# Independent versus joint effects of polygenic or family-based schizophrenia risk in diverse ancestry youth in the ABCD study

**DOI:** 10.1017/S0033291725102304

**Published:** 2025-10-30

**Authors:** Mahnoor Hyat, Jinhan Zhu, Toni A. Boltz, Matthew P. Conomos, Dylan E. Hughes, Alison E. Fohner, Katherine T. Foster, Tim B. Bigdeli, Jennifer K. Forsyth

**Affiliations:** 1 Department of Psychology, University of Washington, Seattle, WA, USA; 2 Stanley Center for Psychiatric Research, Broad Institute of MIT and Harvard, Cambridge, MA, USA; 3 Department of Biostatistics, University of Washington, Seattle, WA, USA; 4 Department of Psychology, University of California, Los Angeles, CA, USA; 5 Department of Epidemiology, University of Washington, WA, USA; 6 Institute for Public Health Genetics, University of Washington, WA, USA; 7 Department of Global Health, University of Washington, WA, USA; 8 Department of Psychiatry and Behavioral Sciences, Institute for Genomics in Health, State University of New York Downstate Health Sciences University, Brooklyn, NY, USA; 9 Department of Veterans Affairs New York Harbor Healthcare System, Brooklyn, NY, USA

**Keywords:** ABCD study, childhood, cognition, early signs, family history, polygenic risk scores (PRS), psychopathology, schizophrenia, symptoms

## Abstract

**Background:**

Subtle behavioral and cognitive symptoms precede schizophrenia (SCZ) and appear in individuals with elevated risk based on polygenic risk scores (SCZ-PRS) and family history of psychosis (SCZ-FH). However, most SCZ-PRS studies focus on European ancestry youth, limiting generalizability. Furthermore, it remains unclear whether SCZ-FH reflects common-variant polygenic risk or broader SCZ liability.

**Methods:**

Using baseline data from the Adolescent Brain Cognitive Development (ABCD) study, we investigated associations of SCZ-FH and SCZ-PRS with cognitive, behavioral, and emotional measures from NIH-Toolbox, Child Behavior Checklist (CBCL), and Kiddie Schedule for Affective Disorders and Schizophrenia (KSADS) for 9,636 children (mean age = 9.92 yrs, 47.4% female), specifically, 5,636 European, 2,093 African, and 1,477 Admixed American ancestry individuals.

**Results:**

SCZ-FH was associated with SCZ-PRS (*b* = 0.05, FDR-*p* = 0.02) and subthreshold psychotic symptoms (*b* = 0.46, FDR-*p* = 0.01) in European youth, higher CBCL scores (*b* range = 0.36–0.6, FDR-*p* < 0.001), and higher odds of multiple internalizing and externalizing disorders (OR = 1.10–1.22, FDR-*p* < 0.001) across ancestries. SCZ-PRS was associated with lower cognition across ancestries (*b* = −0.43, FDR-*p* = 0.02), higher CBCL total problems, anxious/depressed, rule-breaking and aggressive behaviors in European youth (*b* range = 0.16–0.33, FDR-*p* < 0.04), and depressive disorders in Admixed American youth (OR = 1.37, FDR-*p* = 0.02). Results remained consistent when SCZ-PRS and SCZ-FH were jointly modeled. Some SCZ-FH associations weakened when income-to-needs was accounted for, suggesting that SCZ-FH may capture both genetic and environmental influences.

**Conclusions:**

SCZ-FH showed associations with broad psychopathology, while SCZ-PRS was associated with cognition and specific symptoms in European youth. Findings highlight their complementary role in SCZ risk assessment and the need to improve PRS utility across ancestries.

## Introduction

Schizophrenia (SCZ) is a severe psychiatric disorder with substantial personal and societal impact (Chong et al., 2016; Evensen et al., 2016; Kar & Jain, 2016; Laursen, Nordentoft, & Mortensen, 2014). Standard treatments typically begin after full-blown psychosis onset but often fail to restore premorbid levels of functioning. The clinical staging model of SCZ posits that persistent illness reflects late-stage manifestation of disrupted neurodevelopmental processes and that subtle early signs and symptoms may serve as markers of future psychopathology (Forsyth & Lewis, 2017; McGorry et al., 2014). For example, birth cohort studies show that individuals later diagnosed with SCZ exhibit higher rates of childhood cognitive deficits and behavioral and emotional problems (Mollon & Reichenberg, 2018; Riglin et al., 2017; Welham, Isohanni, Jones, & McGrath, 2009). Elevated psychotic-like experiences (PLEs) in childhood, or subthreshold psychotic symptoms, are also associated with later psychotic disorder onset (Healy et al., 2019; Laurens et al., 2007). Similarly, adults with SCZ are more likely to have met criteria for other psychiatric diagnoses, such as anxiety and affective disorders, in childhood and adolescence (Maibing et al., 2015). This suggests that childhood behavioral and clinical signs may help identify individuals at risk for SCZ; however, as most children with such difficulties do not develop SCZ, a clearer understanding of how symptoms relate to underlying etiological processes is essential.

Importantly, genetic factors are known to contribute to the development of SCZ, with twin studies estimating heritability at around 80% (Hilker et al., 2018). Given elevated rates of SCZ among individuals with a family history of psychosis (SCZ-FH) (Cheng et al., 2018; Goldstein, Buka, Seidman, & Tsuang, 2010), SCZ-FH has long served as a proxy for genetic risk and a means of studying potential antecedents of SCZ in at-risk youth (Díaz-Castro et al., 2021; Niemi, Suvisaari, Tuulio-Henriksson, & Lönnqvist, 2003). For example, offspring of individuals with SCZ show higher rates of lifetime psychiatric disorders and cognitive impairments (Erlenmeyer-Kimling et al., 2000; Keshavan, 2009; Sanchez-Gistau et al., 2015). However, the extent to which SCZ-FH captures genetically specific versus broader risk factors for SCZ is unclear. Furthermore, many individuals who develop SCZ do not have an immediate family member with the disorder, raising questions about the generalizability of markers identified in youth with SCZ-FH to those who develop SCZ without known familial risk.

Fortunately, advancements in genetics, especially the advent of large-scale genome-wide association studies (GWAS), have enabled the identification of robust associations between psychiatric disorders and specific genetic risk variants (Schizophrenia Working Group of the Psychiatric Genomics Consortium, 2014). The most recent SCZ GWAS, based on 76,755 patients and 243,649 controls, identified 287 loci significantly associated with SCZ (Trubetskoy et al., 2022). This enables the computation of polygenic risk scores (PRS), which capture an individual’s genetic risk for the disorder as a weighted sum of risk alleles and can be tested for association with childhood phenotypes to identify genetically-mediated antecedents of SCZ. SCZ-PRS, explaining up to 7.3–7.7% of the variance in SCZ case–control status (Legge et al., 2019; Trubetskoy et al., 2022), have been associated with negative symptoms and cognitive impairments in childhood, but findings for depressive and psychotic-like experiences are mixed (Jones et al., 2016; Mistry et al., 2018; Nivard et al., 2017).

Beyond equivocal links between SCZ-PRS and childhood psychopathology, a key challenge remains in developing generalizable SCZ-PRS for prediction across populations. This is due to an over-representation of European ancestry individuals in existing GWAS, which reduces PRS accuracy in diverse populations because of differences in allele frequency and linkage disequilibrium (LD) patterns (Kachuri et al., 2024). However, recent efforts to diversify GWAS samples (e.g., Bigdeli et al., 2020) and the development of advanced PRS construction methods like PRS-CSx, which refine allele effect size estimates by integrating multi-ancestry GWAS summary statistics and ancestry-matched LD panels (Ruan et al., 2022), are improving cross-population PRS accuracy and supporting more equitable risk assessment.

To investigate whether associations between early signs and symptoms and SCZ-FH or SCZ-PRS represent independent or overlapping aspects of SCZ risk, and to assess the generalizability of these associations across genetic ancestries, the current study used data from the Adolescent Brain Cognitive Development (ABCD) Study to examine how SCZ-FH and SCZ-PRS relate to cognitive, behavioral, and emotional functioning during childhood. The ABCD Study is the largest nationally representative, longitudinal study of brain development in the US and includes genetic, behavioral, and clinical information. Previous studies in ABCD found higher SCZ-PRS linked to worse cognitive functioning, greater attentional variability, and more psychotic-like experiences, but showed mixed results for internalizing and externalizing problems in childhood (Chang et al., 2024; Loughnan et al., 2022; Wainberg, Jacobs, Voineskos, & Tripathy, 2022). However, these studies focused on European ancestry youth and/or used methods for PRS construction that were not optimized for cross-ancestry PRS accuracy. By incorporating SCZ GWAS summary statistics from diverse populations and using PRS-CSx to optimize SCZ-PRS accuracy across ancestries, this study refines prior investigations for diverse youth to determine whether integrating SCZ-FH and SCZ-PRS in combined models yields incremental utility for identifying early SCZ risk markers.

## Methods and materials

### Sample

This study utilized baseline data from the ABCD study (Release 4.0), which enrolled 11,880 children aged 9–10 years old across 21 sites to reflect the sociodemographic diversity of the US population (Yang & Jernigan, n.d.), to investigate SCZ-related signs and symptoms at the earliest available time point. Analyses investigated youth of European- (EUR; *n* = 5,626), African- (AFR; *n* = 2,093), or Admixed (AMR; *n* = 1,477) American-like ancestry (47.4% female; mean age = 9.92 years), as determined by genetic similarity to HapMap3 reference populations, as these groups were sufficiently powered for investigation.

### Measures

#### SCZ risk

SCZ-FH was assessed by the ABCD team using the caregiver-reported Family History Assessment Module Screener (FHAM-S), which collected information on family members’ experiences with depression, mania, hallucinations, and related conditions (Barch et al., 2018). SCZ-FH was determined through the question: *“Has ANY blood relative of your child ever had a period lasting six months when they saw visions or heard voices or thought people were spying on them or plotting against them?”* We created a continuous weighted SCZ-FH measure by summing the number of affected relatives weighted by degree of relatedness (i.e. 0.5 for first-degree and 0.25 for second-degree relatives), and then z-score standardized it. Given the low occurrence of family history endorsement, one second-degree relative with psychosis corresponded to a z-score of 1.76, and one first-degree relative corresponded to a z-score of 3.77. An alternative SCZ-FH score that adjusted for the total number of first- and second-degree relatives (SCZ-FH prop) was also generated. However, because this proportional score showed weaker associations with SCZ-PRS, the weighted count measure was used for the primary analyses. See Supplementary Materials for details.

SCZ-PRS were derived from imputed genotyping data that underwent rigorous quality control by the ABCD team following the Ricopili pipeline (Lam et al., 2020; Wainberg et al., 2022). Imputation was completed using the Trans-Omics for Precision Medicine (TOPMed) reference panel version r2 (Das et al., 2016; Fan, Loughnan, Wilson, and Hewitt, 2023; Loh et al., 2016; Taliun et al., 2021). Following ABCD recommendations, we excluded data from plate 461 due to poor quality. Only high-quality imputed variants (i.e. R2 > 0.8) with minor allele frequency (MAF) > 0.01 were retained for PRS construction. To minimize confounding by the complex LD structure of the major histocompatibility complex (MHC) region, only one SNP in the region with the strongest association to SCZ was retained (Trubetskoy et al., 2022).

Before deriving SCZ-PRS, PRS-CSx was employed to refine SNP effect size estimates by integrating summary statistics from multiple GWAS and accounting for LD patterns across populations. This method was chosen due to its demonstrated accuracy in improving cross-ancestry PRS accuracy compared to other methods (Ruan et al., 2022). Specifically, summary statistics were obtained from the largest GWAS of SCZ, in which EUR-like ancestry individuals were over-represented (Trubetskoy et al., 2022), as well as smaller GWAS focused on AMR and AFR-like ancestry individuals (Bigdeli et al., 2021). The PRS-CSx output included a meta-analysis file, which provided refined SNP effect sizes across populations using an inverse-variance-weighted meta-analysis of population-specific posterior effect size estimates after incorporating 1000 Genomes Project LD reference panels. It also provided ancestry-specific SNP effect size estimates for each GWAS. PRS-CSx meta-analyzed SNP estimates were used to construct SCZ-PRS for AMR-like and AFR-like ancestry individuals, whereas SNP effect size estimates derived from the predominantly European ancestry GWAS were used for EUR-like individuals. This is because prior evidence suggests meta-analyzed effect size estimates improve prediction for non-EUR-like individuals, whereas ancestry-specific estimates offer greater accuracy for EUR-like individuals (Ruan et al., 2022). SCZ-PRS were generated in PLINK and z-score standardized within each ancestry group.

#### Behavioral and cognitive signs and symptoms

##### Dimensional assessments

Cognitive functioning across multiple domains was assessed with the NIH Toolbox (Gershon et al., 2013; Weintraub et al., 2013). A winsorized, age-corrected composite cognition score, summarizing performance across tasks, was used for analysis. Emotional and behavioral problems in children over the past 6 months were assessed via the parent-report Child Behavior Checklist (CBCL) (Achenbach, 1991). The CBCL total composite score, along with scores for the eight primary subscales: anxious/depressed, withdrawn/depressed, somatic complaints, social problems, thought problems, attention problems, rule-breaking behavior, and aggressive behavior, were used for analysis. Psychotic-like experiences (PLEs) were assessed via the Prodromal Questionnaire – Brief Child Version (PQ-BC), which is a 21-item scale assessing occurrences of PLEs and the associated level of distress. PLEs were operationalized as the sum of the number of endorsed items weighted by distress level (PQ-BC Distress) (Chang et al., 2024).

##### Diagnostic assessments

Lifetime history of depressive disorders, anxiety disorders, conduct disorder, and ADHD were derived from the caregiver self-administered Kiddie Schedule for Affective Disorders and Schizophrenia (KSADS-COMP) (Kaufman et al., 1997; Townsend et al., 2020). To facilitate comparison with previously published methods for deriving ADHD diagnoses in ABCD (Cordova et al., 2022), we generated an additional ADHD variable using a t-score of ≥65 on the CBCL Attention Problems scale (i.e. the threshold for clinical-level attention problems). See Supplementary Methods for details on measures, including *n*s per measure and endorsement rates.

### Statistical analysis

#### Ancestry and genetic relatedness variables

To create ancestrally homogeneous groups of subjects, we conducted principal components analysis (PCA) using high-quality SNPs from ABCD merged with HapMap3 data as the reference (The International HapMap 3 Consortium, 2010). Ancestry representative PCs were generated following an iterative procedure optimized for samples from diverse groups with familial relatedness (Conomos et al., 2016), using PC-AiR for PC calculation and PC-Relate for kinship coefficient estimation, implemented in the GENESIS R package (Conomos, Miller, & Thornton, 2015; Conomos, Reiner, Weir, & Thornton, 2016). We utilized the top eight PCs as inputs for a Random Forest classifier to categorize participants into ancestry groups based on genetic similarity, using HapMap3 super-population labels (Alexander & Lange, 2011). See Supplementary Figure S1 for distributions across PCs.

#### Association testing

Linear and logistic mixed models were implemented using the GENESIS R package to examine associations between SCZ-FH or SCZ-PRS and the continuous and dichotomous outcomes, respectively (Gogarten et al., 2019). Associations were first assessed in independent models (i.e. dependent variable ~ SCZ-FH/PRS + covariates), followed by a joint model including both SCZ-FH and SCZ-PRS (i.e. dependent variable ~ SCZ-FH + SCZ-PRS + covariates) to refine coefficient estimates for each risk measure, while accounting for the other. We conducted analyses separately for the three ancestries (EUR-only, AFR-only, and AMR-only), followed by a combined analysis using within-ancestry z-scored SCZ-PRS (cross-ancestry). The fixed-effect covariates included sex, age, study site, and ancestry principal components, while genetic correlation was accounted for as a random effect through the genetic relatedness matrix (GRM). P-values across dimensional measure models were corrected for multiple testing using false discovery rate (FDR) correction for the full sample and within each ancestry, across both risk SCZ measures (i.e. 20 models per ancestry, 10 SCZ-FH, 10 SCZ-PRS). p-Values for diagnostic outcomes were similarly FDR-corrected for the full sample and within each ancestry, across both risk SCZ measures (i.e. 12 models per ancestry, 6 SCZ-FH, 6 SCZ-PRS). Proportions of variance explained (PVE) by each risk factor were estimated using score statistics (Hu et al., 2021). We also conducted sensitivity analysis incorporating income-to-needs as an additional covariate to determine whether poverty-related factors influence SCZ-FH & SCZ-PRS associations.

## Results

**Figure 1.**
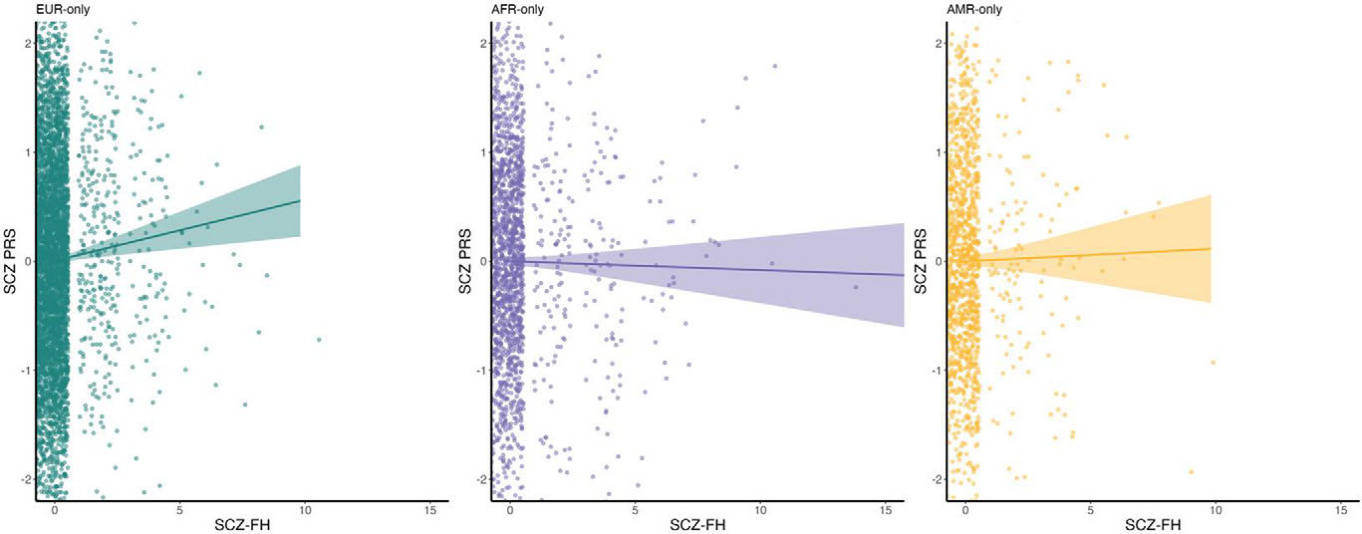
Scatterplot of associations between schizophrenia polygenic risk scores (SCZ-PRS) and family history of psychosis (SCZ-FH) for each of the three ancestries. EUR-only refers to European ancestry youth; AFR-only refers to African ancestry youth; AMR-only refers to American Admixed ancestry youth.

We first assessed associations between SCZ-FH and SCZ-PRS; as expected, they showed a positive relationship across ancestries (Figure 1). This association was significant for the EUR-only group (*b* = 0.05, *p* = 0.01, FDR *p* = 0.02), but not in the cross-ancestry analysis (*b =* 0.01, *p* = 0.27, FDR *p* = 0.53), or for AFR-only (*b =* 0.01, *p* = 0.53, FDR *p* = 0.53) and AMR-only models (*b =* 0.02, *p* = 0.51, FDR *p* = 0.53).

### Independent associations between SCZ risk measures and phenotypes

Table 1 and Supplementary Figure S2A summarizes the independent associations between SCZ-FH and SCZ-PRS and childhood dimensional phenotypes of interest.

**Table 1.** Independent associations of schizophrenia polygenic risk scores (SCZ-PRS) and family history of psychosis (SCZ-FH) with dimensional, total cognitive score derived from the NIH-Toolbox, Child Behavior Checklist (CBCL) scores, and prodromal questionnaire (PQB) scores

Ancestry	Genetic risk measure	Precursor	*B*	SE	95% CI	Nominal P	FDR P	PVE (%)
All	Family history	NIH Total cognitive score	−0.13	0.18	−0.47 to 0.22	0.469	0.607	0.01
		CBCL – Total	1.12	0.12	0.88 to 1.36	<0.001	**<0.001**	0.92
		CBCL – Anxious Depressed	0.40	0.06	0.28 to 0.53	<0.001	**<0.001**	0.44
		CBCL – Withdrawn	0.43	0.06	0.31 to 0.55	<0.001	**<0.001**	0.51
		CBCL – Somatic	0.43	0.07	0.30 to 0.56	<0.001	**<0.001**	0.47
		CBCL – Social	0.49	0.05	0.40 to 0.59	<0.001	**<0.001**	1.03
		CBCL – Thought	0.60	0.06	0.47 to 0.72	<0.001	**<0.001**	0.93
		CBCL – Attention	0.53	0.07	0.40 to 0.67	<0.001	**<0.001**	0.68
		CBCL – Rule Breaking	0.36	0.05	0.26 to 0.46	<0.001	**<0.001**	0.50
		CBCL – Aggressive	0.48	0.06	0.37 to 0.60	<0.001	**<0.001**	0.71
		PQB – Distress Score	0.43	0.11	0.21 to 0.65	<0.001	**<0.001**	0.16
	SCZ PRS	NIH Total Cognitive Score	−0.43	0.17	−0.77 to −0.10	0.011	**0.023**	0.07
		CBCL – Total	0.08	0.12	−0.16 to 0.31	0.520	0.636	0.00
		CBCL – Anxious Depressed	0.07	0.06	−0.05 to 0.20	0.242	0.354	0.01
		CBCL – Withdrawn	0.01	0.06	−0.11 to 0.13	0.849	0.889	0.00
		CBCL – Somatic	−0.09	0.06	−0.22 to 0.04	0.170	0.282	0.02
		CBCL – Social	0.01	0.05	−0.09 to 0.11	0.843	0.889	0.00
		CBCL – Thought	0.06	0.06	−0.06 to 0.19	0.320	0.440	0.01
		CBCL – Attention	0.00	0.07	−0.13 to 0.13	0.977	0.977	0.00
		CBCL – Rule Breaking	0.07	0.05	−0.03 to 0.17	0.179	0.282	0.02
		CBCL – Aggressive	0.02	0.06	−0.09 to 0.14	0.690	0.799	0.00
		PQB – Distress Score	0.15	0.11	−0.06 to 0.37	0.168	0.282	0.02
European	Family History	NIH Total Cognitive Score	−0.44	0.28	−0.99 to 0.11	0.118	0.145	0.04
		CBCL – Total	0.97	0.19	0.61 to 1.34	<0.001	**<0.001**	0.48
		CBCL – Anxious Depressed	0.27	0.10	0.07 to 0.48	0.010	**0.017**	0.12
		CBCL – Withdrawn	0.25	0.09	0.07 to 0.44	0.008	**0.015**	0.13
		CBCL – Somatic	0.42	0.10	0.22 to 0.62	<0.001	**<0.001**	0.29
		CBCL – Social	0.40	0.08	0.25 to 0.55	<0.001	**<0.001**	0.47
		CBCL – Thought	0.65	0.10	0.44 to 0.85	<0.001	**<0.001**	0.70
		CBCL – Attention	0.61	0.10	0.41 to 0.81	<0.001	**<0.001**	0.63
		CBCL – Rule Breaking	0.34	0.08	0.19–0.49	<0.001	**<0.001**	0.36
		CBCL – Aggressive	0.40	0.09	0.22–0.57	<0.001	**<0.001**	0.34
		PQB – Distress Score	0.46	0.16	0.16–0.77	0.003	**0.006**	0.16
	SCZ PRS	NIH Total Cognitive Score	−0.78	0.22	−1.21 – –0.34	<0.001	**0.0013**	0.22
		CBCL – Total	0.33	0.15	0.04–0.62	0.026	**0.038**	0.09
		CBCL – Anxious Depressed	0.21	0.08	0.04–0.37	0.013	**0.022**	0.11
		CBCL – Withdrawn	0.09	0.08	−0.06 to 0.24	0.238	0.280	0.02
		CBCL – Somatic	−0.08	0.08	−0.24 to 0.08	0.342	0.380	0.02
		CBCL – Social	0.02	0.06	−0.09 to 0.14	0.683	0.680	0.00
		CBCL – Thought	0.13	0.08	−0.03 to 0.29	0.102	0.130	0.05
		CBCL – Attention	0.07	0.08	−0.09 to 0.23	0.384	0.400	0.01
		CBCL – Rule Breaking	0.21	0.06	0.09 to 0.33	0.001	**0.001**	0.21
		CBCL – Aggressive	0.16	0.07	0.02 to 0.30	0.023	**0.036**	0.09
		PQB – Distress Score	0.22	0.12	−0.03 to 0.46	0.083	0.114	0.05
African	Family History	NIH Total Cognitive Score	−0.03	0.27	−0.55 to 0.49	0.905	0.946	0.00
		CBCL – Total	1.26	0.20	0.87 to 1.66	<0.001	**<0.001**	1.85
		CBCL – Anxious Depressed	0.46	0.09	0.29 to 0.64	<0.001	**<0.001**	1.29
		CBCL – Withdrawn	0.52	0.10	0.32 to 0.72	<0.001	**<0.001**	1.24
		CBCL – Somatic	0.41	0.10	0.21 to 0.60	<0.001	**<0.001**	0.81
		CBCL – Social	0.57	0.08	0.41 to 0.74	<0.001	**<0.001**	2.20
		CBCL – Thought	0.61	0.10	0.41 to 0.80	<0.001	**<0.001**	1.69
		CBCL – Attention	0.45	0.11	0.23 to 0.68	<0.001	**<0.001**	0.77
		CBCL – Rule Breaking	0.36	0.10	0.17 to 0.56	<0.001	**<0.001**	0.62
		CBCL – Aggressive	0.53	0.10	0.33 to 0.73	<0.001	**<0.001**	1.24
		PQB – Distress Score	0.33	0.20	−0.07 to 0.72	0.109	0.239	0.12
	SCZ PRS	NIH Total Cognitive Score	0.33	0.42	−0.49 to 1.15	0.431	0.678	0.03
		CBCL – Total	−0.17	0.33	−0.82 to 0.48	0.600	0.744	0.01
		CBCL – Anxious Depressed	−0.06	0.15	−0.35 to 0.23	0.676	0.744	0.01
		CBCL – Withdrawn	−0.19	0.17	−0.52 to 0.14	0.267	0.500	0.06
		CBCL – Somatic	−0.07	0.16	−0.39 to 0.25	0.670	0.744	0.01
		CBCL – Social	−0.01	0.14	−0.28 to 0.26	0.946	0.946	0.00
		CBCL – Thought	−0.07	0.17	−0.40 to 0.25	0.657	0.744	0.01
		CBCL – Attention	0.13	0.18	−0.23 to 0.49	0.475	0.697	0.02
		CBCL – Rule Breaking	−0.18	0.17	−0.50 to 0.14	0.273	0.500	0.06
		CBCL – Aggressive	−0.13	0.17	−0.47 to 0.20	0.431	0.678	0.03
		PQB – Distress Score	0.18	0.34	−0.48 to 0.84	0.598	0.744	0.01
American Admixed	Family History	NIH Total Cognitive Score	0.27	0.43	−0.58 to 1.12	0.529	0.645	0.03
		CBCL – Total	0.94	0.30	0.35 to 1.54	0.002	**0.007**	0.66
		CBCL – Anxious Depressed	0.49	0.16	0.19 to 0.81	0.002	**0.007**	0.67
		CBCL – Withdrawn	0.56	0.17	0.23 to 0.90	0.001	**0.005**	0.75
		CBCL – Somatic	0.45	0.17	0.12 to 0.79	0.008	**0.019**	0.49
		CBCL – Social	0.45	0.13	0.20 to 0.71	0.001	**0.005**	0.82
		CBCL – Thought	0.44	0.15	0.14 to 0.74	0.004	**0.012**	0.57
		CBCL – Attention	0.56	0.16	0.24 to 0.89	0.001	**0.005**	0.79
		CBCL – Rule Breaking	0.34	0.12	0.10 to 0.59	0.005	**0.015**	0.53
		CBCL – Aggressive	0.52	0.14	0.25 to 0.81	<0.001	**0.005**	0.92
		PQB – Distress Score	0.69	0.31	0.08 to 1.32	0.027	0.060	0.33
	SCZ PRS	NIH Total Cognitive Score	0.11	0.45	−0.76 – 0.99	0.803	0.844	0.00
		CBCL – Total	0.33	0.31	−0.29 to 0.94	0.296	0.445	0.07
		CBCL – Anxious Depressed	0.17	0.16	−0.15 to 0.49	0.304	0.445	0.07
		CBCL – Withdrawn	0.24	0.18	−0.11 to 0.58	0.176	0.322	0.13
		CBCL – Somatic	0.14	0.18	−0.21 to 0.49	0.428	0.553	0.04
		CBCL – Social	0.21	0.13	−0.05 to 0.47	0.109	0.219	0.18
		CBCL – Thought	0.15	0.16	−0.16 to 0.46	0.345	0.474	0.06
		CBCL – Attention	−0.04	0.17	−0.38 to 0.29	0.806	0.844	0.00
		CBCL – Rule Breaking	0.16	0.13	−0.09 to 0.41	0.213	0.360	0.11
		CBCL – Aggressive	0.09	0.15	−0.21 to 0.38	0.557	0.645	0.02
		PQB – Distress Score	0.04	0.32	−0.59 to 0.68	0.890	0.890	0.00

*Note:* FDR P refers to the false discovery rate–corrected *p* value (bold values indicate FDR-corrected p<0.05); PVE refers to the percentage of variance explained.

For NIH-TB total cognition, no significant associations were observed with SCZ-FH in the cross-ancestry analysis, nor in the EUR-only, AFR-only, and AMR-only analyses. Conversely, greater SCZ-FH was associated with CBCL total problems in the cross-ancestry analysis (*b* = 1.12, *p* < 0.001, FDR *p* < 0.001) and within the three ancestries (*b* range = 0.94–1.26, *p* < =0.002, FDR *p* < 0.007). SCZ-FH was also associated with all eight CBCL subscales in the cross-ancestry analysis and within the three groups (*b* range = 0.25–0.65, *p* < 0.001, FDR *p* < 0.001). Additionally, individuals with greater SCZ-FH had higher PQB distress scores in the cross-ancestry (*b* = 0.43, *p* < 0.001, FDR *p* < 0.001) and EUR-only analyses (*b* = 0.46, *p* = 0.003, FDR *p* = 0.006); this association was only nominally significant in the AMR-only analysis and null in the AFR-only analysis.

Exploratory analyses using a measure of SCZ-FH adjusted for the total number of first- and second-degree relatives (SCZ-FH-prop) did not show an association with SCZ-PRS. However, associations for SCZ-FH-prop with dimensional phenotypes were similar. The association between total cognition and SCZ-FH-prop was also significant in EUR-only analyses, but not for other groups. See Supplementary Materials for details.

Higher SCZ-PRS was associated with significantly lower total cognition scores in the cross-ancestry (*b* = −0.43, *p* = 0.01 FDR *p* = 0.02) and EUR-only analyses (*b* = −0.78, *p* < 0.001, FDR *p* = 0.001), but not in the AFR-only or AMR-only analyses. SCZ-PRS was not significantly associated with CBCL total problems (*b* = 0.08 *p* = 0.52, FDR *p* = 0.64) or any of the 8 subscale scores in the cross-ancestry analysis (*b* range = −0.09–0.07, *p* = 0.17–0.98, FDR *p* = 0.28–0.98). However, in the EUR-only analyses, higher SCZ-PRS was associated with higher CBCL total problems (*b* = 0.33, *p* = 0.03, FDR *p* = 0.04), anxious/depressed symptoms (*b* = 0.21, *p* = 0.01, FDR *p* = 0.02), rule-breaking (*b* = 0.21, *p* = 0.001, FDR *p* = 0.001) and aggressive behaviors (*b* = 0.16, *p* = 0.02, FDR *p* = 0.04). SCZ-PRS and PQB Distress scores were not associated in the cross-ancestry analysis (*b* = 0.15, *p* = 0.17, FDR *p* = 0.28) or within any of the three ancestries (*p*s > .05).

Primary analyses for psychopathology focused on dimensional symptoms, given relatively low rates of psychiatric disorders at this young age. However, exploratory analyses of clinical diagnoses in childhood indicated that SCZ-FH was associated with a lifetime history of diagnoses, including depressive, conduct, and anxiety disorders as well as ADHD in the cross-ancestry analysis. SCZ-FH was also associated with multiple diagnoses in ancestry group-specific analyses. SCZ-PRS was not significantly associated with lifetime history of diagnoses in the cross-ancestry analysis or within individual ancestry groups for most disorders, except for depressive disorders in AMR-only youth. See Supplementary Results for details.

### Joint modeling of SCZ risk measures with phenotypes

**Figure 2.**
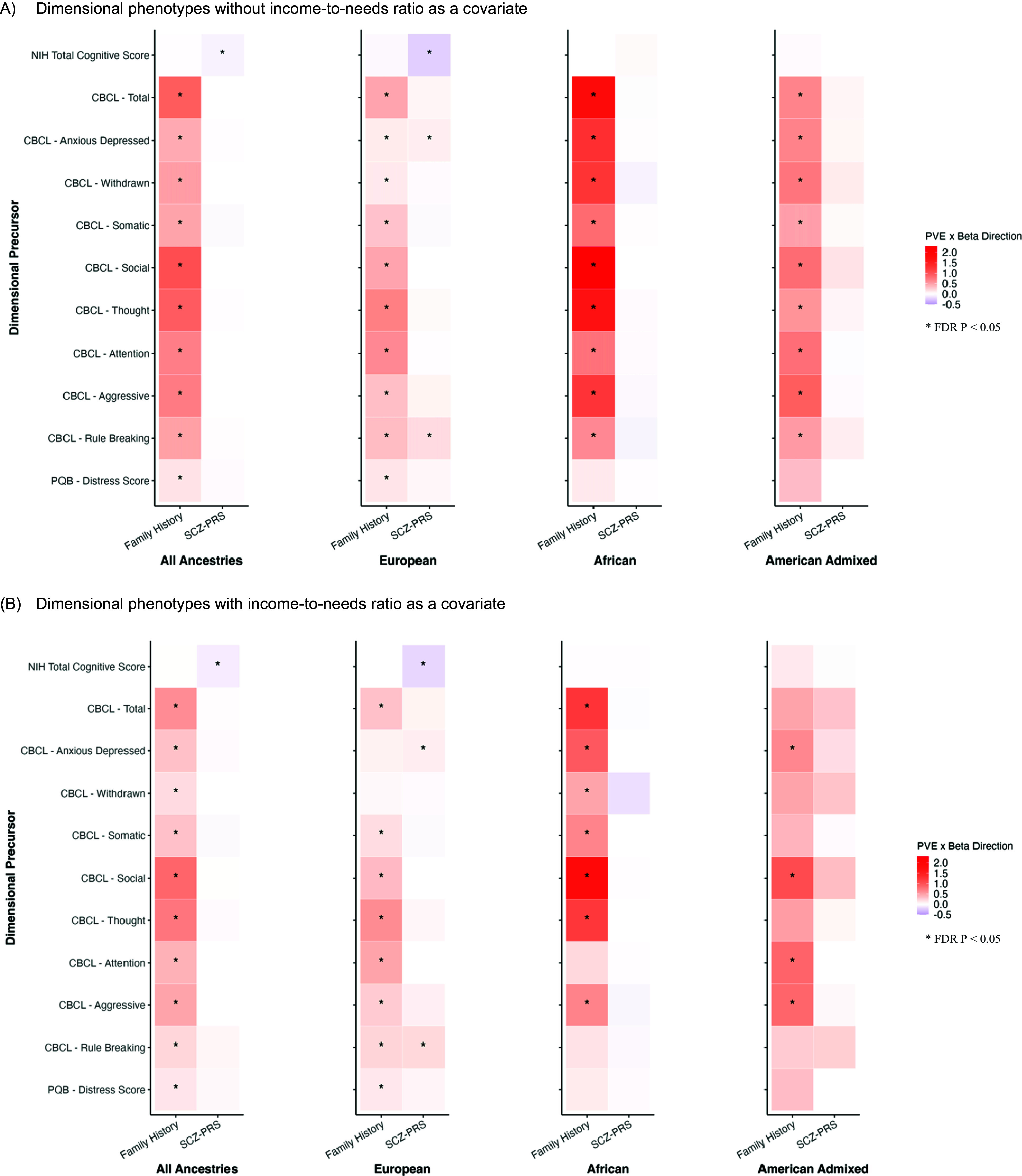
Heatmap of joint associations of schizophrenia polygenic risk scores (SCZ-PRS) and family history of psychosis (SCZ-FH) with total cognitive score derived from the NIH-Toolbox, Child Behavior Checklist (CBCL) scores, and prodromal questionnaire (PQB) scores (A) without income-to-needs ratio as a covariate and (B) with income-to-needs ratio as a covariate. Heatmap shows associations of one genetic risk measure (i.e. SCZ-PRS or SCZ-FH) while adjusting for the other. FDR P refers to the false discovery rate-corrected *p-value.*

Table 2 and Figure 2A summarize associations between the two SCZ risk measures and dimensional phenotypes when modeled jointly. These joint model associations closely mirrored patterns observed in the models when each SCZ risk measure was tested separately.

**Table 2.** Joint model associations of schizophrenia polygenic risk scores (SCZ-PRS) and family history of psychosis (SCZ-FH) with dimensional, total cognitive score derived from the NIH-Toolbox, Child Behavior Checklist (CBCL) scores, and prodromal questionnaire (PQB) scores

Ancestry	Precursor	Family history of psychosis (joint model)						SCZ PRS (joint model)						Joint PVE
		*B*	*SE*	*95% CI*	*Nominal P*	*FDR P*	*PVE*	*B*	*SE*	*95% CI*	*Nominal P*	*FDR P*	*PVE*	
All	NIH total cognitive score	−0.12	0.18	−0.47 to 0.23	0.493	0.638	0.01	−0.43	0.17	−0.77 to –0.10	0.012	**0.024**	0.07	0.08
	CBCL – Total	1.12	0.12	0.88 to 1.36	<0.001	**<0.001**	0.92	0.06	0.12	−0.17 to 0.30	0.596	0.729	0.00	0.93
	CBCL – Anxious Depressed	0.40	0.06	0.28 to 0.53	<0.001	**<0.001**	0.44	0.07	0.06	−0.05 to 0.19	0.277	0.406	0.01	0.45
	CBCL – Withdrawn	0.43	0.06	0.31 to 0.55	<0.001	**<0.001**	0.51	0.01	0.06	−0.12 to 0.13	0.921	0.944	0.00	0.51
	CBCL – Somatic	0.43	0.07	0.30 to 0.56	<0.001	**<0.001**	0.47	−0.09	0.06	−0.22 to 0.03	0.144	0.264	0.02	0.49
	CBCL – Social	0.49	0.05	0.40 to 0.59	<0.001	**<0.001**	1.03	0.00	0.05	−0.09 to 0.10	0.944	0.944	0.00	1.03
	CBCL – Thought	0.60	0.06	0.47 to 0.72	<0.001	**<0.001**	0.93	0.06	0.06	−0.07 to 0.18	0.380	0.523	0.01	0.94
	CBCL – Attention	0.53	0.07	0.40 to 0.67	<0.001	**<0.001**	0.68	−0.01	0.07	−0.14 to 0.12	0.897	0.944	0.00	0.68
	CBCL – Rule Breaking	0.36	0.05	0.25 to 0.46	<0.001	**<0.001**	0.50	0.07	0.05	−0.04 to 0.17	0.208	0.327	0.02	0.52
	CBCL – Aggressive	0.48	0.06	0.37 to 0.60	<0.001	**<0.001**	0.71	0.02	0.06	−0.10 to 0.13	0.767	0.888	0.00	0.71
	PQB – Distress Score	0.43	0.11	0.21 to 0.65	<0.001	**<0.001**	0.16	0.15	0.11	−0.07 to 0.37	0.184	0.311	0.02	0.18
European	NIH Total Cognitive Score	−0.40	0.28	−0.95 – 0.15	0.153	0.198	0.04	−0.76	0.22	−1.20 to –0.33	<0.001	**0.002**	0.22	0.26
	CBCL – Total	0.96	0.19	0.59–1.32	<0.001	**<0.001**	0.46	0.30	0.15	0.01 to 0.59	0.042	0.061	0.07	0.55
	CBCL – Anxious Depressed	0.26	0.10	0.06 to 0.47	0.013	**0.023**	0.11	0.20	0.08	0.03 to 0.36	0.017	**0.029**	0.10	0.22
	CBCL – Withdrawn	0.25	0.09	0.06 to 0.43	0.009	**0.018**	0.12	0.08	0.08	−0.07 to 0.23	0.283	0.312	0.02	0.15
	CBCL – Somatic	0.42	0.10	0.22 to 0.62	<0.001	**<0.001**	0.30	−0.09	0.08	−0.25 to 0.07	0.263	0.305	0.02	0.32
	CBCL – Social	0.40	0.08	0.25 to 0.55	<0.001	**<0.001**	0.47	0.01	0.06	−0.11 to 0.13	0.841	0.841	0.00	0.47
	CBCL – Thought	0.64	0.10	0.44 to 0.84	<0.001	**<0.001**	0.69	0.11	0.08	−0.05 to 0.27	0.166	0.202	0.03	0.74
	CBCL – Attention	0.61	0.10	0.41 to 0.81	<0.001	**<0.001**	0.62	0.05	0.08	−0.11 to 0.21	0.527	0.552	0.01	0.64
	CBCL – Rule Breaking	0.33	0.08	0.18 to 0.48	<0.001	**<0.001**	0.34	0.20	0.06	0.08–0.32	0.001	**0.003**	0.19	0.55
	CBCL – Aggressive	0.39	0.09	0.21 to 0.57	<0.001	**<0.001**	0.33	0.15	0.07	0.01 to 0.29	0.035	0.056	0.08	0.42
	PQB – Distress Score	0.45	0.16	0.15 to 0.76	0.004	**0.008**	0.15	0.20	0.12	−0.04 to 0.44	0.107	0.147	0.05	0.20
African	NIH Total Cognitive Score	−0.04	0.27	−0.56 to 0.48	0.891	0.891	0.00	0.33	0.42	−0.49 to 1.15	0.430	0.676	0.03	0.03
	CBCL – Total	1.26	0.20	0.87 to 1.66	<0.001	**<0.001**	1.85	−0.20	0.33	−0.85 to 0.44	0.537	0.690	0.02	1.87
	CBCL – Anxious Depressed	0.46	0.09	0.29 to 0.64	<0.001	**<0.001**	1.29	−0.07	0.15	−0.36 to 0.21	0.625	0.690	0.01	1.30
	CBCL – Withdrawn	0.52	0.10	0.32 to 0.72	<0.001	**<0.001**	1.25	−0.20	0.17	−0.52 to 0.13	0.235	0.458	0.07	1.31
	CBCL – Somatic	0.41	0.10	0.22 to 0.60	<0.001	**<0.001**	0.82	−0.08	0.16	−0.39 to 0.24	0.628	0.690	0.01	0.82
	CBCL – Social	0.57	0.08	0.41 to 0.74	<0.001	**<0.001**	2.20	−0.02	0.14	−0.29 to 0.25	0.871	0.891	0.00	2.20
	CBCL – Thought	0.61	0.10	0.41 to 0.81	<0.001	**<0.001**	1.69	−0.09	0.17	−0.41 to 0.24	0.596	0.690	0.01	1.70
	CBCL – Attention	0.45	0.11	0.23 to 0.67	<0.001	**<0.001**	0.77	0.12	0.18	−0.24 to 0.48	0.508	0.690	0.02	0.79
	CBCL – Rule Breaking	0.37	0.10	0.17 to 0.56	<0.001	**<0.001**	0.62	−0.19	0.16	−0.51 to 0.13	0.250	0.458	0.06	0.68
	CBCL – Aggressive	0.53	0.10	0.33 to 0.73	<0.001	**<0.001**	1.24	−0.15	0.17	−0.48 to 0.18	0.387	0.655	0.04	1.27
	PQB – Distress Score	0.32	0.20	−0.07 to 0.72	0.110	0.243	0.12	0.17	0.34	−0.49 to 0.83	0.611	0.690	0.01	0.14
American Admixed	NIH Total Cognitive Score	0.27	0.43	−0.57 to 1.11	0.532	0.650	0.03	0.11	0.45	−0.77 to 0.98	0.809	0.848	0.00	0.03
	CBCL – Total	0.93	0.30	0.34 to 1.52	0.002	**0.007**	0.65	0.31	0.31	−0.30 to 0.92	0.321	0.482	0.07	0.73
	CBCL – Anxious Depressed	0.49	0.16	0.18 to 0.80	0.002	**0.007**	0.66	0.16	0.16	−0.16 to 0.48	0.329	0.482	0.06	0.73
	CBCL – Withdrawn	0.55	0.17	0.22 to 0.88	0.001	**0.005**	0.74	0.23	0.18	−0.12 to 0.57	0.193	0.354	0.11	0.86
	CBCL – Somatic	0.45	0.17	0.12 to 0.78	0.008	**0.019**	0.48	0.13	0.18	−0.21 to 0.48	0.455	0.589	0.04	0.52
	CBCL – Social	0.44	0.13	0.19 to 0.69	0.001	**0.005**	0.81	0.20	0.13	−0.06 to 0.46	0.123	0.246	0.16	0.98
	CBCL to Thought	0.43	0.15	0.14 to 0.73	0.004	**0.013**	0.56	0.14	0.16	−0.17 to 0.45	0.371	0.510	0.05	0.62
	CBCL – Attention	0.56	0.16	0.24 to 0.88	0.001	**0.005**	0.79	−0.05	0.17	−0.39 to 0.28	0.758	0.833	0.01	0.79
	CBCL – Rule Breaking	0.34	0.12	0.10 to 0.58	0.006	**0.016**	0.52	0.15	0.13	−0.10 to 0.40	0.230	0.389	0.10	0.62
	CBCL – Aggressive	0.52	0.14	0.24 to 0.80	<0.001	**0.005**	0.91	0.08	0.15	−0.21 to 0.37	0.599	0.693	0.02	0.94
	PQB – Distress Score	0.69	0.31	0.08 to 1.30	0.027	0.060	0.33	0.03	0.32	−0.60 to 0.67	0.919	0.919	0.00	0.33

*Note:* FDR P refers to the false discovery rate–corrected *p* value (bold values indicate FDR-corrected p<0.05); PVE refers to the percentage of variance explained.

SCZ-FH remained unassociated with total cognition in the cross-ancestry analysis (*b* = −0.12, *p* = 0.49, FDR *p* = 0.64) or within individual ancestries, while higher SCZ-PRS was associated with lower cognitive scores in cross-ancestry (*b* = −0.43, *p* = 0.01, FDR *p* = 0.024) and EUR-only analyses (*b* = −0.76, *p* < 0.001, FDR *p* = 0.002), after adjusting for SCZ-FH.

The joint models showed strong associations between SCZ-FH and CBCL total problems in the cross-ancestry analysis (*b* = 1.12, *p* < 0.001, FDR *p* < 0.001) and within ancestry groups, as well as with the 8 CBCL subscales in the cross-ancestry (*b* range = 0.36–0.60, *p* < 0.001, FDR *p* < 0.001), EUR-only, AFR-only and AMR-only analyses, after adjusting for SCZ-PRS. SCZ-PRS was not associated with CBCL total problems (*b* = 0.06, *p* = 0.60, FDR *p* = 0.73) or any of the 8 subscale scores (*b* range = −0.09–0.07, *p* = 0.14–0.94, FDR *p* = 0.26–0.94) in the cross-ancestry analysis. For EUR-only, associations of SCZ-PRS with CBCL anxious/depressed symptoms (*b* = 0.20, *p* = 0.02, FDR *p* = 0.03) and rule breaking behavior (*b* = 0.20, *p* = 0.001, FDR *p* = 0.003) scores remained significant, whereas associations with total problems and aggressive behavior were no longer significant after accounting for SCZ-FH.

Similar to when modeled alone, SCZ-FH remained significantly associated with PQB Distress scores in the cross-ancestry analysis (*b* = 0.43, *p* < 0.001, FDR *p* < 0.001) and for EUR-only youth (*b* = 0.45, *p* = 0.004, FDR *p* = 0.01), after accounting for SCZ-PRS, while the association did not survive correction for AMR-only youth (*b* = 0.69, *p* = 0.03, FDR *p* = 0.06) and was null in AFR-only youth (*b* = 0.32, *p* = 0.11, FDR *p* = 0.24). Similar to when SCZ-PRS was modeled alone, SCZ-PRS was not associated with PQB Distress scores in cross-ancestry analysis (*b* = 0.15, *p* = 0.18, FDR *p* = 0.31) or within-ancestry models that accounted for SCZ-FH.

In exploratory joint models for clinical diagnoses, SCZ-FH retained most associations with increased odds of disorders including ADHD, depressive, conduct and anxiety disorders in the cross-ancestry analysis. SCZ-PRS remained largely unassociated with diagnoses across ancestries after accounting for SCZ-FH, except for depressive disorders, where the association among AMR-only youth remained significant. See Supplementary Results for details.

### Sensitivity analysis incorporating income-to-needs

Controlling for income-to-needs, most associations with SCZ-FH were consistent with the main analyses, although some associations no longer reached significance. In both independent (Table 3 and Supplementary Figure S2B) and joint models (Table 4 and Figure 2B), SCZ-FH was no longer associated with CBCL attention and rule-breaking symptoms in AFR-only youth, or with somatic and rule-breaking symptoms in AMR-only youth. In the joint model, SCZ-FH was no longer associated with CBCL total and withdrawn symptoms in AMR-only youth. For diagnostic phenotypes, adjusting for income-to-needs in the independent models (Supplementary Table S6 and Supplementary Figure S3B) attenuated associations between SCZ-FH and conduct disorders in AFR-only and AMR-only youth, as well as ADHD diagnoses in AFR-only youth. In contrast, SCZ-PRS associations remained largely unchanged in the joint models (Supplementary Table S8 and Supplementary Figure S4B). These findings suggest that poverty-related factors may partially account for SCZ-FH associations with psychopathology, indicating that SCZ-FH may capture risk beyond genetic predispositions, including environmental influences. The stability of SCZ-PRS associations across models may suggest a more direct genetic contribution and partially distinct pathways of risk.

**Table 3.** Independent associations of schizophrenia polygenic risk scores (SCZ-PRS) and family history of psychosis (SCZ-FH) with dimensional, total cognitive score derived from the NIH-Toolbox, Child Behavior Checklist (CBCL) scores, and prodromal questionnaire (PQB) scores, including income-to-needs as a covariate

Ancestry	Genetic risk measure	Precursor	B	SE	95% CI	Nominal P	FDR P	PVE (%)
All	Family history	NIH Total Cognitive Score	0.10	0.18	−0.26 to 0.46	0.576	0.633	0.00
		CBCL – Total	0.89	0.13	0.65 to 1.14	<0.001	**<0.001**	0.60
		CBCL – Anxious Depressed	0.35	0.07	0.21 to 0.48	<0.001	**<0.001**	0.32
		CBCL – Withdrawn	0.26	0.06	0.13 to 0.39	<0.001	**<0.001**	0.20
		CBCL – Somatic	0.35	0.07	0.21 to 0.48	<0.001	**<0.001**	0.31
		CBCL – Social	0.44	0.05	0.34 to 0.55	<0.001	**<0.001**	0.85
		CBCL – Thought	0.54	0.07	0.40 to 0.67	<0.001	**<0.001**	0.75
		CBCL – Attention	0.41	0.07	0.27 to 0.55	<0.001	**<0.001**	0.40
		CBCL – Rule Breaking	0.24	0.05	0.13 to 0.34	<0.001	**<0.001**	0.22
		CBCL – Aggressive	0.40	0.06	0.27 to 0.52	<0.001	**<0.001**	0.47
		PQB – Distress Score	0.40	0.12	0.17 to 0.63	0.001	**0.001**	0.14
	SCZ PRS	NIH Total Cognitive Score	−0.48	0.18	−0.82 to −0.13	0.007	**0.014**	0.09
		CBCL – Total	0.17	0.12	−0.08 to 0.41	0.178	0.245	0.02
		CBCL – Anxious Depressed	0.10	0.07	−0.03 to 0.23	0.129	0.189	0.03
		CBCL – Withdrawn	0.03	0.06	−0.09 to 0.16	0.616	0.646	0.00
		CBCL – Somatic	−0.09	0.07	−0.22 to 0.05	0.198	0.256	0.02
		CBCL – Social	0.04	0.05	−0.06 to 0.14	0.429	0.525	0.01
		CBCL – Thought	0.10	0.07	−0.03 to 0.23	0.125	0.189	0.03
		CBCL – Attention	−0.01	0.07	−0.14 to 0.13	0.932	0.932	0.00
		CBCL – Rule Breaking	0.12	0.05	0.01 to 0.22	0.029	0.054	0.06
		CBCL – Aggressive	0.04	0.06	−0.08 to 0.16	0.479	0.555	0.01
		PQB – Distress Score	0.20	0.12	−0.02 to 0.43	0.078	0.132	0.04
European	Family History	NIH Total Cognitive Score	−0.09	0.29	−0.65 to 0.47	0.744	0.779	0.00
		CBCL – Total	0.79	0.19	0.42 to 1.16	<0.001	**<0.001**	0.32
		CBCL – Anxious Depressed	0.24	0.11	0.03 to 0.45	0.024	**0.042**	0.10
		CBCL – Withdrawn	0.13	0.10	−0.05 to 0.32	0.163	0.211	0.04
		CBCL – Somatic	0.33	0.11	0.12 to 0.53	0.002	**0.005**	0.18
		CBCL – Social	0.34	0.08	0.18 to 0.49	<0.001	**<0.001**	0.34
		CBCL – Thought	0.61	0.11	0.40 to 0.81	<0.001	**<0.001**	0.62
		CBCL – Attention	0.54	0.11	0.33 to 0.74	<0.001	**<0.001**	0.48
		CBCL – Rule Breaking	0.28	0.08	0.13 to 0.43	<0.001	**<0.001**	0.25
		CBCL – Aggressive	0.36	0.09	0.18 to 0.54	<0.001	**<0.001**	0.28
		PQB – Distress Score	0.44	0.16	0.12 to 0.75	0.007	**0.016**	0.14
	SCZ PRS	NIH Total Cognitive Score	−0.68	0.22	−1.12 to –0.24	0.002	**0.006**	0.18
		CBCL – Total	0.32	0.15	0.03 to 0.61	0.031	**0.049**	0.09
		CBCL – Anxious Depressed	0.20	0.08	0.04 to 0.37	0.017	**0.034**	0.11
		CBCL – Withdrawn	0.10	0.08	−0.05 to 0.24	0.204	0.250	0.03
		CBCL – Somatic	−0.09	0.08	−0.25 to 0.08	0.304	0.352	0.02
		CBCL – Social	0.01	0.06	−0.11 to 0.13	0.839	0.839	0.00
		CBCL – Thought	0.14	0.08	−0.02 to 0.30	0.085	0.116	0.06
		CBCL – Attention	0.04	0.08	−0.12 to 0.21	0.588	0.647	0.01
		CBCL – Rule Breaking	0.21	0.06	0.09 to 0.33	<0.001	**0.002**	0.23
		CBCL – Aggressive	0.16	0.07	0.02 to 0.30	0.025	**0.042**	0.09
		PQB – Distress Score	0.23	0.13	−0.02 to 0.48	0.068	0.100	0.06
African	Family History	NIH Total Cognitive Score	0.13	0.28	−0.42 to 0.68	0.644	0.827	0.01
		CBCL – Total	0.98	0.21	0.56 to 1.40	<0.001	**<0.001**	1.24
		CBCL – Anxious Depressed	0.38	0.10	0.19 to 0.57	<0.001	**<0.001**	0.94
		CBCL – Withdrawn	0.30	0.11	0.09 to 0.51	0.005	**0.016**	0.46
		CBCL – Somatic	0.35	0.11	0.15 to 0.56	0.001	**0.003**	0.65
		CBCL – Social	0.51	0.09	0.33 to 0.68	<0.001	**<0.001**	1.89
		CBCL – Thought	0.50	0.11	0.29 to 0.72	<0.001	**<0.001**	1.22
		CBCL – Attention	0.23	0.12	−0.01 to 0.47	0.064	0.176	0.20
		CBCL – Rule Breaking	0.18	0.11	−0.03 to 0.39	0.101	0.246	0.16
		CBCL – Aggressive	0.38	0.11	0.16 to 0.60	0.001	**0.003**	0.65
		PQB – Distress Score	0.31	0.22	−0.12 to 0.74	0.164	0.328	0.11
	SCZ PRS	NIH Total Cognitive Score	0.19	0.45	−0.69 to 1.07	0.677	0.827	0.01
		CBCL – Total	−0.09	0.36	−0.79 to 0.62	0.812	0.940	0.00
		CBCL – Anxious Depressed	−0.02	0.16	−0.33 to 0.30	0.924	0.988	0.00
		CBCL – Withdrawn	−0.29	0.18	−0.64 to 0.07	0.114	0.250	0.15
		CBCL – Somatic	0.00	0.18	−0.35 to 0.35	0.988	0.988	0.00
		CBCL – Social	0.09	0.15	−0.21 to 0.38	0.571	0.827	0.02
		CBCL – Thought	0.01	0.18	−0.35 to 0.37	0.973	0.988	0.00
		CBCL – Attention	0.09	0.20	−0.30 to 0.49	0.644	0.827	0.01
		CBCL – Rule Breaking	−0.13	0.18	−0.49 to 0.22	0.458	0.725	0.03
		CBCL – Aggressive	−0.17	0.19	−0.54 to 0.21	0.381	0.699	0.05
		PQB – Distress Score	0.28	0.37	−0.46 to 1.01	0.461	0.725	0.03
American Admixed	Family History	NIH Total Cognitive Score	0.53	0.46	−0.36 to 1.43	0.245	0.337	0.11
		CBCL – Total	0.79	0.32	0.16 to 1.43	0.014	**0.044**	0.49
		CBCL – Anxious Depressed	0.49	0.17	0.15 to 0.82	0.004	**0.024**	0.66
		CBCL – Withdrawn	0.44	0.18	0.09 to 0.80	0.014	**0.044**	0.49
		CBCL – Somatic	0.40	0.19	0.04 to 0.77	0.029	0.075	0.39
		CBCL – Social	0.52	0.14	0.24 to 0.79	<0.001	**0.006**	1.08
		CBCL – Thought	0.42	0.17	0.10 to 0.75	0.011	**0.044**	0.52
		CBCL – Attention	0.58	0.18	0.23 to 0.94	0.001	**0.008**	0.86
		CBCL – Rule Breaking	0.24	0.13	−0.01 to 0.50	0.064	0.106	0.28
		CBCL – Aggressive	0.51	0.16	0.20 to 0.82	0.001	**0.008**	0.86
		PQB – Distress Score	0.67	0.33	0.02 to 1.32	0.044	0.084	0.33
	SCZ PRS	NIH Total Cognitive Score	−0.20	0.47	−1.12 to 0.72	0.670	0.737	0.02
		CBCL – Total	0.67	0.33	0.01 to 1.32	0.046	0.084	0.33
		CBCL – Anxious Depressed	0.28	0.18	−0.07 to 0.63	0.119	0.174	0.20
		CBCL – Withdrawn	0.37	0.19	0.01 to 0.74	0.045	0.084	0.33
		CBCL – Somatic	0.12	0.19	−0.26 to 0.49	0.539	0.624	0.03
		CBCL – Social	0.31	0.14	0.03 to 0.60	0.031	0.075	0.38
		CBCL – Thought	0.17	0.17	−0.17 to 0.51	0.337	0.435	0.08
		CBCL – Attention	0.02	0.19	−0.35 to 0.38	0.921	0.921	0.00
		CBCL – Rule Breaking	0.25	0.14	−0.02 to 0.52	0.067	0.106	0.27
		CBCL – Aggressive	0.12	0.16	−0.20 to 0.44	0.451	0.551	0.05
		PQB – Distress Score	0.07	0.34	−0.60 to 0.75	0.829	0.869	0.00

*Note:* FDR P refers to the false discovery rate–corrected *p* value (bold values indicate FDR-corrected p<0.05); PVE refers to the percentage of variance explained.

**Table 4.** Joint model associations of schizophrenia polygenic risk scores (SCZ-PRS) and family history of psychosis (SCZ-FH) with dimensional, total cognitive score derived from the NIH-Toolbox, Child Behavior Checklist (CBCL) scores, and prodromal questionnaire (PQB) scores, including income-to-needs as a covariate

Ancestry	Precursor	Family History of Psychosis (Joint Model)						SCZ PRS (Joint Model)						Joint PVE
		*B*	*SE*	*95% CI*	*Nominal P*	*FDR P*	*PVE*	*B*	*SE*	*95% CI*	*Nominal P*	*FDR P*	*PVE*	
All	NIH Total Cognitive Score	0.11	0.18	−0.25 to 0.48	0.536	0.610	0.00	−0.48	0.18	−0.83 to −0.13	0.007	**0.013**	0.09	0.10
	CBCL – Total	0.89	0.13	0.64 to 1.14	<0.001	**<0.001**	0.60	0.15	0.12	−0.09 to 0.39	0.223	0.289	0.02	0.62
	CBCL – Anxious Depressed	0.34	0.07	0.21 to 0.47	<0.001	**<0.001**	0.31	0.09	0.07	−0.04 to 0.22	0.155	0.227	0.02	0.34
	CBCL – Withdrawn	0.26	0.06	0.13 to 0.39	<0.001	**<0.001**	0.19	0.03	0.06	−0.10 to 0.15	0.672	0.704	0.00	0.20
	CBCL – Somatic	0.35	0.07	0.22 to 0.48	<0.001	**<0.001**	0.31	−0.09	0.07	−0.23 to 0.04	0.165	0.227	0.02	0.33
	CBCL – Social	0.44	0.05	0.34 to 0.55	<0.001	**<0.001**	0.85	0.03	0.05	−0.07 to 0.13	0.528	0.610	0.00	0.86
	CBCL – Thought	0.54	0.07	0.40 to 0.67	<0.001	**<0.001**	0.74	0.09	0.07	−0.04 to 0.22	0.165	0.227	0.02	0.77
	CBCL – Attention	0.41	0.07	0.27 to 0.55	<0.001	**<0.001**	0.40	−0.01	0.07	−0.15 to 0.12	0.846	0.846	0.00	0.40
	CBCL – Rule Breaking	0.23	0.05	0.13 to 0.34	<0.001	**<0.001**	0.22	0.11	0.05	0.01 to 0.22	0.036	0.066	0.05	0.28
	CBCL – Aggressive	0.39	0.06	0.27 to 0.52	<0.001	**<0.001**	0.47	0.04	0.06	−0.08 to 0.16	0.555	0.610	0.00	0.48
	PQB – Distress Score	0.40	0.12	0.17 to 0.63	<0.001	**0.001**	0.14	0.20	0.12	−0.03 to 0.42	0.090	0.152	0.03	0.18
European	NIH Total Cognitive Score	−0.06	0.29	−0.62 to 0.50	0.828	0.867	0.00	−0.68	0.22	−1.12 to −0.24	0.002	**0.006**	0.18	0.18
	CBCL – Total	0.78	0.19	0.41 to 1.15	<0.001	**<0.001**	0.31	0.30	0.15	0.01 to 0.59	0.045	0.070	0.08	0.40
	CBCL – Anxious Depressed	0.23	0.11	0.02 to 0.44	0.030	0.055	0.09	0.19	0.08	0.03 to 0.36	0.021	**0.043**	0.10	0.19
	CBCL – Withdrawn	0.13	0.10	−0.06 to 0.32	0.178	0.230	0.03	0.09	0.08	−0.06 to 0.24	0.224	0.273	0.03	0.06
	CBCL – Somatic	0.33	0.11	0.12 to 0.54	0.002	**0.005**	0.18	−0.10	0.08	−0.26 to 0.07	0.251	0.291	0.02	0.20
	CBCL – Social	0.34	0.08	0.18 to 0.49	<0.001	**<0.001**	0.34	0.00	0.06	−0.12 to 0.12	0.967	0.967	0.00	0.34
	CBCL – Thought	0.60	0.11	0.39 to 0.81	<0.001	**<0.001**	0.61	0.12	0.08	−0.04 to 0.29	0.130	0.179	0.04	0.66
	CBCL – Attention	0.53	0.11	0.33 to 0.74	<0.001	**<0.001**	0.47	0.03	0.08	−0.13 to 0.19	0.724	0.796	0.00	0.48
	CBCL – Rule Breaking	0.27	0.08	0.12 to 0.42	<0.001	**0.002**	0.23	0.20	0.06	0.08 to 0.32	<0.001	**0.003**	0.21	0.46
	CBCL – Aggressive	0.35	0.09	0.17 to 0.53	<0.001	**<0.001**	0.27	0.15	0.07	0.01 to 0.29	0.035	0.060	0.08	0.36
	PQB – Distress Score	0.42	0.16	0.11 to 0.74	0.009	**0.019**	0.13	0.22	0.13	−0.03 to 0.47	0.085	0.125	0.06	0.19
African	NIH Total Cognitive Score	0.13	0.28	−0.42 to 0.67	0.653	0.840	0.01	0.18	0.45	−0.70 to 1.06	0.687	0.840	0.01	0.02
	CBCL – Total	0.98	0.21	0.56 to 1.40	<0.001	**<0.001**	1.24	−0.12	0.36	−0.81 to 0.58	0.742	0.859	0.01	1.24
	CBCL – Anxious Depressed	0.38	0.10	0.19 to 0.57	<0.001	**<0.001**	0.94	−0.03	0.16	−0.34 to 0.29	0.865	0.952	0.00	0.94
	CBCL – Withdrawn	0.30	0.11	0.09 to 0.51	0.005	**0.014**	0.47	−0.30	0.18	−0.65 to 0.06	0.102	0.224	0.16	0.62
	CBCL – Somatic	0.35	0.11	0.15 to 0.56	<0.001	**0.003**	0.65	−0.01	0.18	−0.36 to 0.33	0.936	0.957	0.00	0.65
	CBCL – Social	0.51	0.09	0.33 to 0.68	<0.001	**<0.001**	1.89	0.07	0.15	−0.22 to 0.36	0.645	0.840	0.01	1.91
	CBCL – Thought	0.50	0.11	0.29 to 0.72	<0.001	**<0.001**	1.22	−0.01	0.18	−0.37 to 0.35	0.957	0.957	0.00	1.22
	CBCL – Attention	0.23	0.12	−0.01 to 0.46	0.065	0.180	0.20	0.09	0.20	−0.31 to 0.48	0.670	0.840	0.01	0.21
	CBCL – Rule Breaking	0.18	0.11	−0.03 to 0.39	0.098	0.224	0.16	−0.14	0.18	−0.49 to 0.21	0.439	0.742	0.04	0.19
	CBCL – Aggressive	0.38	0.11	0.16 to 0.60	<0.001	**0.003**	0.66	−0.18	0.19	−0.55 to 0.19	0.345	0.632	0.05	0.70
	PQB – Distress Score	0.30	0.22	−0.13 to 0.73	0.168	0.336	0.11	0.27	0.37	−0.47 to 1.00	0.476	0.748	0.03	0.14
American Admixed	NIH Total Cognitive Score	0.54	0.46	−0.36 to 1.43	0.241	0.331	0.12	−0.21	0.47	−1.14 to 0.71	0.650	0.715	0.02	0.13
	CBCL – Total	0.78	0.32	0.14 to 1.41	0.016	0.050	0.47	0.64	0.33	−0.01 to 1.30	0.053	0.098	0.30	0.80
	CBCL – Anxious Depressed	0.48	0.17	0.14 to 0.82	0.005	**0.028**	0.64	0.26	0.18	−0.08 to 0.61	0.137	0.200	0.18	0.84
	CBCL – Withdrawn	0.43	0.18	0.08 to 0.79	0.016	0.050	0.47	0.36	0.19	−0.00 to 0.73	0.052	0.098	0.31	0.80
	CBCL – Somatic	0.40	0.19	0.04 to 0.77	0.031	0.084	0.38	0.11	0.19	−0.27 to 0.48	0.579	0.670	0.03	0.41
	CBCL – Social	0.51	0.14	0.23 to 0.78	<0.001	**0.007**	1.05	0.30	0.14	0.01 to 0.58	0.039	0.096	0.34	1.43
	CBCL – Thought	0.42	0.17	0.09 to 0.75	0.012	0.050	0.51	0.15	0.17	−0.18 to 0.49	0.373	0.482	0.06	0.59
	CBCL – Attention	0.58	0.18	0.23 to 0.94	0.001	**0.009**	0.86	0.00	0.18	−0.36 to 0.36	0.994	0.994	0.00	0.86
	CBCL – Rule Breaking	0.24	0.13	−0.02 to 0.49	0.071	0.118	0.27	0.24	0.14	−0.02 to 0.51	0.075	0.118	0.26	0.54
	CBCL – Aggressive	0.51	0.16	0.20 to 0.81	0.001	**0.009**	0.85	0.11	0.16	−0.21 to 0.43	0.501	0.612	0.04	0.90
	PQB – Distress Score	0.67	0.33	0.02 to 1.32	0.045	0.098	0.33	0.06	0.34	−0.62 to 0.73	0.871	0.913	0.00	0.33

*Note:* FDR P refers to the false discovery rate–corrected *p* value (bold values indicate FDR-corrected p<0.05); PVE refers to the percentage of variance explained.

Exploratory analyses using family conflict or parental monitoring as covariates showed minimal attenuation of associations between SCZ-FH and psychopathology (see Supplementary Results). This suggests that the attenuated SCZ-FH associations observed when controlling for income-to-needs are not explained by family conflict or parental monitoring.

## Discussion

This study demonstrates partially distinct and independent associations of SCZ-FH and SCZ-PRS with cognitive, behavioral, and emotional functioning in childhood in the ABCD study. SCZ-FH showed broad cross-ancestry associations with dimensional and diagnostic markers of psychopathology, with minimal associations to cognitive functioning. In contrast, SCZ-PRS showed more specific associations to lower cognitive functioning across ancestries, and total, anxious-depressed, rule-breaking, and aggressive behavioral symptoms for the EUR-like ancestry group. Notably, when both SCZ-FH and SCZ-PRS were included in joint models, SCZ-FH retained robust associations across psychopathology measures, while SCZ-PRS effects on cognition and specific behavioral and emotional symptoms persisted, underscoring their relatively independent associations with functioning.

Across ancestries, greater SCZ-FH was linked with greater emotional and behavioral problems, more distressing childhood psychotic-like experiences, and a higher likelihood of lifetime psychiatric diagnoses, including ADHD, depressive, conduct, and anxiety disorders. These findings align with previous ABCD-specific findings, which showed that a binary measure of SCZ-FH was associated with internalizing and externalizing problems, psychotic-like symptoms, and KSADS reports of ADHD and PTSD among EUR-like ancestry (Loughnan et al., 2022). Interestingly, that study identified associations between SCZ-FH and KSADS-based depressive and conduct disorders in EUR-like ancestry youth after controlling for socioeconomic status (SES), which we did not observe in our analysis. Conversely, we found associations between SCZ-FH and anxiety disorders, a relationship not reported by Loughnan et al. (2022). This incongruence likely reflects differences in the operationalization of SCZ-FH and SES. Both studies used the same question to assess SCZ-FH, but we employed a weighted measure accounting for the number of affected relatives and degree of relatedness. Similarly, we defined SES using the income-to-needs ratio, whereas the prior study used parental education and household income. Nevertheless, current results highlight robust associations between greater SCZ-FH and emotional and behavioral problems in childhood and suggest that a weighted SCZ-FH measure may reveal associations with some diagnoses not observed previously.

Associations between SCZ-PRS and childhood psychopathology varied across ancestries. For EUR-like ancestry youth, higher SCZ-PRS was linked to increased CBCL total problems, anxious/depressed, rule-breaking, and aggressive behavior symptoms. For AMR-like ancestry youth, SCZ-PRS was associated with depressive diagnoses. Although SCZ-PRS was associated with some CBCL variables in the EUR-only, these links were weaker than those observed for SCZ-FH. Nevertheless, we observed more associations with emotional and behavioral problems at baseline than reported in some recent studies. For example, Wainberg et al. (2022) did not find significant associations between SCZ-PRS and the eight CBCL subscales in unrelated European ancestry youth, while Loughnan et al. (2022) noted a relationship only with CBCL Rule-Breaking. This may be due to our use of a GRM to account for relatedness, enabling a larger sample than studies that excluded related individuals. Furthermore, differences in PRS generation methods, with our study employing PRS-CSx, may have facilitated our ability to detect these associations. Overall, SCZ-PRS showed weak but detectable associations with childhood psychopathology, suggesting modest effects of molecularly defined genetic risk for SCZ on childhood psychopathology.

Cognitive functioning in childhood was not associated with SCZ-FH using our primary weighted count-based measure, but interestingly, did show an association in EUR-only youth for the alternative SCZ-FH proportion version of the measure, which accounted for the number of first- and second-degree relatives, but was not associated with SCZ-PRS. In contrast, SCZ-PRS was associated with poorer cognition in the cross-ancestry and EUR-only analyses, consistent with prior research (Loughnan et al., 2022; Mollon & Reichenberg, 2018). Offspring of parents with psychosis have been observed to exhibit cognitive deficits (MacKenzie et al., 2020; Seidman, 2010). Weaker associations of SCZ-FH with cognition in ABCD may reflect the sample’s normative characteristics, reliance on a single-question SCZ-FH measure, and/or the use of NIH-TB over traditional neurocognitive assessments like the Wechsler Intelligence Scale for Children (WISC). Nevertheless, our results suggest that poorer cognitive function may more directly reflect genetic risk for SCZ than broader aspects of SCZ risk that are captured by SCZ-FH.

The effect sizes and significance levels for SCZ risk predictors in the joint models closely mirrored those in the independent models, suggesting that SCZ-FH and SCZ-PRS capture partially distinct aspects of SCZ risk. This aligns with growing evidence that both offer independent yet complementary insights into disease susceptibility for SCZ and other conditions (Loughnan et al., 2022; Lu et al., 2018; Mars et al., 2022). SCZ-FH may capture environmental aspects of risk, such as intergenerational exposure to stress, or increased symptom recognition in families with a history of SCZ (Bowers & Yehuda, 2016; Oliver-Parra, Dalmau-Bueno, Ruiz-Muñoz, & García-Altés, 2020). Consistent with this, some associations between SCZ-FH and psychopathology symptoms were attenuated after accounting for income-to-needs, whereas associations between SCZ-PRS and cognitive functioning, as well as a subset of psychopathology symptoms in European ancestry youth, were unchanged. Interestingly, exploratory analysis including family conflict and parental monitoring did not show similar attenuation, suggesting that income-to-needs may reflect broader contextual factors shaping associations between SCZ risk and child outcomes. Together, these findings highlight the potential clinical value of combining SCZ-FH and SCZ-PRS for early risk assessment.

Although we identified associations between SCZ-PRS and aspects of cognitive and emotional functioning in EUR-like ancestry youth, associations in non-European ancestry groups were weaker. While we leveraged discovery GWAS with greater ancestral diversity, disparities in predictive accuracy across ancestry groups persist, likely due to residual imbalances in GWAS power and sample composition. These findings underscore the importance of improving PRS accuracy through increased GWAS representation, continued PRS methods development, and clearer analytic standards. For example, our use of within-ancestry z-scoring aligns with a common scaling method for creating PRS clinical cutoffs or odds ratios across groups, though no definitive standard yet exists (Khan et al., 2022). Emerging approaches for accounting for ancestry differences, such as using covariates reflecting genetic distance to original GWAS samples, are promising, but methods for implementation are still developing (Ding et al., 2023). In summary, while PRS methodology and inclusion efforts have advanced, further work is needed to establish robust, inclusive standards for cross-ancestry PRS analyses to better understand genetically mediated precursors for disorders.

### Limitations

One limitation is the reliance on parent-report CBCL and KSADS-COMP measures, which, while generally showing stronger concurrent validity with gold-standard clinician interviews for diagnoses and symptom severity at this age (Townsend et al., 2020; Warnick, Bracken, & Kasl, 2008), may introduce reporting biases (Robinson et al., 2019). Second, psychometric properties of the cognitive functioning measure derived from NIH-TB are less robust than some traditional measures, such as the WISC (Taylor et al., 2022). However, the NIH-TB has notable advantages, including ease of administration, which improves scalability, making it a reasonably effective measure of cognition (Distefano et al., 2023). Additionally, although the ABCD study aimed to reflect general U.S. demographics, a general population sample may be lower powered to detect associations between SCZ risk and aspects of functioning because of the low base rate of SCZ-relevant markers compared to samples enriched for SCZ risk. Lastly, our SCZ-FH variable was derived from a single FHAM-S item assessing psychotic symptoms in relatives, which may not capture the full complexity of family history. However, we aimed to improve its informativeness by modeling it continuously rather than dichotomously. Our finding that the weighted count version of the SCZ-FH variable used in primary analyses was associated with SCZ-PRS in EUR ancestry youth supports its convergent validity as a measure of psychosis family history.

## Conclusion

In summary, the current study found robust associations between SCZ-FH and broad psychopathology, with weaker, inconsistent associations with cognitive functioning in the ABCD Study. Conversely, SCZ-PRS demonstrated stronger associations with cognition across ancestries and with specific emotional and behavioral symptoms in EUR-like ancestry youth. These patterns persisted when SCZ-FH and SCZ-PRS were jointly modeled, highlighting the complementary insights provided by both measures. Nevertheless, associations between SCZ-PRS and cognitive and emotional functioning in non-European ancestry youth were substantially weaker compared to EUR-like ancestry youth. Clarifying whether this reflects lower SCZ-PRS accuracy or greater influence of other risk factors on childhood functioning in diverse populations is crucial. Future research should prioritize inclusive methodologies to improve understanding of SCZ risk across diverse youth, and longitudinal studies to explore the temporal progression of these relationships.

## Supporting information

Hyat et al. supplementary material Hyat et al. supplementary material

## Supplementary material

The supplementary material for this article can be http://doi.org/10.1017/S0033291725102304.
