# Supplementary material for: Independent versus joint effects of polygenic or family-based schizophrenia risk in diverse ancestry youth in the ABCD study
Source: Psychol Med. 2025 Oct 30;55:e327. doi: 10.1017/S0033291725102304 (PMC13054910; doi:10.1017/S0033291725102304)
Supplement: Hyat et al. supplementary material [file S0033291725102304sup001.docx]

**Supplementary Information**

**Supplementary Methods**

Measures

*SCZ Risk*

SCZ-FH was derived from the version of the Family History Assessment Module Screener (FHAM-S) administered in the ABCD Study, which obtains reports on the presence or absence of symptoms associated with various conditions including alcohol use disorder, depression, mania and psychosis in all 1st and 2nd degree “blood relatives” of the youth (Barch et al., 2018). The FHAM was originally developed for the Collaborative Study on the Genetics of Alcoholism (COGA) and demonstrated strong reliability when compared with diagnoses obtained through direct interviews (Rice et al., 2016). Since then, it has been widely adopted by studies as a primary method for assessing family history of substance use and psychiatric disorders, such as in the National Consortium on Alcohol and Neurodevelopment in Adolescence (NCANDA) study (Brown et al., 2015). While to the best of our knowledge, no published studies have specifically evaluated the psychometric performance of the FHAM-S psychosis variable, our finding that the weighted count version of the SCZ-FH variable used for primary analyses in the current study was associated with SCZ-PRS in EUR ancestry youth provides evidence for convergent validity for this measure for psychosis family history.

*SCZ-related Signs and Symptoms*

I) Dimensional Assessments

A. NIH Cognitive Toolbox

Children completed tasks from the NIH Cognitive Toolbox (NIH-TB) which quantifies cognitive functioning across various domains (Gershon et al., 2013; Weintraub et al., 2013). The tasks include Flanker (inhibitory control), List Sorting (working memory), Picture Sequence (episodic memory), Oral Reading Recognition (language), Picture Vocabulary (language), and Pattern Comparison (processing speed) subtests. The Flanker task tests participants’ ability to inhibit attention from irrelevant stimuli. List Sorting requires the participant to recall and sequence different stimuli while Picture Sequence is a test of episodic memory and involves the reproduction of pictures. Both Oral Reading Recognition and Picture Vocabulary assess language skills with the former requiring participants to read letters and words aloud, while the latter involves selecting the most fitting picture for a word presented audibly. Lastly, Pattern Comparison measures processing speed by requiring participants to quickly determine whether two stimuli are the same. A total composite, age-corrected cognitive score, summarizing functioning across these tasks was used for analysis, after winsorizing scores to account for outliers beyond three standard deviations.

B. Child Behavior Checklist

Primary caregivers completed the CBCL which is a well-validated and widely used assessment involving 113 questions to measure emotional and behavioral problems in children over the past 6 months (Achenbach, 1991). The total composite score, along with scores for the eight primary subscales: anxious/depressed, withdrawn/depressed, somatic complaints, social problems, thought problems, attention problems, rule-breaking behavior, and aggressive behavior severity were used for analysis.

C. Prodromal Questionnaire – Brief Child Version

Children completed the Prodromal Questionnaire – Brief Child Version (PQ-BC) which is a 21-item scale that assesses the occurrences of psychotic-like experiences (e.g. perceptual abnormalities), and level of distress for any endorsed experience. We generated a summary PQ-BC Distress score by the adding the total number of endorsements weighted by distress (for each item, 0 = did not experience PLE, 1 = experienced PLE with no distress, 2-6 = distressing PLE [+1 to the distress score for the item]) (Chang et al., 2024). The PQ-BC Distress score was used as the measure of psychotic-like experiences (PLEs) for analysis.

II) Diagnostic Assessments

A. Kiddie Schedule for Affective Disorders and Schizophrenia

Caregivers and youth completed a semi-structured, self-administered, computerized version of the KSADS-5 which assesses mental health conditions experienced by the child, including depression, bipolar disorder, anxiety disorders, psychosis, conduct disorder and ADHD (KSADS-COMP; Kaufman et al., 1997). Lifetime history of depressive disorders, conduct disorder, anxiety disorders (with and without post-traumatic stress disorder) and attention-deficit/hyperactivity disorder (ADHD) diagnoses were derived from the caregiver self-administered KSADS-COMP, as they have been shown to have greater concordance with diagnoses derived from gold-standard clinician interviews integrating parent and youth report, compared to diagnoses derived from the youth self-administered KSADS-COMP (Townsend et al., 2020). For the diagnosis of depressive disorders, lifetime disruptive mood dysregulation disorder, major depressive disorder, or persistent depressive disorder, were included. For the diagnosis of anxiety disorders, agoraphobia, generalized anxiety disorder, panic, separation anxiety, and social anxiety disorder, were included. An additional metric for lifetime history of anxiety disorders including post-traumatic stress disorder (PTSD), was also analyzed. To facilitate comparison with dimensional psychopathology measures derived from the CBCL, analyses of KSADS-derived clinical diagnoses utilized lifetime history of any depressive disorder, any anxiety disorder (including and excluding PTSD), conduct disorder, and ADHD. Potential issues with KSADS ADHD diagnoses in ABCD have been noted previously due to a modification of the diagnostic criterion to require impairment in two or more settings, which yielded an unexpectedly low endorsement rate (Barch et al., 2021). To address this, we re-coded the variable to include cases with impairment in just one setting, an approach that aligns with the criterion originally used in Data Release 3.

B. CBCL ADHD variable

To facilitate comparison with alternate methods for deriving ADHD diagnoses, we generated an additional ADHD variable using CBCL cut-offs. A T-score of ≥65 on the CBCL Attention Problems scale was established as the threshold for clinical-level attention problems. A key distinction between the diagnostic measures is that the KSADS variables reflect the lifetime prevalence of ADHD, while the CBCL-derived ADHD measure captures symptoms occurring only within the past six months.

*Family Conflict and Parental Monitoring*

We generated a family conflict variable using data from the baseline youth-reported conflict subset of the Family Environment Scale and by averaging responses across nine questions, in line with prior studies (Al‐shoaibi et al., 2024). Missing responses were ignored in the calculation, such that participants with one or more available responses still received a score (i.e., the mean was computed based on the number of non-missing items). Internal consistency for the scale was acceptable (Cronbach’s α = 0.68). We also created a variable to capture family monitoring by utilizing baseline data from the Parental Monitoring Scale which assessed parents’ monitoring and knowledge of their children’s whereabouts and who their children were spending time with (Trevino et al., 2024). Scores were created by averaging responses across five items, and missing responses were dealt in the same manner as for the calculation of the family conflict variable.

Statistical Analysis

*Ancestry Principal Components, Ancestry Grouping, and Genetic Relatedness*

To create ancestry groups of subjects, we conducted principal components analysis (PCA) using high-quality SNPs from the ABCD dataset merged with HapMap3 data as the reference (The International HapMap 3 Consortium, 2010). HapMap3 includes genotype data from 11 global populations, providing a robust reference for ancestry analysis. To ensure inclusion of only high-quality SNPs, we excluded variants that violated Hardy-Weinberg Equilibrium at a p-threshold of 0.001 during the merging process (Chang et al., 2024). Ancestry representative PCs were generated following an iterative procedure optimized for samples from diverse ancestral groups with familial relatedness (Conomos, Laurie, et al., 2016). This involved using PC-AiR for PC calculation and PC-Relate for kinship coefficient estimation, implemented in the GENESIS R package (Conomos, Miller, & Thornton, 2015; Conomos, Reiner, Weir, & Thornton, 2016).

We utilized these ancestry PCs as inputs for a Random Forest classifier to categorize participants into ancestry groups based on genetic similarity, using HapMap3 super-population labels (Alexander & Lange, 2011). The top eight PCs, which captured the majority of ancestry variance, were used to train the classifier. A probability threshold of 0.7 was applied for ancestry group assignment, leading to a slight reduction in the sample size from 10,979 to 9,365 participants but better grouping accuracy. Figure S1 provides a visualization of the variance explained by the top 8 PCs.

*Association Testing*

To optimize computational efficiency, the GRMs were made sparse by setting a fourth-degree relatedness threshold; values in the GRM below this threshold were set to 0. We utilized the fitNullModel function within the GENESIS package to perform linear and logistic regressions, as it accommodates matrix covariates (e.g. the GRM) as random effects. P-values for all dimensional measures were corrected for multiple testing using false discovery rate (FDR) correction within each ancestry group, across both risk SCZ measures. P-values for all diagnostic outcomes were similarly FDR-corrected for multiple testing within each ancestry group, across both risk SCZ measures. We also conducted a sensitivity analysis incorporating the income-to-needs ratio as an additional covariate. This ratio was calculated by dividing the median income value within each income band by the federal poverty threshold, adjusted for household size (Hair et al., 2015). Exploratory analyses alternatively incorporated measures of family conflict or parental monitoring as covariates.

**Supplementary Results**

Independent associations between SCZ Risk Measures & Diagnoses in Childhood

SCZ-FH was significantly associated with lifetime history of multiple diagnoses. In the cross-ancestry analysis, SCZ-FH was associated with higher likelihood of a depressive (*OR*=1.14 p<0.001, FDR p<0.001), conduct (*OR*=1.10, p<0.001, FDR p<0.001), and anxiety disorder, excluding (*OR*=1.21, p<0.001, FDR p<0.001), or including PTSD (*OR*=1.22, p<0.001, FDR p<0.001), as well as ADHD (*OR*=1.13, p<0.001, FDR p<0.001). CBCL-based diagnosis of ADHD was also associated with SCZ-FH (*OR*=1.20, p<0.001, FDR p<0.001). Associations within specific ancestries were similar, including showing significant associations across diagnoses in AFR-only analyses, although associations for depressive and conduct disorders were not significant in EUR-only analyses (Figure S3A, Table S5). The associations for depressive and conduct disorder were also not significant in the AMR-only analyses. SCZ-PRS was not significantly associated with lifetime history of the assessed diagnoses in the cross-ancestry analysis or within individual ancestry groups for most disorders. However, there was a significant association between SCZ-PRS and depressive disorders among AMR-only youth (*OR*=1.37, p=0.008, FDR p=0.02).

Joint associations between SCZ Risk Measures & Diagnostic Measures

In the cross-ancestry analysis, greater SCZ-FH remained associated with higher likelihood of a depressive disorder (*OR*=1.14, p<0.001, FDR p<0.001), conduct disorder (*OR*=1.10, p<0.001, FDR p<0.001), anxiety disorder excluding (*OR*=1.21, p<0.001, FDR p<0.001), or including PTSD (*OR*=1.22, p<0.001, FDR p<0.001), and ADHD (*OR*=1.13, P<0.001, FDR p<0.001). Results were similar using CBCL-based current ADHD (*OR*=1.20, p<0.001, FDR p<0.001). As in the independent model, associations of SCZ-FH with depressive and conduct disorder did not survive correction for multiple testing for EUR-only or AMR-only youth (Figure S4A, Table S7). SCZ-PRS was also not associated with history of any diagnoses in the cross-ancestry analysis or within specific ancestries after accounting for SCZ-FH, except for depressive disorders, for which the association with SCZ-PRS among AMR-only youth remained significant (*OR*=1.37, p=0.01, FDR p=0.02).

**Supplementary Tables & Figures**

Figure S1. Scatterplot of the top 8 ancestry PCs, including the ABCD and HapMap3 reference samples. CEU: Utah residents with Northern and Western European ancestry; CHB: Han Chinese in Beijing, China; YRI: Yoruba in Ibadan, Nigeria; TSI: Toscans in Italy; JPT: Japanese in Tokyo, Japan; CHD: Chinese in Metropolitan Denver, Colorado; MEX: Mexican ancestry in Los Angeles, California; GIH: Gujarati Indians in Houston, Texas; ASW: Gujarati Indians in Houston, Texas; LWK: Luhya in Webuye, Kenya; MKK: Maasai in Kinyawa, Kenya

Figure S2. Heatmap of independent associations of schizophrenia polygenic risk scores (SCZ-PRS) and family history of psychosis (SCZ-FH) with total cognitive score derived from the NIH-Toolbox, Child Behavior Checklist (CBCL) scores, and prodromal questionnaire (PQB) scores A) without income-to-needs ratio as a covariate and (B) with income-to-needs ratio as a covariate. FDR P refers to the false discovery rate–corrected p value.

1. Without income-to-needs ratio


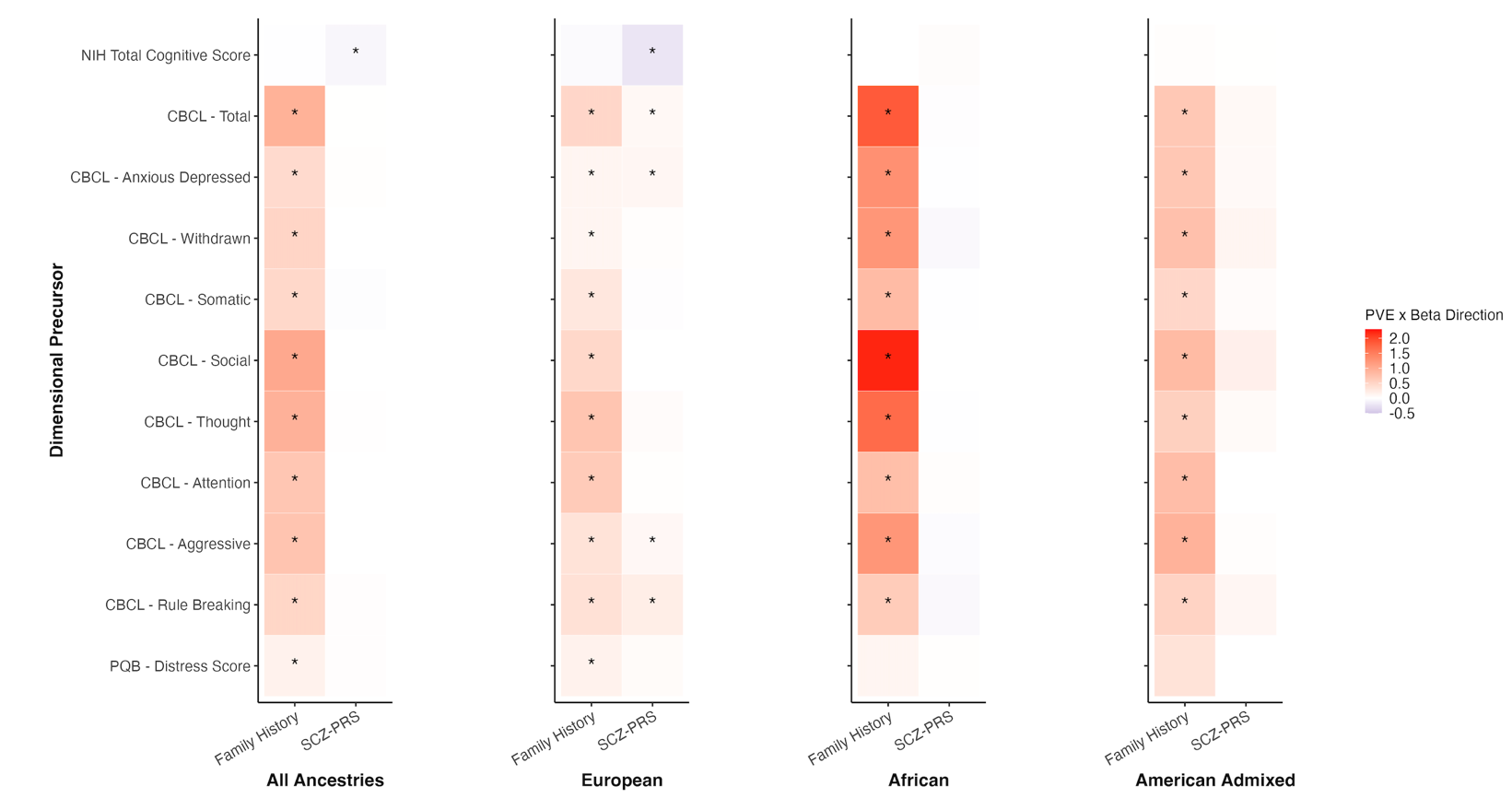


* FDR P < 0.05

1. With income-to-needs ratio


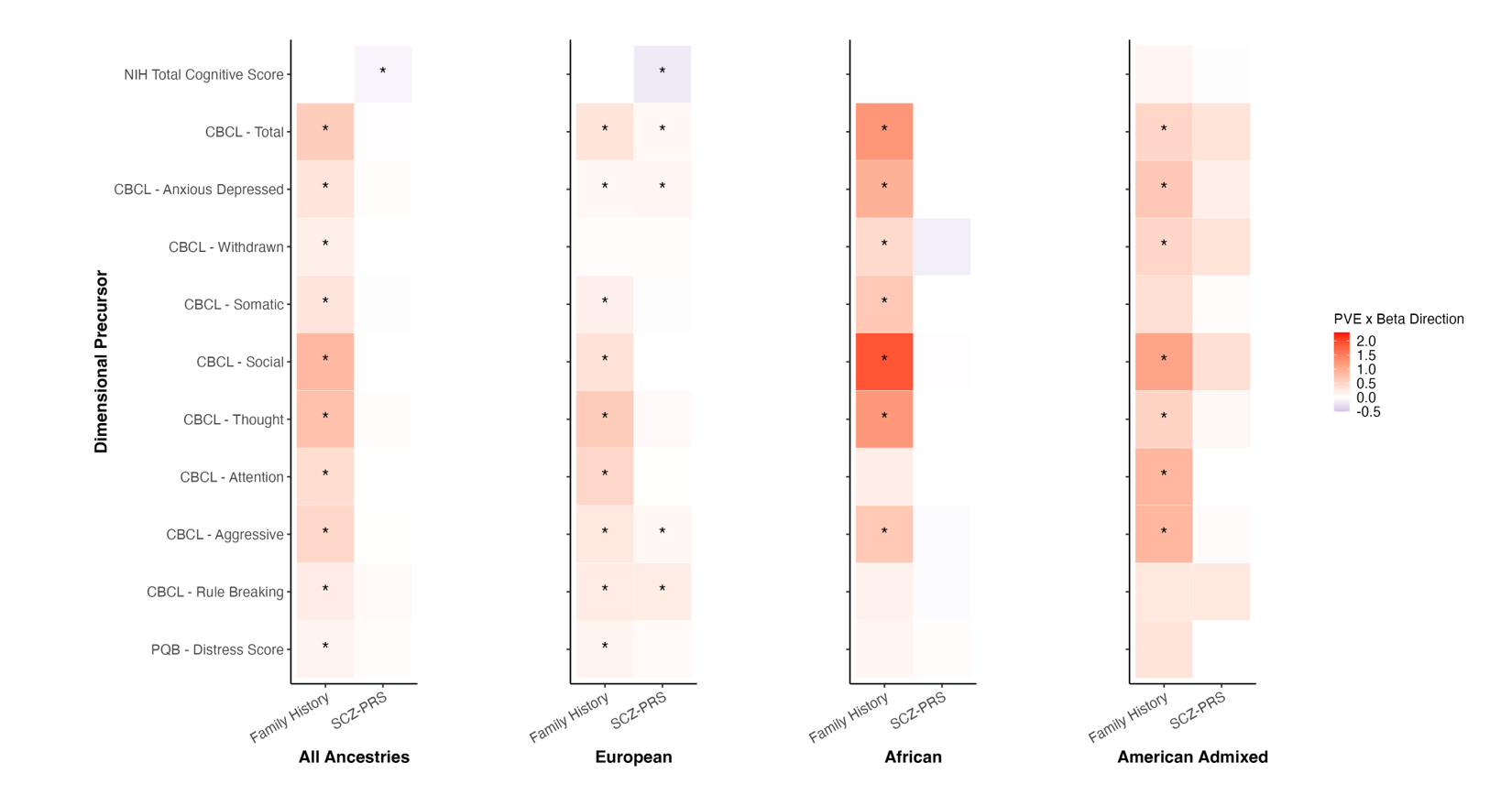


* FDR P < 0.05

Figure S3. Heatmap of independent associations of schizophrenia polygenic risk scores (SCZ-PRS) and family history of psychosis (SCZ-FH) with lifetime history of psychiatric diagnoses derived from the Kiddie Schedule for Affective Disorders and Schizophrenia (KSADS) (A) without income-to-needs ratio as a covariate and (B) with income-to-needs ratio as a covariate. FDR P refers to the false discovery rate–corrected p value.

1. Without income-to-needs ratio


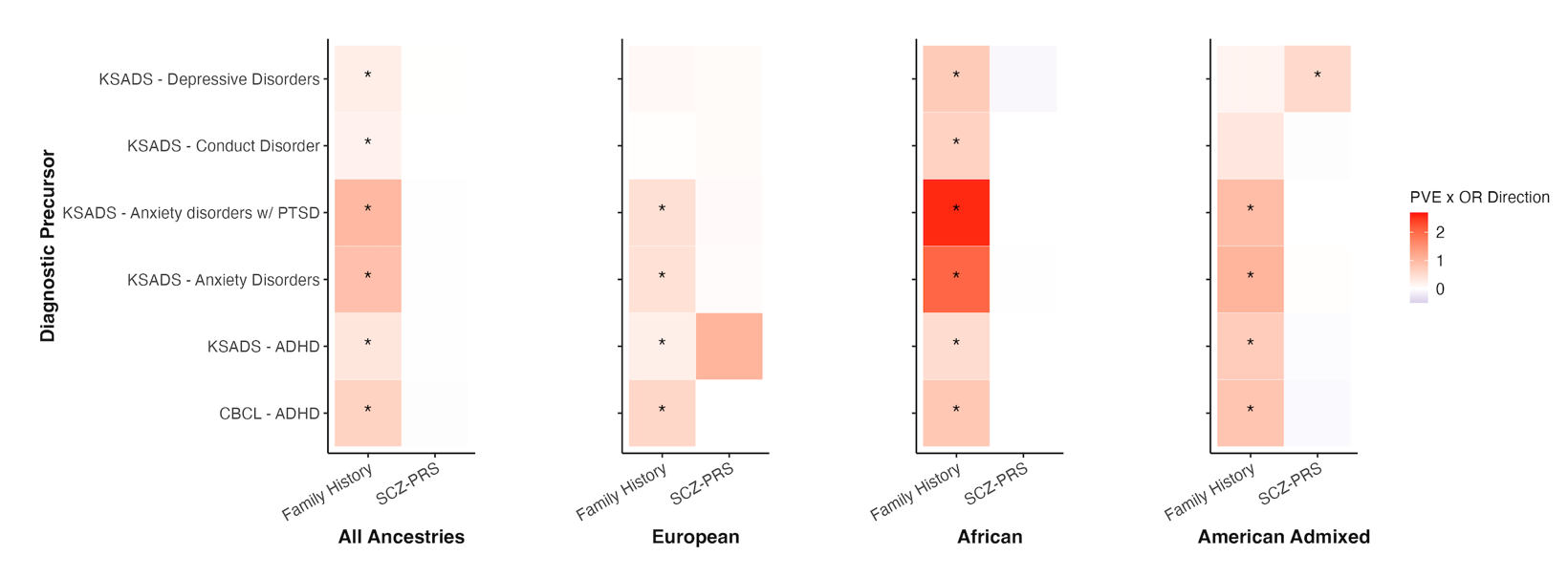


* FDR P < 0.05

1. With income-to-needs ratio


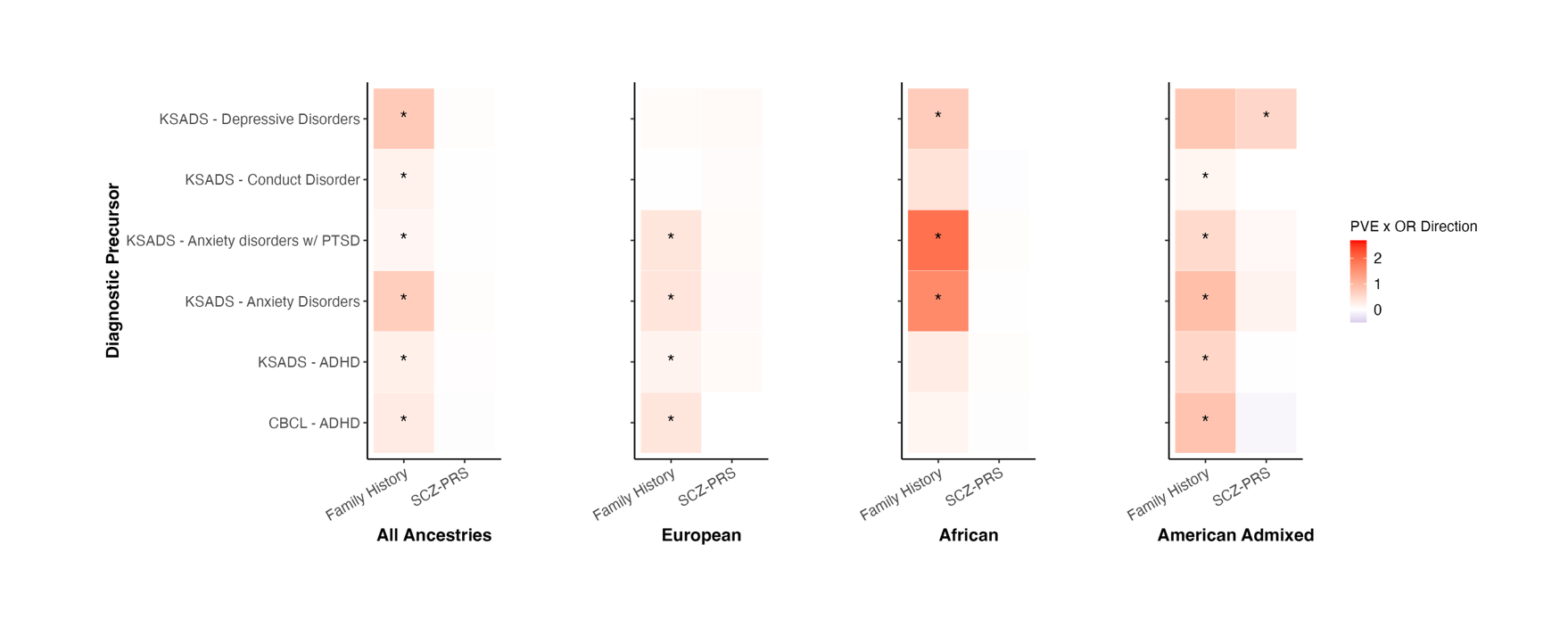


* FDR P < 0.05

Figure S4. Heatmap of joint associations of schizophrenia polygenic risk scores (SCZ-PRS) and family history of psychosis (SCZ-FH) with lifetime history of psychiatric diagnoses derived from the Kiddie Schedule for Affective Disorders and Schizophrenia (KSADS) (A) without income-to-needs ratio as a covariate and (B) with income-to-needs ratio as a covariate. Heatmap shows associations of one genetic risk measure (i.e. SCZ-PRS or SCZ-FH) while adjusting for the other. FDR P refers to the false discovery rate–corrected p value.

1. Diagnostic phenotypes without income-to-needs ratio as a covariate


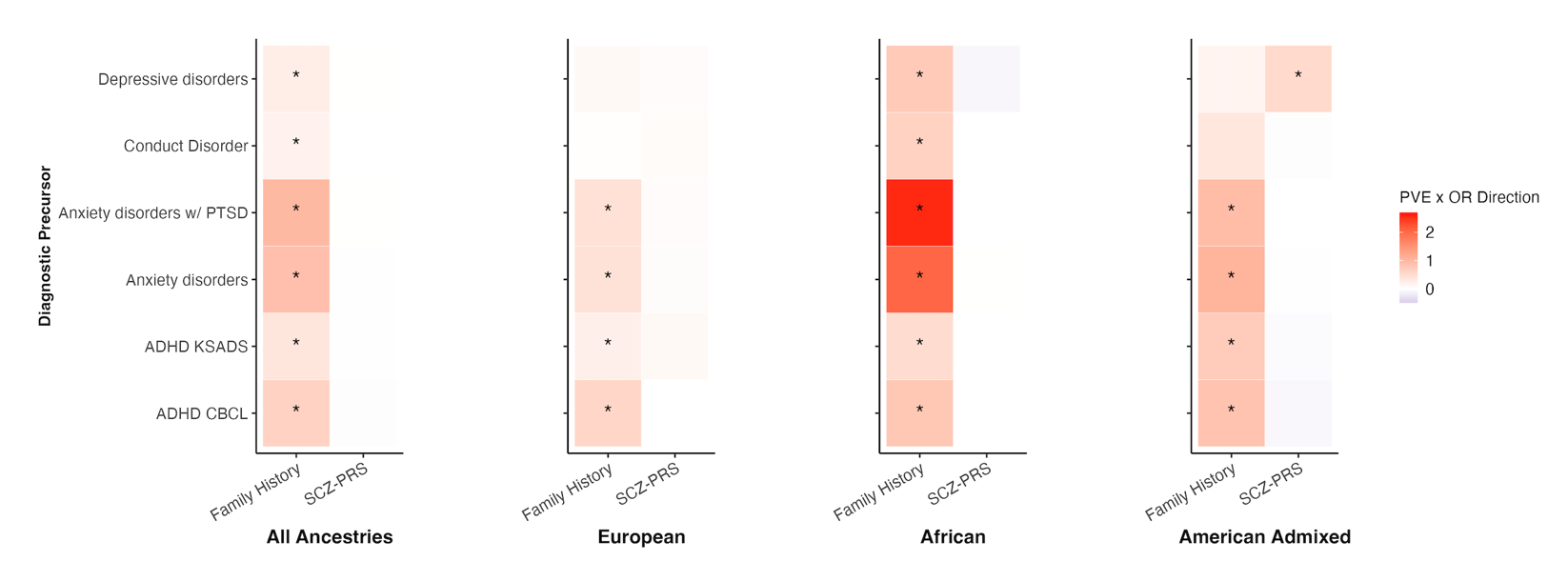


* FDR P < 0.05

1. Diagnostic phenotypes with income-to-needs ratio as a covariate


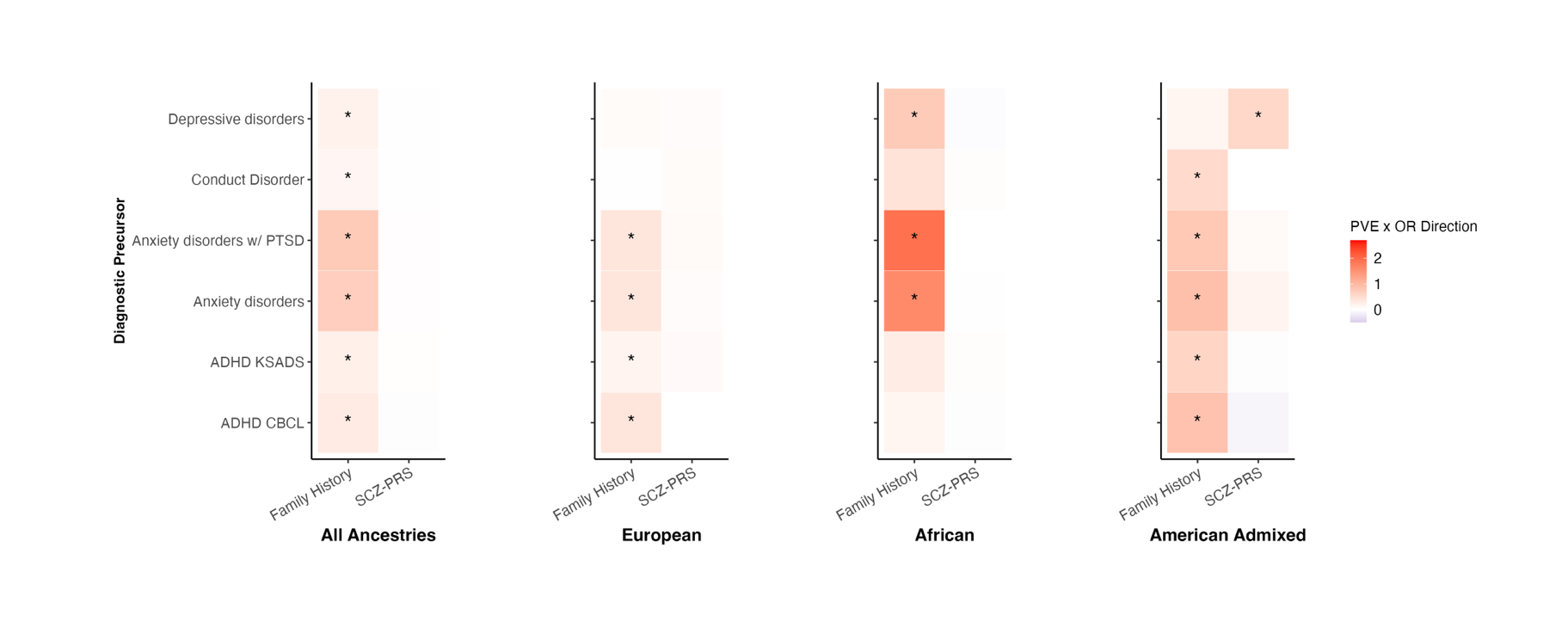


* FDR P < 0.05

Table S1. The number of participants with data available per phenotype measure for each ancestry

| Phenotype Measure | European | African | Admixed American | Cross-Ancestry (Total) |
| --- | --- | --- | --- | --- |
| NIH-TB Cognition | 5,452 | 2,020 | 1,425 | 8,897 |
| CBCL | 5,633 | 2,093 | 1,474 | 9,200 |
| PQB | 5,636 | 2,093 | 1,477 | 9,206 |
| KSADS-COMP | 5,621 | 2,087 | 1,471 | 9,179 |

Table S2. Endorsement of family history of psychosis (SCZ-FH) for each ancestry

| **Ancestry** | **N with SCZ-FH > 0** | **Total N** | **Percent with SCZ-FH > 0 (%)** |
| --- | --- | --- | --- |
| All | 776 | 9206 | 8.4 |
| EUR-only | 392 | 5636 | 7.0 |
| AFR-only | 244 | 2093 | 11.7 |
| AMR-only | 140 | 1477 | 9.5 |

Table S3. Mean and standard deviation of income-to-needs ratio for each ancestry and the cross-ancestry analysis

| **Ancestry** | **Mean SES** | **SD SES** |
| --- | --- | --- |
| All | 3.67 | 2.43 |
| EUR-only | 4.51 | 2.23 |
| AFR-only | 1.97 | 1.98 |
| AMR-only | 2.37 | 2.02 |

Table S4A. Descriptive statistics for dimensional outcome measures for each ancestry group and across all ancestry groups.

| **Precursor** | **All Mean (SD)** | **EUR-only Mean (SD)** | **AFR-only Mean (SD)** | **AMR-only Mean (SD)** |
| --- | --- | --- | --- | --- |
| NIH Total Cognitive Score (z-scored) | -0.04 (1.00) | 0.27 (0.91) | -0.67 (0.89) | -0.31 (0.92) |
| CBCL - Total | 45.97 (11.33) | 45.82 (10.77) | 46.28 (12.70) | 46.13 (11.36) |
| CBCL - Anxious Depressed | 53.48 (5.91) | 53.65 (6.03) | 52.88 (5.55) | 53.68 (5.88) |
| CBCL - Withdrawn | 53.54 (5.86) | 53.26 (5.51) | 53.91 (6.36) | 54.11 (6.33) |
| CBC - Somatic | 54.88 (6.03) | 54.92 (5.92) | 54.73 (6.10) | 54.94 (6.35) |
| CBCL - Social | 52.81 (4.71) | 52.49 (4.44) | 53.53 (5.28) | 53.01 (4.75) |
| CBCL - Thought | 53.84 (5.96) | 53.97 (5.89) | 53.79 (6.28) | 53.42 (5.72) |
| CBCL - Attention | 53.93 (6.20) | 53.65 (5.90) | 54.75 (6.93) | 53.82 (6.12) |
| CBCL - Rule Breaking | 52.83 (4.93) | 52.35 (4.38) | 54.15 (6.17) | 52.80 (4.60) |
| CBCL - Aggressive | 52.89 (5.54) | 52.68 (5.20) | 53.50 (6.41) | 52.80 (5.38) |
| PQB - Distress Score | 6.35 (10.60) | 5.00 (9.07) | 8.84 (12.72) | 7.94 (11.77) |

Table S4B. Endorsement rates for diagnostic outcome measures for each ancestry groups and across all ancestry groups.

| **Precursor** | **All N Positive** | **EUR-only N Positive** | **AFR-only N Positive** | **AMR-only N Positive** | |
| --- | --- | --- | --- | --- | --- |
| KSADS - ADHD | 1928 (21%) | 1156 (20.6%) | 503 (24.1%) | 269 (18.3%) |  |
| CBCL - ADHD | 686 (7.5%) | 365 (6.5%) | 216 (10.3%) | 105 (7.1%) |  |
| KSADS - Anxiety Disorders | 1364 (14.9%) | 939 (16.7%) | 260 (12.5%) | 165 (11.2%) |  |
| KSADS - Depressive Disorders | 499 (5.4%) | 241 (4.3%) | 170 (8.1%) | 88 (6.0%) |  |
| KSADS - Conduct Disorder | 1420 (15.5%) | 935 (16.6%) | 334 (16%) | 151 (10.3%) |  |
| KSADS - Anxiety disorders w/ PTSD | 1421 (15.5%) | 965 (17.2%) | 285 (13.7%) | 171 (11.6%) |  |
|  |  |  |  |  |  |

Table S5. Independent associations of schizophrenia polygenic risk scores (SCZ-PRS) and family history of psychosis (SCZ-FH) with diagnostic phenotypes derived from the Kiddie Schedule for Affective Disorders and Schizophrenia (KSADS) or Child Behavior Checklist (CBCL). FDR P refers to the false discovery rate–corrected p value; PVE refers to the percentage of variance explained.

| **Ancestry** | **Genetic Risk Measure** | **Precursor** | **OR** | **SE** | **95% CI** | **Nominal P** | **FDR P** | **PVE (%)** |
| --- | --- | --- | --- | --- | --- | --- | --- | --- |
| All | Family History | KSADS - ADHD | 1.13 | 0.02 | 1.08 - 1.19 | <0.001 | **<0.001** | 0.35 |
|  |  | CBCL - ADHD | 1.20 | 0.03 | 1.14 - 1.27 | <0.001 | **<0.001** | 0.63 |
|  |  | KSADS - Anxiety Disorders | 1.21 | 0.02 | 1.16 - 1.27 | <0.001 | **<0.001** | 0.89 |
|  |  | KSADS - Anxiety disorders w/ PTSD | 1.22 | 0.02 | 1.17 - 1.28 | <0.001 | **<0.001** | 0.99 |
|  |  | KSADS - Depressive Disorders | 1.14 | 0.03 | 1.07 - 1.22 | <0.001 | **<0.001** | 0.24 |
|  |  | KSADS - Conduct Disorder | 1.10 | 0.03 | 1.05 - 1.16 | <0.001 | **<0.001** | 0.18 |
|  |  |  |  |  |  |  |  |  |
|  | SCZ PRS | KSADS - ADHD | 1.03 | 0.03 | 0.98 - 1.08 | 0.293 | 0.440 | 0.01 |
|  |  | CBCL - ADHD | 0.96 | 0.04 | 0.88 - 1.04 | 0.277 | 0.440 | 0.02 |
|  |  | KSADS - Anxiety Disorders | 1.03 | 0.03 | 0.97 - 1.09 | 0.356 | 0.455 | 0.01 |
|  |  | KSADS - Anxiety disorders w/ PTSD | 1.03 | 0.03 | 0.97 - 1.09 | 0.379 | 0.455 | 0.01 |
|  |  | KSADS - Depressive Disorders | 1.03 | 0.05 | 0.94 - 1.13 | 0.486 | 0.530 | 0.01 |
|  |  | KSADS - Conduct Disorder | 1.01 | 0.03 | 0.95 - 1.07 | 0.707 | 0.707 | 0.00 |
|  |  |  |  |  |  |  |  |  |
| European | Family History | KSADS - ADHD | 1.14 | 0.04 | 1.05 - 1.23 | 0.001 | **0.003** | 0.23 |
|  |  | CBCL - ADHD | 1.27 | 0.05 | 1.15 - 1.39 | <0.001 | **<0.001** | 0.57 |
|  |  | KSADS - Anxiety Disorders | 1.19 | 0.04 | 1.10 - 1.29 | <0.001 | **<0.001** | 0.43 |
|  |  | KSADS - Anxiety disorders w/ PTSD | 1.19 | 0.04 | 1.10 - 1.29 | <0.001 | **<0.001** | 0.44 |
|  |  | KSADS - Depressive Disorders | 1.14 | 0.07 | 1.00 - 1.30 | 0.050 | 0.100 | 0.09 |
|  |  | KSADS - Conduct Disorder | 1.04 | 0.04 | 0.96 - 1.14 | 0.343 | 0.374 | 0.02 |
|  |  |  |  |  |  |  |  |  |
|  | SCZ PRS | KSADS - ADHD | 1.08 | 0.03 | 1.01 - 1.16 | 0.024 | 0.058 | 0.10 |
|  |  | CBCL - ADHD | 1.02 | 0.06 | 0.91 - 1.14 | 0.720 | 0.720 | 0.00 |
|  |  | KSADS - Anxiety Disorders | 1.06 | 0.04 | 0.99 - 1.14 | 0.118 | 0.142 | 0.05 |
|  |  | KSADS - Anxiety disorders w/ PTSD | 1.07 | 0.04 | 0.99 - 1.15 | 0.079 | 0.130 | 0.07 |
|  |  | KSADS - Depressive Disorders | 1.11 | 0.07 | 0.97 - 1.27 | 0.115 | 0.142 | 0.06 |
|  |  | KSADS - Conduct Disorder | 1.07 | 0.04 | 0.99 - 1.15 | 0.086 | 0.130 | 0.06 |
|  |  |  |  |  |  |  |  |  |
| African | Family History | KSADS - ADHD | 1.11 | 0.04 | 1.04 - 1.19 | 0.003 | **0.006** | 0.50 |
|  |  | CBCL - ADHD | 1.15 | 0.04 | 1.06 - 1.25 | 0.001 | **0.003** | 0.78 |
|  |  | KSADS - Anxiety Disorders | 1.22 | 0.04 | 1.14 - 1.32 | <0.001 | **<0.001** | 2.05 |
|  |  | KSADS - Anxiety disorders w/ PTSD | 1.25 | 0.04 | 1.16 - 1.35 | <0.001 | **<0.001** | 2.57 |
|  |  | KSADS - Depressive Disorders | 1.15 | 0.04 | 1.06 - 1.26 | 0.001 | **0.003** | 0.74 |
|  |  | KSADS - Conduct Disorder | 1.13 | 0.04 | 1.05 - 1.22 | 0.001 | **0.003** | 0.64 |
|  |  |  |  |  |  |  |  |  |
|  | SCZ PRS | KSADS - ADHD | 1.01 | 0.07 | 0.89 - 1.15 | 0.873 | 0.932 | 0.00 |
|  |  | CBCL - ADHD | 0.98 | 0.09 | 0.82 - 1.17 | 0.812 | 0.932 | 0.00 |
|  |  | KSADS - Anxiety Disorders | 1.03 | 0.08 | 0.88 - 1.21 | 0.698 | 0.932 | 0.01 |
|  |  | KSADS - Anxiety disorders w/ PTSD | 1.01 | 0.08 | 0.86 - 1.17 | 0.932 | 0.932 | 0.00 |
|  |  | KSADS - Depressive Disorders | 0.89 | 0.10 | 0.74 - 1.08 | 0.245 | 0.420 | 0.08 |
|  |  | KSADS - Conduct Disorder | 1.02 | 0.07 | 0.88 - 1.18 | 0.806 | 0.932 | 0.00 |
|  |  |  |  |  |  |  |  |  |
| American Admixed | Family History | KSADS - ADHD | 1.19 | 0.06 | 1.06 - 1.34 | 0.003 | **0.010** | 0.72 |
|  |  | CBCL - ADHD | 1.25 | 0.07 | 1.08 - 1.44 | 0.003 | **0.010** | 0.82 |
|  |  | KSADS - Anxiety Disorders | 1.24 | 0.06 | 1.09 - 1.41 | 0.001 | **0.009** | 1.05 |
|  |  | KSADS - Anxiety disorders w/ PTSD | 1.23 | 0.06 | 1.08 - 1.39 | 0.001 | **0.009** | 0.93 |
|  |  | KSADS - Depressive Disorders | 1.13 | 0.09 | 0.95 - 1.34 | 0.154 | 0.264 | 0.16 |
|  |  | KSADS - Conduct Disorder | 1.15 | 0.07 | 1.00 - 1.33 | 0.046 | 0.092 | 0.33 |
|  |  |  |  |  |  |  |  |  |
|  | SCZ PRS | KSADS - ADHD | 0.95 | 0.07 | 0.82 - 1.10 | 0.503 | 0.670 | 0.04 |
|  |  | CBCL - ADHD | 0.91 | 0.11 | 0.74 - 1.13 | 0.406 | 0.608 | 0.06 |
|  |  | KSADS - Anxiety Disorders | 1.04 | 0.09 | 0.87 - 1.24 | 0.657 | 0.717 | 0.02 |
|  |  | KSADS - Anxiety disorders w/ PTSD | 1.01 | 0.09 | 0.85 - 1.21 | 0.873 | 0.873 | 0.00 |
|  |  | KSADS - Depressive Disorders | 1.37 | 0.12 | 1.08 - 1.74 | 0.008 | **0.020** | 0.53 |
|  |  | KSADS - Conduct Disorder | 0.96 | 0.10 | 0.79 - 1.15 | 0.643 | 0.717 | 0.02 |

Table S6. Independent associations of schizophrenia polygenic risk scores (SCZ-PRS) and family history of psychosis (SCZ-FH) with diagnostic phenotypes derived from the Kiddie Schedule for Affective Disorders and Schizophrenia (KSADS) or Child Behavior Checklist (CBCL), including income-to-needs as a covariate. FDR P refers to the false discovery rate–corrected p value; PVE refers to the percentage of variance explained.

| **Ancestry** | **Genetic Risk Measure** | **Precursor** | **OR** | **SE** | **95% CI** | **Nominal P** | **FDR P** | **PVE (%)** |
| --- | --- | --- | --- | --- | --- | --- | --- | --- |
| All | Family History | KSADS - ADHD | 1.11 | 0.03 | 1.05 - 1.16 | <0.001 | **<0.001** | 0.22 |
|  |  | CBCL - ADHD | 1.14 | 0.03 | 1.07 - 1.22 | <0.001 | **<0.001** | 0.28 |
|  |  | KSADS - Anxiety Disorders | 1.19 | 0.03 | 1.13 - 1.25 | <0.001 | **<0.001** | 0.69 |
|  |  | KSADS - Anxiety disorders w/ PTSD | 1.20 | 0.03 | 1.14 - 1.26 | <0.001 | **<0.001** | 0.74 |
|  |  | KSADS - Depressive Disorders | 1.13 | 0.03 | 1.06 - 1.21 | <0.001 | **0.001** | 0.19 |
|  |  | KSADS - Conduct Disorder | 1.09 | 0.03 | 1.03 - 1.15 | 0.003 | **0.006** | 0.13 |
|  |  |  |  |  |  |  |  |  |
|  | SCZ PRS | KSADS - ADHD | 1.04 | 0.03 | 0.98 - 1.09 | 0.209 | 0.279 | 0.02 |
|  |  | CBCL - ADHD | 0.95 | 0.04 | 0.88 - 1.04 | 0.276 | 0.331 | 0.02 |
|  |  | KSADS - Anxiety Disorders | 1.05 | 0.03 | 0.98 - 1.11 | 0.152 | 0.229 | 0.03 |
|  |  | KSADS - Anxiety disorders w/ PTSD | 1.05 | 0.03 | 0.98 - 1.11 | 0.150 | 0.229 | 0.03 |
|  |  | KSADS - Depressive Disorders | 1.04 | 0.05 | 0.94 - 1.15 | 0.422 | 0.422 | 0.01 |
|  |  | KSADS - Conduct Disorder | 1.03 | 0.03 | 0.97 - 1.09 | 0.360 | 0.393 | 0.01 |
|  |  |  |  |  |  |  |  |  |
| European | Family History | KSADS - ADHD | 1.12 | 0.04 | 1.03 - 1.21 | 0.006 | **0.019** | 0.16 |
|  |  | CBCL - ADHD | 1.22 | 0.05 | 1.10 - 1.35 | <0.001 | **<0.001** | 0.35 |
|  |  | KSADS - Anxiety Disorders | 1.18 | 0.04 | 1.08 - 1.27 | <0.001 | **<0.001** | 0.36 |
|  |  | KSADS - Anxiety disorders w/ PTSD | 1.18 | 0.04 | 1.08 - 1.27 | <0.001 | **<0.001** | 0.36 |
|  |  | KSADS - Depressive Disorders | 1.12 | 0.07 | 0.98 - 1.28 | 0.110 | 0.147 | 0.06 |
|  |  | KSADS - Conduct Disorder | 1.04 | 0.05 | 0.95 - 1.14 | 0.413 | 0.450 | 0.01 |
|  |  |  |  |  |  |  |  |  |
|  | SCZ PRS | KSADS - ADHD | 1.07 | 0.04 | 1.00 - 1.15 | 0.062 | 0.124 | 0.08 |
|  |  | CBCL - ADHD | 1.01 | 0.06 | 0.91 - 1.13 | 0.802 | 0.802 | 0.00 |
|  |  | KSADS - Anxiety Disorders | 1.07 | 0.04 | 0.99 - 1.15 | 0.085 | 0.145 | 0.07 |
|  |  | KSADS - Anxiety disorders w/ PTSD | 1.08 | 0.04 | 1.00 - 1.16 | 0.049 | 0.118 | 0.09 |
|  |  | KSADS - Depressive Disorders | 1.11 | 0.07 | 0.97 - 1.27 | 0.131 | 0.157 | 0.06 |
|  |  | KSADS - Conduct Disorder | 1.07 | 0.04 | 0.99 - 1.15 | 0.096 | 0.145 | 0.06 |
|  |  |  |  |  |  |  |  |  |
| African | Family History | KSADS - ADHD | 1.08 | 0.04 | 1.00 - 1.17 | 0.047 | 0.114 | 0.27 |
|  |  | CBCL - ADHD | 1.07 | 0.05 | 0.97 - 1.18 | 0.173 | 0.347 | 0.15 |
|  |  | KSADS - Anxiety Disorders | 1.20 | 0.04 | 1.10 - 1.30 | <0.001 | **<0.001** | 1.62 |
|  |  | KSADS - Anxiety disorders w/ PTSD | 1.21 | 0.04 | 1.12 - 1.31 | <0.001 | **<0.001** | 1.92 |
|  |  | KSADS - Depressive Disorders | 1.15 | 0.05 | 1.05 - 1.27 | 0.002 | **0.009** | 0.73 |
|  |  | KSADS - Conduct Disorder | 1.10 | 0.04 | 1.02 - 1.19 | 0.019 | 0.058 | 0.41 |
|  |  |  |  |  |  |  |  |  |
|  | SCZ PRS | KSADS - ADHD | 1.05 | 0.07 | 0.91 - 1.21 | 0.502 | 0.687 | 0.03 |
|  |  | CBCL - ADHD | 0.95 | 0.10 | 0.78 - 1.16 | 0.644 | 0.749 | 0.02 |
|  |  | KSADS - Anxiety Disorders | 1.04 | 0.09 | 0.87 - 1.23 | 0.687 | 0.749 | 0.01 |
|  |  | KSADS - Anxiety disorders w/ PTSD | 1.01 | 0.09 | 0.86 - 1.20 | 0.891 | 0.891 | 0.00 |
|  |  | KSADS - Depressive Disorders | 0.93 | 0.11 | 0.75 - 1.15 | 0.502 | 0.687 | 0.03 |
|  |  | KSADS - Conduct Disorder | 1.06 | 0.08 | 0.90 - 1.24 | 0.515 | 0.687 | 0.03 |
|  |  |  |  |  |  |  |  |  |
| American Admixed | Family History | KSADS - ADHD | 1.17 | 0.07 | 1.03 - 1.34 | 0.016 | **0.038** | 0.58 |
|  |  | CBCL - ADHD | 1.25 | 0.08 | 1.06 - 1.47 | 0.006 | **0.029** | 0.84 |
|  |  | KSADS - Anxiety Disorders | 1.23 | 0.07 | 1.07 - 1.41 | 0.004 | **0.029** | 0.91 |
|  |  | KSADS - Anxiety disorders w/ PTSD | 1.21 | 0.07 | 1.05 - 1.39 | 0.007 | **0.029** | 0.78 |
|  |  | KSADS - Depressive Disorders | 1.13 | 0.09 | 0.94 - 1.35 | 0.199 | 0.299 | 0.15 |
|  |  | KSADS - Conduct Disorder | 1.19 | 0.08 | 1.03 - 1.39 | 0.022 | **0.045** | 0.53 |
|  |  |  |  |  |  |  |  |  |
|  | SCZ PRS | KSADS - ADHD | 0.97 | 0.08 | 0.83 - 1.14 | 0.740 | 0.808 | 0.01 |
|  |  | CBCL - ADHD | 0.90 | 0.12 | 0.71 - 1.13 | 0.362 | 0.434 | 0.09 |
|  |  | KSADS - Anxiety Disorders | 1.13 | 0.10 | 0.94 - 1.37 | 0.200 | 0.299 | 0.17 |
|  |  | KSADS - Anxiety disorders w/ PTSD | 1.10 | 0.10 | 0.91 - 1.32 | 0.331 | 0.434 | 0.10 |
|  |  | KSADS - Depressive Disorders | 1.38 | 0.13 | 1.07 - 1.77 | 0.012 | **0.036** | 0.56 |
|  |  | KSADS - Conduct Disorder | 1.02 | 0.10 | 0.83 - 1.25 | 0.874 | 0.874 | 0.00 |

Table S7. Joint model associations of schizophrenia polygenic risk scores (SCZ-PRS) and family history of psychosis (SCZ-FH) with diagnostic phenotypes derived from the Kiddie Schedule for Affective Disorders and Schizophrenia (KSADS) or Child Behavior Checklist (CBCL). FDR P refers to the false discovery rate–corrected p value; PVE refers to the percentage of variance explained.

| **Ancestry** | **Precursor** | **Family History of Psychosis (Joint Model)** | | | | | | |  | | **SCZ PRS (Joint Model)** | | | | | |  | | **Joint PVE** | |  |  |
| --- | --- | --- | --- | --- | --- | --- | --- | --- | --- | --- | --- | --- | --- | --- | --- | --- | --- | --- | --- | --- | --- | --- |
|  |  | *OR* | *SE* | *95% CI* | *Nominal P* | *FDR P* | *PVE* |  | | *OR* | | *SE* | *95% CI* | *Nominal P* | *FDR P* | *PVE* | |  | |  | | |
| All | KSADS - ADHD | 1.13 | 0.02 | 1.08 - 1.19 | <0.001 | **<0.001** | 0.35 |  | | 1.03 | | 0.03 | 0.97 - 1.08 | 0.331 | 0.497 | 0.01 | |  | | 0.36 | |  |
|  | CBCL - ADHD | 1.20 | 0.03 | 1.14 - 1.27 | <0.001 | **<0.001** | 0.63 |  | | 0.95 | | 0.04 | 0.88 - 1.03 | 0.252 | 0.433 | 0.02 | |  | | 0.65 | |  |
|  | KSADS - Anxiety Disorders | 1.21 | 0.02 | 1.15 - 1.27 | <0.001 | **<0.001** | 0.88 |  | | 1.02 | | 0.03 | 0.96 - 1.09 | 0.435 | 0.557 | 0.01 | |  | | 0.89 | |  |
|  | KSADS - Anxiety disorders w/ PTSD | 1.22 | 0.02 | 1.17 - 1.28 | <0.001 | **<0.001** | 0.99 |  | | 1.02 | | 0.03 | 0.96 - 1.08 | 0.465 | 0.557 | 0.01 | |  | | 1.00 | |  |
|  | KSADS - Depressive Disorders | 1.14 | 0.03 | 1.07 - 1.22 | <0.001 | **<0.001** | 0.24 |  | | 1.03 | | 0.05 | 0.94 - 1.13 | 0.510 | 0.557 | 0.01 | |  | | 0.25 | |  |
|  | KSADS - Conduct Disorder | 1.10 | 0.03 | 1.05 - 1.16 | <0.001 | **<0.001** | 0.18 |  | | 1.01 | | 0.03 | 0.95 - 1.07 | 0.754 | 0.754 | 0.00 | |  | | 0.18 | |  |
|  |  |  |  |  |  |  |  |  | |  | |  |  |  |  |  | |  | |  | |  |
| European | KSADS - ADHD | 1.13 | 0.04 | 1.05 - 1.22 | 0.002 | **0.005** | 0.21 |  | | 1.08 | | 0.03 | 1.01 - 1.15 | 0.034 | 0.083 | 0.09 | |  | | 0.32 | |  |
|  | CBCL - ADHD | 1.27 | 0.05 | 1.15 - 1.39 | <0.001 | **<0.001** | 0.57 |  | | 1.01 | | 0.06 | 0.90 - 1.13 | 0.875 | 0.875 | 0.00 | |  | | 0.57 | |  |
|  | KSADS - Anxiety Disorders | 1.19 | 0.04 | 1.10 - 1.28 | <0.001 | **<0.001** | 0.41 |  | | 1.05 | | 0.04 | 0.98 - 1.13 | 0.168 | 0.202 | 0.04 | |  | | 0.47 | |  |
|  | KSADS - Anxiety disorders w/ PTSD | 1.19 | 0.04 | 1.10 - 1.28 | <0.001 | **<0.001** | 0.42 |  | | 1.06 | | 0.04 | 0.99 - 1.14 | 0.116 | 0.175 | 0.05 | |  | | 0.49 | |  |
|  | KSADS - Depressive Disorders | 1.14 | 0.07 | 0.99 - 1.30 | 0.060 | 0.119 | 0.09 |  | | 1.11 | | 0.07 | 0.97 - 1.26 | 0.134 | 0.179 | 0.05 | |  | | 0.15 | |  |
|  | KSADS - Conduct Disorder | 1.04 | 0.05 | 0.95 - 1.14 | 0.384 | 0.419 | 0.02 |  | | 1.06 | | 0.04 | 0.99 - 1.15 | 0.094 | 0.161 | 0.06 | |  | | 0.08 | |  |
|  |  |  |  |  |  |  |  |  | |  | |  |  |  |  |  | |  | |  | |  |
| African | KSADS - ADHD | 1.11 | 0.04 | 1.04 - 1.19 | 0.003 | **0.006** | 0.50 |  | | 1.01 | | 0.07 | 0.89 - 1.15 | 0.900 | 0.973 | 0.00 | |  | | 0.50 | |  |
|  | CBCL - ADHD | 1.15 | 0.04 | 1.06 - 1.25 | <0.001 | **0.003** | 0.78 |  | | 0.98 | | 0.09 | 0.82 - 1.16 | 0.778 | 0.973 | 0.00 | |  | | 0.78 | |  |
|  | KSADS - Anxiety Disorders | 1.22 | 0.04 | 1.14 - 1.32 | <0.001 | **<0.001** | 2.04 |  | | 1.02 | | 0.08 | 0.87 - 1.20 | 0.770 | 0.973 | 0.01 | |  | | 2.05 | |  |
|  | KSADS - Anxiety disorders w/ PTSD | 1.25 | 0.04 | 1.16 - 1.35 | <0.001 | **<0.001** | 2.57 |  | | 1.00 | | 0.08 | 0.85 - 1.16 | 0.973 | 0.973 | 0.00 | |  | | 2.57 | |  |
|  | KSADS - Depressive Disorders | 1.16 | 0.04 | 1.06 - 1.26 | <0.001 | **0.003** | 0.74 |  | | 0.89 | | 0.10 | 0.73 - 1.08 | 0.224 | 0.384 | 0.09 | |  | | 0.83 | |  |
|  | KSADS - Conduct Disorder | 1.13 | 0.04 | 1.05 - 1.22 | 0.001 | **0.003** | 0.64 |  | | 1.02 | | 0.07 | 0.88 - 1.18 | 0.830 | 0.973 | 0.00 | |  | | 0.64 | |  |
|  |  |  |  |  |  |  |  |  | |  | |  |  |  |  |  | |  | |  | |  |
| American Admixed | KSADS - ADHD | 1.19 | 0.06 | 1.06 - 1.34 | 0.003 | **0.009** | 0.73 |  | | 0.95 | | 0.07 | 0.82 - 1.10 | 0.462 | 0.616 | 0.05 | |  | | 0.76 | |  |
|  | CBCL - ADHD | 1.25 | 0.07 | 1.08 - 1.45 | 0.003 | **0.009** | 0.84 |  | | 0.91 | | 0.11 | 0.73 - 1.12 | 0.359 | 0.539 | 0.08 | |  | | 0.90 | |  |
|  | KSADS - Anxiety Disorders | 1.24 | 0.06 | 1.09 - 1.41 | <0.001 | **0.009** | 1.05 |  | | 1.04 | | 0.09 | 0.87 - 1.24 | 0.682 | 0.744 | 0.01 | |  | | 1.07 | |  |
|  | KSADS - Anxiety disorders w/ PTSD | 1.23 | 0.06 | 1.08 - 1.39 | 0.002 | **0.009** | 0.93 |  | | 1.01 | | 0.09 | 0.85 - 1.20 | 0.905 | 0.905 | 0.00 | |  | | 0.93 | |  |
|  | KSADS - Depressive Disorders | 1.13 | 0.09 | 0.95 - 1.35 | 0.167 | 0.286 | 0.15 |  | | 1.37 | | 0.12 | 1.08 - 1.73 | 0.009 | **0.021** | 0.52 | |  | | 0.68 | |  |
|  | KSADS - Conduct Disorder | 1.15 | 0.07 | 1.00 - 1.33 | 0.044 | 0.089 | 0.34 |  | | 0.95 | | 0.09 | 0.79 - 1.15 | 0.601 | 0.722 | 0.02 | |  | | 0.36 | |  |

Table S8. Joint model associations of schizophrenia polygenic risk scores (SCZ-PRS) and family history of psychosis (SCZ-FH) with diagnostic phenotypes derived from the Kiddie Schedule for Affective Disorders and Schizophrenia (KSADS) or Child Behavior Checklist (CBCL), including income-to-needs as a covariate. FDR P refers to the false discovery rate–corrected p value; PVE refers to the percentage of variance explained.

| **Ancestry** | **Precursor** | **Family History of Psychosis (Joint Model)** | | | | |  | |  | | | **SCZ PRS (Joint Model)** | | | | |  | |  | | **Joint PVE** | | |  |
| --- | --- | --- | --- | --- | --- | --- | --- | --- | --- | --- | --- | --- | --- | --- | --- | --- | --- | --- | --- | --- | --- | --- | --- | --- |
|  |  | **OR** | **SE** | **95% CI** | **Nominal P** | **FDR P** | | **PVE** | |  | **OR** | | **SE** | **95% CI** | **Nominal P** | **FDR P** | | **PVE** | |  | |  | | |
| All | KSADS - ADHD | 1.10 | 0.03 | 1.05 - 1.16 | <0.001 | <0.001 | | 0.22 | |  | 1.03 | | 0.03 | 0.98 - 1.09 | 0.240 | 0.302 | | 0.02 | |  | | 0.24 |  |  |
|  | CBCL - ADHD | 1.14 | 0.03 | 1.07 - 1.22 | <0.001 | <0.001 | | 0.28 | |  | 0.95 | | 0.04 | 0.87 - 1.04 | 0.252 | 0.302 | | 0.02 | |  | | 0.30 |  |  |
|  | KSADS - Anxiety Disorders | 1.19 | 0.03 | 1.13 - 1.25 | <0.001 | <0.001 | | 0.69 | |  | 1.04 | | 0.03 | 0.98 - 1.11 | 0.198 | 0.298 | | 0.02 | |  | | 0.72 |  |  |
|  | KSADS - Anxiety disorders w/ PTSD | 1.20 | 0.03 | 1.14 - 1.26 | <0.001 | <0.001 | | 0.74 | |  | 1.04 | | 0.03 | 0.98 - 1.11 | 0.197 | 0.298 | | 0.02 | |  | | 0.77 |  |  |
|  | KSADS - Depressive Disorders | 1.13 | 0.03 | 1.06 - 1.21 | <0.001 | 0.001 | | 0.19 | |  | 1.04 | | 0.05 | 0.94 - 1.15 | 0.450 | 0.450 | | 0.01 | |  | | 0.19 |  |  |
|  | KSADS - Conduct Disorder | 1.09 | 0.03 | 1.03 - 1.15 | 0.003 | 0.007 | | 0.13 | |  | 1.03 | | 0.03 | 0.97 - 1.09 | 0.396 | 0.432 | | 0.01 | |  | | 0.14 |  |  |
|  |  |  |  |  |  |  | |  | |  |  | |  |  |  |  | |  | |  | |  |  |  |
| European | KSADS - ADHD | 1.11 | 0.04 | 1.03 - 1.21 | 0.008 | 0.024 | | 0.16 | |  | 1.06 | | 0.04 | 0.99 - 1.14 | 0.079 | 0.157 | | 0.07 | |  | | 0.23 |  |  |
|  | CBCL - ADHD | 1.22 | 0.05 | 1.10 - 1.35 | <0.001 | <0.001 | | 0.35 | |  | 1.01 | | 0.06 | 0.90 - 1.13 | 0.923 | 0.923 | | 0.00 | |  | | 0.35 |  |  |
|  | KSADS - Anxiety Disorders | 1.17 | 0.04 | 1.08 - 1.27 | <0.001 | <0.001 | | 0.35 | |  | 1.06 | | 0.04 | 0.99 - 1.14 | 0.117 | 0.167 | | 0.05 | |  | | 0.42 |  |  |
|  | KSADS - Anxiety disorders w/ PTSD | 1.17 | 0.04 | 1.08 - 1.27 | <0.001 | <0.001 | | 0.35 | |  | 1.07 | | 0.04 | 0.99 - 1.15 | 0.070 | 0.157 | | 0.07 | |  | | 0.44 |  |  |
|  | KSADS - Depressive Disorders | 1.11 | 0.07 | 0.97 - 1.27 | 0.125 | 0.167 | | 0.06 | |  | 1.11 | | 0.07 | 0.97 - 1.27 | 0.146 | 0.175 | | 0.05 | |  | | 0.11 |  |  |
|  | KSADS - Conduct Disorder | 1.04 | 0.05 | 0.95 - 1.13 | 0.454 | 0.495 | | 0.01 | |  | 1.06 | | 0.04 | 0.99 - 1.15 | 0.103 | 0.167 | | 0.06 | |  | | 0.07 |  |  |
|  |  |  |  |  |  |  | |  | |  |  | |  |  |  |  | |  | |  | |  |  |  |
| African | KSADS - ADHD | 1.08 | 0.04 | 1.00 - 1.16 | 0.049 | 0.117 | | 0.26 | |  | 1.05 | | 0.07 | 0.91 - 1.21 | 0.520 | 0.714 | | 0.03 | |  | | 0.29 |  |  |
|  | CBCL - ADHD | 1.07 | 0.05 | 0.97 - 1.18 | 0.172 | 0.344 | | 0.15 | |  | 0.95 | | 0.10 | 0.78 - 1.16 | 0.635 | 0.762 | | 0.02 | |  | | 0.16 |  |  |
|  | KSADS - Anxiety Disorders | 1.20 | 0.04 | 1.10 - 1.30 | <0.001 | <0.001 | | 1.61 | |  | 1.03 | | 0.09 | 0.86 - 1.22 | 0.753 | 0.821 | | 0.01 | |  | | 1.62 |  |  |
|  | KSADS - Anxiety disorders w/ PTSD | 1.21 | 0.04 | 1.12 - 1.31 | <0.001 | <0.001 | | 1.92 | |  | 1.00 | | 0.09 | 0.85 - 1.19 | 0.979 | 0.979 | | 0.00 | |  | | 1.92 |  |  |
|  | KSADS - Depressive Disorders | 1.16 | 0.05 | 1.05 - 1.27 | 0.002 | 0.008 | | 0.73 | |  | 0.92 | | 0.11 | 0.74 - 1.14 | 0.460 | 0.714 | | 0.04 | |  | | 0.76 |  |  |
|  | KSADS - Conduct Disorder | 1.10 | 0.04 | 1.02 - 1.19 | 0.020 | 0.060 | | 0.41 | |  | 1.05 | | 0.08 | 0.90 - 1.24 | 0.535 | 0.714 | | 0.03 | |  | | 0.44 |  |  |
|  |  |  |  |  |  |  | |  | |  |  | |  |  |  |  | |  | |  | |  |  |  |
| American Admixed | KSADS - ADHD | 1.18 | 0.07 | 1.03 - 1.34 | 0.015 | 0.036 | | 0.59 | |  | 0.97 | | 0.08 | 0.83 - 1.13 | 0.683 | 0.745 | | 0.02 | |  | | 0.60 |  |  |
|  | CBCL - ADHD | 1.25 | 0.08 | 1.07 - 1.47 | 0.006 | 0.031 | | 0.86 | |  | 0.89 | | 0.12 | 0.70 - 1.12 | 0.324 | 0.425 | | 0.11 | |  | | 0.95 |  |  |
|  | KSADS - Anxiety Disorders | 1.23 | 0.07 | 1.07 - 1.41 | 0.004 | 0.031 | | 0.89 | |  | 1.13 | | 0.10 | 0.93 - 1.36 | 0.213 | 0.320 | | 0.15 | |  | | 1.06 |  |  |
|  | KSADS - Anxiety disorders w/ PTSD | 1.21 | 0.07 | 1.05 - 1.39 | 0.008 | 0.031 | | 0.77 | |  | 1.09 | | 0.09 | 0.91 - 1.31 | 0.354 | 0.425 | | 0.08 | |  | | 0.86 |  |  |
|  | KSADS - Depressive Disorders | 1.13 | 0.10 | 0.93 - 1.36 | 0.213 | 0.320 | | 0.15 | |  | 1.37 | | 0.13 | 1.07 - 1.76 | 0.012 | 0.036 | | 0.56 | |  | | 0.70 |  |  |
|  | KSADS - Conduct Disorder | 1.19 | 0.08 | 1.02 - 1.39 | 0.023 | 0.046 | | 0.52 | |  | 1.01 | | 0.10 | 0.82 - 1.23 | 0.944 | 0.944 | | 0.00 | |  | | 0.53 |  |  |

Table S9. Independent associations of schizophrenia polygenic risk scores (SCZ-PRS) and family history of psychosis (SCZ-FH) with dimensional, total cognitive score derived from the NIH-Toolbox, Child Behavior Checklist (CBCL) scores, and prodromal questionnaire (PQB) scores), including family conflict as a covariate. FDR P refers to the false discovery rate–corrected p value; PVE refers to the percentage of variance explained.

| **Ancestry** | **Genetic Risk Measure** | **Precursor** | **B** | **SE** | **95% CI** | **Nominal P** | **FDR P** | **PVE (%)** |
| --- | --- | --- | --- | --- | --- | --- | --- | --- |
| All | Family History | NIH Total Cognitive Score | -0.11 | 0.18 | -0.46 - 0.24 | 0.537 | 0.656 | 0.00 |
|  |  | CBCL - Total | 1.11 | 0.12 | 0.87 - 1.35 | <0.001 | **<0.001** | 0.90 |
|  |  | CBCL - Anxious Depressed | 0.40 | 0.06 | 0.28 - 0.53 | <0.001 | **<0.001** | 0.44 |
|  |  | CBCL - Withdrawn | 0.43 | 0.06 | 0.31 - 0.55 | <0.001 | **<0.001** | 0.51 |
|  |  | CBCL - Somatic | 0.42 | 0.07 | 0.30 - 0.55 | <0.001 | **<0.001** | 0.46 |
|  |  | CBCL - Social | 0.49 | 0.05 | 0.39 - 0.59 | <0.001 | **<0.001** | 1.02 |
|  |  | CBCL - Thought | 0.59 | 0.06 | 0.47 - 0.72 | <0.001 | **<0.001** | 0.92 |
|  |  | CBCL - Attention | 0.53 | 0.07 | 0.39 - 0.66 | <0.001 | **<0.001** | 0.66 |
|  |  | CBCL - Rule Breaking | 0.35 | 0.05 | 0.25 - 0.46 | <0.001 | **<0.001** | 0.48 |
|  |  | CBCL - Aggressive | 0.48 | 0.06 | 0.36 - 0.59 | <0.001 | **<0.001** | 0.69 |
|  |  | PQB - Distress Score | 0.38 | 0.11 | 0.16 - 0.59 | <0.001 | 0.002 | 0.12 |
|  |  |  |  |  |  |  |  |  |
|  | SCZ PRS | NIH Total Cognitive Score | -0.43 | 0.17 | -0.77 - -0.10 | 0.012 | **0.023** | 0.07 |
|  |  | CBCL - Total | 0.08 | 0.12 | -0.15 - 0.32 | 0.502 | 0.649 | 0.00 |
|  |  | CBCL - Anxious Depressed | 0.08 | 0.06 | -0.05 - 0.20 | 0.230 | 0.338 | 0.02 |
|  |  | CBCL - Withdrawn | 0.01 | 0.06 | -0.11 - 0.14 | 0.819 | 0.868 | 0.00 |
|  |  | CBCL - Somatic | -0.09 | 0.06 | -0.21 - 0.04 | 0.173 | 0.272 | 0.02 |
|  |  | CBCL - Social | 0.01 | 0.05 | -0.09 - 0.11 | 0.829 | 0.868 | 0.00 |
|  |  | CBCL - Thought | 0.07 | 0.06 | -0.06 - 0.19 | 0.293 | 0.402 | 0.01 |
|  |  | CBCL - Attention | 0.00 | 0.07 | -0.13 - 0.13 | 0.989 | 0.989 | 0.00 |
|  |  | CBCL - Rule Breaking | 0.07 | 0.05 | -0.03 - 0.18 | 0.163 | 0.272 | 0.02 |
|  |  | CBCL - Aggressive | 0.03 | 0.06 | -0.09 - 0.14 | 0.659 | 0.763 | 0.00 |
|  |  | PQB - Distress Score | 0.15 | 0.11 | -0.07 - 0.37 | 0.171 | 0.272 | 0.02 |
|  |  |  |  |  |  |  |  |  |
| European | Family History | NIH Total Cognitive Score | -0.42 | 0.28 | -0.98 - 0.13 | 0.132 | 0.161 | 0.04 |
|  |  | CBCL - Total | 0.97 | 0.19 | 0.60 - 1.33 | <0.001 | **<0.001** | 0.48 |
|  |  | CBCL - Anxious Depressed | 0.27 | 0.10 | 0.07 - 0.48 | 0.009 | **0.017** | 0.12 |
|  |  | CBCL - Withdrawn | 0.25 | 0.09 | 0.07 - 0.44 | 0.008 | **0.015** | 0.13 |
|  |  | CBCL - Somatic | 0.42 | 0.10 | 0.22 - 0.62 | <0.001 | **<0.001** | 0.29 |
|  |  | CBCL - Social | 0.40 | 0.08 | 0.25 - 0.55 | <0.001 | **<0.001** | 0.47 |
|  |  | CBCL - Thought | 0.65 | 0.10 | 0.45 - 0.85 | <0.001 | **<0.001** | 0.71 |
|  |  | CBCL - Attention | 0.61 | 0.10 | 0.41 - 0.81 | <0.001 | **<0.001** | 0.63 |
|  |  | CBCL - Rule Breaking | 0.34 | 0.08 | 0.19 - 0.49 | <0.001 | **<0.001** | 0.35 |
|  |  | CBCL - Aggressive | 0.39 | 0.09 | 0.22 - 0.57 | <0.001 | **<0.001** | 0.34 |
|  |  | PQB - Distress Score | 0.45 | 0.16 | 0.15 - 0.76 | 0.004 | **0.008** | 0.15 |
|  |  |  |  |  |  |  |  |  |
|  | SCZ PRS | NIH Total Cognitive Score | -0.77 | 0.22 | -1.20 - -0.33 | 0.001 | **0.001** | 0.22 |
|  |  | CBCL - Total | 0.33 | 0.15 | 0.05 - 0.62 | 0.023 | **0.034** | 0.09 |
|  |  | CBCL - Anxious Depressed | 0.21 | 0.08 | 0.05 - 0.37 | 0.012 | **0.020** | 0.11 |
|  |  | CBCL - Withdrawn | 0.09 | 0.08 | -0.06 - 0.24 | 0.220 | 0.255 | 0.03 |
|  |  | CBCL - Somatic | -0.08 | 0.08 | -0.24 - 0.08 | 0.349 | 0.378 | 0.02 |
|  |  | CBCL - Social | 0.03 | 0.06 | -0.09 - 0.15 | 0.668 | 0.668 | 0.00 |
|  |  | CBCL - Thought | 0.14 | 0.08 | -0.02 - 0.30 | 0.088 | 0.114 | 0.05 |
|  |  | CBCL - Attention | 0.07 | 0.08 | -0.09 - 0.23 | 0.361 | 0.378 | 0.01 |
|  |  | CBCL - Rule Breaking | 0.21 | 0.06 | 0.09 - 0.33 | 0.000 | **0.001** | 0.22 |
|  |  | CBCL - Aggressive | 0.17 | 0.07 | 0.03 - 0.31 | 0.020 | **0.032** | 0.10 |
|  |  | PQB - Distress Score | 0.21 | 0.12 | -0.03 - 0.45 | 0.086 | 0.114 | 0.05 |
|  |  |  |  |  |  |  |  |  |
| African | Family History | NIH Total Cognitive Score | -0.03 | 0.27 | -0.55 - 0.49 | 0.900 | 0.943 | 0.00 |
|  |  | CBCL - Total | 1.25 | 0.20 | 0.86 - 1.65 | <0.001 | **<0.001** | 1.82 |
|  |  | CBCL - Anxious Depressed | 0.46 | 0.09 | 0.29 - 0.64 | <0.001 | **<0.001** | 1.30 |
|  |  | CBCL - Withdrawn | 0.52 | 0.10 | 0.32 - 0.72 | <0.001 | **<0.001** | 1.25 |
|  |  | CBCL - Somatic | 0.41 | 0.10 | 0.21 - 0.60 | <0.001 | **<0.001** | 0.81 |
|  |  | CBCL - Social | 0.57 | 0.08 | 0.41 - 0.74 | <0.001 | **<0.001** | 2.18 |
|  |  | CBCL - Thought | 0.60 | 0.10 | 0.40 - 0.80 | <0.001 | **<0.001** | 1.66 |
|  |  | CBCL - Attention | 0.45 | 0.11 | 0.23 - 0.67 | <0.001 | **<0.001** | 0.76 |
|  |  | CBCL - Rule Breaking | 0.35 | 0.10 | 0.15 - 0.55 | <0.001 | **<0.001** | 0.58 |
|  |  | CBCL - Aggressive | 0.52 | 0.10 | 0.32 - 0.72 | <0.001 | **<0.001** | 1.20 |
|  |  | PQB - Distress Score | 0.26 | 0.20 | -0.14 - 0.65 | 0.202 | 0.444 | 0.08 |
|  |  |  |  |  |  |  |  |  |
|  | SCZ PRS | NIH Total Cognitive Score | 0.34 | 0.42 | -0.48 - 1.16 | 0.417 | 0.684 | 0.03 |
|  |  | CBCL - Total | -0.17 | 0.33 | -0.82 - 0.48 | 0.614 | 0.747 | 0.01 |
|  |  | CBCL - Anxious Depressed | -0.06 | 0.15 | -0.35 - 0.23 | 0.679 | 0.747 | 0.01 |
|  |  | CBCL - Withdrawn | -0.19 | 0.17 | -0.52 - 0.14 | 0.267 | 0.530 | 0.06 |
|  |  | CBCL - Somatic | -0.07 | 0.16 | -0.38 - 0.25 | 0.678 | 0.747 | 0.01 |
|  |  | CBCL - Social | -0.01 | 0.14 | -0.28 - 0.27 | 0.962 | 0.962 | 0.00 |
|  |  | CBCL - Thought | -0.07 | 0.17 | -0.40 - 0.26 | 0.675 | 0.747 | 0.01 |
|  |  | CBCL - Attention | 0.13 | 0.18 | -0.23 - 0.50 | 0.466 | 0.684 | 0.03 |
|  |  | CBCL - Rule Breaking | -0.17 | 0.16 | -0.50 - 0.15 | 0.289 | 0.530 | 0.05 |
|  |  | CBCL - Aggressive | -0.13 | 0.17 | -0.46 - 0.20 | 0.446 | 0.684 | 0.03 |
|  |  | PQB - Distress Score | 0.22 | 0.34 | -0.44 - 0.87 | 0.521 | 0.716 | 0.02 |
|  |  |  |  |  |  |  |  |  |
| American Admixed | Family History | NIH Total Cognitive Score | 0.26 | 0.43 | -0.58 - 1.11 | 0.540 | 0.626 | 0.03 |
|  |  | CBCL - Total | 0.90 | 0.30 | 0.31 - 1.49 | 0.003 | **0.010** | 0.61 |
|  |  | CBCL - Anxious Depressed | 0.48 | 0.16 | 0.17 - 0.78 | 0.002 | **0.010** | 0.63 |
|  |  | CBCL - Withdrawn | 0.57 | 0.17 | 0.24 - 0.90 | 0.001 | **0.005** | 0.79 |
|  |  | CBCL - Somatic | 0.43 | 0.17 | 0.09 - 0.76 | 0.012 | **0.029** | 0.43 |
|  |  | CBCL - Social | 0.44 | 0.13 | 0.19 - 0.69 | 0.001 | **0.005** | 0.79 |
|  |  | CBCL - Thought | 0.42 | 0.15 | 0.12 - 0.72 | 0.005 | **0.016** | 0.53 |
|  |  | CBCL - Attention | 0.52 | 0.16 | 0.20 - 0.85 | 0.001 | **0.008** | 0.70 |
|  |  | CBCL - Rule Breaking | 0.34 | 0.12 | 0.10 - 0.58 | 0.006 | **0.016** | 0.52 |
|  |  | CBCL - Aggressive | 0.51 | 0.14 | 0.23 - 0.79 | <0.001 | **0.005** | 0.88 |
|  |  | PQB - Distress Score | 0.52 | 0.31 | -0.08 - 1.13 | 0.092 | 0.201 | 0.19 |
|  |  |  |  |  |  |  |  |  |
|  | SCZ PRS | NIH Total Cognitive Score | 0.10 | 0.45 | -0.77 - 0.98 | 0.817 | 0.889 | 0.00 |
|  |  | CBCL - Total | 0.33 | 0.31 | -0.28 - 0.95 | 0.286 | 0.420 | 0.08 |
|  |  | CBCL - Anxious Depressed | 0.18 | 0.16 | -0.14 - 0.50 | 0.274 | 0.420 | 0.08 |
|  |  | CBCL - Withdrawn | 0.24 | 0.18 | -0.10 - 0.59 | 0.167 | 0.306 | 0.13 |
|  |  | CBCL - Somatic | 0.14 | 0.18 | -0.20 - 0.49 | 0.424 | 0.548 | 0.04 |
|  |  | CBCL - Social | 0.22 | 0.13 | -0.04 - 0.48 | 0.102 | 0.204 | 0.18 |
|  |  | CBCL - Thought | 0.15 | 0.16 | -0.15 - 0.46 | 0.327 | 0.449 | 0.07 |
|  |  | CBCL - Attention | -0.03 | 0.17 | -0.36 - 0.30 | 0.848 | 0.889 | 0.00 |
|  |  | CBCL - Rule Breaking | 0.16 | 0.13 | -0.09 - 0.41 | 0.201 | 0.340 | 0.11 |
|  |  | CBCL - Aggressive | 0.09 | 0.15 | -0.20 - 0.38 | 0.530 | 0.626 | 0.03 |
|  |  | PQB - Distress Score | 0.02 | 0.32 | -0.61 - 0.65 | 0.946 | 0.946 | 0.00 |

Table S10. Independent associations of schizophrenia polygenic risk scores (SCZ-PRS) and family history of psychosis (SCZ-FH) with diagnostic phenotypes derived from the Kiddie Schedule for Affective Disorders and Schizophrenia (KSADS) or Child Behavior Checklist (CBCL), including family conflict as a covariate. FDR P refers to the false discovery rate–corrected p value; PVE refers to the percentage of variance explained.

| **Ancestry** | **Genetic Risk Measure** | **Precursor** | **OR** | **SE** | **95% CI** | **Nominal P** | **FDR P** | **PVE (%)** |
| --- | --- | --- | --- | --- | --- | --- | --- | --- |
| All | Family History | KSADS - ADHD | 1.13 | 0.02 | 1.08 - 1.19 | <0.001 | **<0.001** | 0.35 |
|  |  | CBCL - ADHD | 1.20 | 0.03 | 1.13 - 1.27 | <0.001 | **<0.001** | 0.61 |
|  |  | KSADS - Anxiety Disorders | 1.22 | 0.03 | 1.16 - 1.28 | <0.001 | **<0.001** | 0.91 |
|  |  | KSADS - Anxiety disorders w/ PTSD | 1.23 | 0.02 | 1.17 - 1.29 | <0.001 | **<0.001** | 1.01 |
|  |  | KSADS - Depressive Disorders | 1.13 | 0.03 | 1.06 - 1.21 | <0.001 | **<0.001** | 0.20 |
|  |  | KSADS - Conduct Disorder | 1.10 | 0.03 | 1.04 - 1.16 | <0.001 | **<0.001** | 0.17 |
|  |  |  |  |  |  |  |  |  |
|  | SCZ PRS | KSADS - ADHD | 1.03 | 0.03 | 0.98 - 1.09 | 0.280 | 0.433 | 0.01 |
|  |  | CBCL - ADHD | 0.96 | 0.04 | 0.88 - 1.04 | 0.289 | 0.433 | 0.02 |
|  |  | KSADS - Anxiety Disorders | 1.03 | 0.03 | 0.97 - 1.09 | 0.352 | 0.453 | 0.01 |
|  |  | KSADS - Anxiety disorders w/ PTSD | 1.03 | 0.03 | 0.97 - 1.09 | 0.378 | 0.453 | 0.01 |
|  |  | KSADS - Depressive Disorders | 1.03 | 0.05 | 0.94 - 1.13 | 0.492 | 0.536 | 0.01 |
|  |  | KSADS - Conduct Disorder | 1.01 | 0.03 | 0.95 - 1.07 | 0.681 | 0.681 | 0.00 |
|  |  |  |  |  |  |  |  |  |
| European | Family History | KSADS - ADHD | 1.14 | 0.04 | 1.05 - 1.23 | 0.001 | **0.003** | 0.22 |
|  |  | CBCL - ADHD | 1.27 | 0.05 | 1.15 - 1.40 | <0.001 | **<0.001** | 0.58 |
|  |  | KSADS - Anxiety Disorders | 1.19 | 0.04 | 1.10 - 1.29 | <0.001 | **<0.001** | 0.43 |
|  |  | KSADS - Anxiety disorders w/ PTSD | 1.19 | 0.04 | 1.10 - 1.29 | <0.001 | **<0.001** | 0.44 |
|  |  | KSADS - Depressive Disorders | 1.14 | 0.07 | 1.00 - 1.30 | 0.059 | 0.118 | 0.09 |
|  |  | KSADS - Conduct Disorder | 1.04 | 0.04 | 0.95 - 1.14 | 0.354 | 0.386 | 0.02 |
|  |  |  |  |  |  |  |  |  |
|  | SCZ PRS | KSADS - ADHD | 1.08 | 0.03 | 1.01 - 1.16 | 0.022 | 0.053 | 0.11 |
|  |  | CBCL - ADHD | 1.02 | 0.06 | 0.92 - 1.14 | 0.699 | 0.699 | 0.00 |
|  |  | KSADS - Anxiety Disorders | 1.06 | 0.04 | 0.98 - 1.14 | 0.123 | 0.149 | 0.05 |
|  |  | KSADS - Anxiety disorders w/ PTSD | 1.07 | 0.04 | 0.99 - 1.15 | 0.083 | 0.124 | 0.07 |
|  |  | KSADS - Depressive Disorders | 1.11 | 0.07 | 0.97 - 1.27 | 0.124 | 0.149 | 0.06 |
|  |  | KSADS - Conduct Disorder | 1.07 | 0.04 | 0.99 - 1.15 | 0.081 | 0.124 | 0.06 |
|  |  |  |  |  |  |  |  |  |
| African | Family History | KSADS - ADHD | 1.11 | 0.04 | 1.03 - 1.19 | 0.004 | **0.009** | 0.46 |
|  |  | CBCL - ADHD | 1.15 | 0.04 | 1.06 - 1.24 | 0.001 | **0.004** | 0.74 |
|  |  | KSADS - Anxiety Disorders | 1.22 | 0.04 | 1.13 - 1.32 | <0.001 | **<0.001** | 1.99 |
|  |  | KSADS - Anxiety disorders w/ PTSD | 1.25 | 0.04 | 1.16 - 1.34 | <0.001 | **<0.001** | 2.48 |
|  |  | KSADS - Depressive Disorders | 1.14 | 0.04 | 1.05 - 1.24 | 0.003 | **0.006** | 0.60 |
|  |  | KSADS - Conduct Disorder | 1.12 | 0.04 | 1.04 - 1.21 | 0.002 | **0.006** | 0.58 |
|  |  |  |  |  |  |  |  |  |
|  | SCZ PRS | KSADS - ADHD | 1.01 | 0.07 | 0.89 - 1.15 | 0.828 | 0.909 | 0.00 |
|  |  | CBCL - ADHD | 0.98 | 0.09 | 0.82 - 1.17 | 0.838 | 0.909 | 0.00 |
|  |  | KSADS - Anxiety Disorders | 1.03 | 0.08 | 0.88 - 1.21 | 0.683 | 0.909 | 0.01 |
|  |  | KSADS - Anxiety disorders w/ PTSD | 1.01 | 0.08 | 0.86 - 1.18 | 0.909 | 0.909 | 0.00 |
|  |  | KSADS - Depressive Disorders | 0.90 | 0.10 | 0.74 - 1.09 | 0.274 | 0.470 | 0.07 |
|  |  | KSADS - Conduct Disorder | 1.02 | 0.08 | 0.88 - 1.19 | 0.752 | 0.909 | 0.01 |
|  |  |  |  |  |  |  |  |  |
| American Admixed | Family History | KSADS - ADHD | 1.20 | 0.06 | 1.07 - 1.35 | 0.002 | **0.009** | 0.77 |
|  |  | CBCL - ADHD | 1.23 | 0.08 | 1.06 - 1.43 | 0.006 | **0.018** | 0.70 |
|  |  | KSADS - Anxiety Disorders | 1.25 | 0.07 | 1.10 - 1.42 | 0.001 | **0.006** | 1.14 |
|  |  | KSADS - Anxiety disorders w/ PTSD | 1.24 | 0.07 | 1.09 - 1.40 | 0.001 | **0.007** | 1.00 |
|  |  | KSADS - Depressive Disorders | 1.13 | 0.09 | 0.95 - 1.34 | 0.172 | 0.295 | 0.14 |
|  |  | KSADS - Conduct Disorder | 1.16 | 0.07 | 1.01 - 1.34 | 0.035 | 0.070 | 0.37 |
|  |  |  |  |  |  |  |  |  |
|  | SCZ PRS | KSADS - ADHD | 0.95 | 0.07 | 0.82 - 1.10 | 0.509 | 0.678 | 0.03 |
|  |  | CBCL - ADHD | 0.92 | 0.11 | 0.74 - 1.13 | 0.419 | 0.628 | 0.06 |
|  |  | KSADS - Anxiety Disorders | 1.05 | 0.09 | 0.88 - 1.26 | 0.595 | 0.714 | 0.02 |
|  |  | KSADS - Anxiety disorders w/ PTSD | 1.02 | 0.09 | 0.86 - 1.22 | 0.812 | 0.812 | 0.00 |
|  |  | KSADS - Depressive Disorders | 1.36 | 0.12 | 1.08 - 1.72 | 0.009 | **0.023** | 0.50 |
|  |  | KSADS - Conduct Disorder | 0.96 | 0.10 | 0.80 - 1.16 | 0.660 | 0.720 | 0.02 |

Table S11. Independent associations of schizophrenia polygenic risk scores (SCZ-PRS) and family history of psychosis (SCZ-FH) with dimensional, total cognitive score derived from the NIH-Toolbox, Child Behavior Checklist (CBCL) scores, and prodromal questionnaire (PQB) scores, including parental monitoring as a covariate. FDR P refers to the false discovery rate–corrected p value; PVE refers to the percentage of variance explained.

| **Ancestry** | **Genetic Risk Measure** | **Precursor** | **B** | **SE** | **95% CI** | **Nominal P** | **FDR P** | **PVE (%)** |
| --- | --- | --- | --- | --- | --- | --- | --- | --- |
| All | Family History | NIH Total Cognitive Score | -0.09 | 0.18 | -0.44 - 0.26 | 0.612 | 0.792 | 0.00 |
|  |  | CBCL - Total | 1.09 | 0.12 | 0.85 - 1.33 | <0.001 | **<0.001** | 0.88 |
|  |  | CBCL - Anxious Depressed | 0.39 | 0.06 | 0.27 - 0.51 | <0.001 | **<0.001** | 0.41 |
|  |  | CBCL - Withdrawn | 0.41 | 0.06 | 0.29 - 0.53 | <0.001 | **<0.001** | 0.48 |
|  |  | CBCL - Somatic | 0.42 | 0.07 | 0.29 - 0.55 | <0.001 | **<0.001** | 0.45 |
|  |  | CBCL - Social | 0.48 | 0.05 | 0.38 - 0.58 | <0.001 | **<0.001** | 0.99 |
|  |  | CBCL - Thought | 0.58 | 0.06 | 0.46 - 0.71 | <0.001 | **<0.001** | 0.89 |
|  |  | CBCL - Attention | 0.51 | 0.07 | 0.38 - 0.64 | <0.001 | **<0.001** | 0.63 |
|  |  | CBCL - Rule Breaking | 0.35 | 0.05 | 0.25 - 0.45 | <0.001 | **<0.001** | 0.47 |
|  |  | CBCL - Aggressive | 0.47 | 0.06 | 0.36 - 0.59 | <0.001 | **<0.001** | 0.68 |
|  |  | PQB - Distress Score | 0.37 | 0.11 | 0.15 - 0.58 | <0.001 | **0.002** | 0.12 |
|  |  |  |  |  |  |  |  |  |
|  | SCZ PRS | NIH Total Cognitive Score | -0.41 | 0.17 | -0.74 - -0.07 | 0.018 | **0.035** | 0.06 |
|  |  | CBCL - Total | 0.05 | 0.12 | -0.18 - 0.29 | 0.660 | 0.806 | 0.00 |
|  |  | CBCL - Anxious Depressed | 0.07 | 0.06 | -0.06 - 0.19 | 0.290 | 0.426 | 0.01 |
|  |  | CBCL - Withdrawn | 0.00 | 0.06 | -0.13 - 0.12 | 0.936 | 0.981 | 0.00 |
|  |  | CBCL - Somatic | -0.09 | 0.06 | -0.22 - 0.03 | 0.155 | 0.284 | 0.02 |
|  |  | CBCL - Social | 0.00 | 0.05 | -0.10 - 0.10 | 0.995 | 0.995 | 0.00 |
|  |  | CBCL - Thought | 0.05 | 0.06 | -0.07 - 0.18 | 0.395 | 0.543 | 0.01 |
|  |  | CBCL - Attention | -0.02 | 0.07 | -0.15 - 0.11 | 0.797 | 0.879 | 0.00 |
|  |  | CBCL - Rule Breaking | 0.06 | 0.05 | -0.04 - 0.17 | 0.228 | 0.385 | 0.02 |
|  |  | CBCL - Aggressive | 0.02 | 0.06 | -0.10 - 0.13 | 0.799 | 0.879 | 0.00 |
|  |  | PQB - Distress Score | 0.12 | 0.11 | -0.10 - 0.33 | 0.280 | 0.426 | 0.01 |
|  |  |  |  |  |  |  |  |  |
| European | Family History | NIH Total Cognitive Score | -0.35 | 0.28 | -0.90 - 0.20 | 0.214 | 0.262 | 0.03 |
|  |  | CBCL - Total | 0.91 | 0.19 | 0.55 - 1.28 | <0.001 | **<0.001** | 0.43 |
|  |  | CBCL - Anxious Depressed | 0.25 | 0.10 | 0.05 - 0.46 | 0.015 | **0.031** | 0.10 |
|  |  | CBCL - Withdrawn | 0.22 | 0.09 | 0.03 - 0.40 | 0.022 | **0.037** | 0.09 |
|  |  | CBCL - Somatic | 0.41 | 0.10 | 0.21 - 0.61 | <0.001 | **<0.001** | 0.29 |
|  |  | CBCL - Social | 0.37 | 0.08 | 0.22 - 0.52 | <0.001 | **<0.001** | 0.41 |
|  |  | CBCL - Thought | 0.62 | 0.10 | 0.42 - 0.82 | <0.001 | **<0.001** | 0.65 |
|  |  | CBCL - Attention | 0.57 | 0.10 | 0.37 - 0.77 | <0.001 | **<0.001** | 0.56 |
|  |  | CBCL - Rule Breaking | 0.32 | 0.08 | 0.17 - 0.47 | <0.001 | **<0.001** | 0.31 |
|  |  | CBCL - Aggressive | 0.37 | 0.09 | 0.19 - 0.55 | <0.001 | **<0.001** | 0.30 |
|  |  | PQB - Distress Score | 0.38 | 0.15 | 0.08 - 0.68 | 0.013 | **0.028** | 0.11 |
|  |  |  |  |  |  |  |  |  |
|  | SCZ PRS | NIH Total Cognitive Score | -0.74 | 0.22 | -1.17 - -0.30 | <0.001 | **0.002** | 0.20 |
|  |  | CBCL - Total | 0.31 | 0.15 | 0.02 - 0.59 | 0.036 | 0.053 | 0.08 |
|  |  | CBCL - Anxious Depressed | 0.20 | 0.08 | 0.04 - 0.36 | 0.017 | **0.031** | 0.10 |
|  |  | CBCL - Withdrawn | 0.07 | 0.07 | -0.08 - 0.22 | 0.341 | 0.375 | 0.02 |
|  |  | CBCL - Somatic | -0.08 | 0.08 | -0.24 - 0.08 | 0.324 | 0.375 | 0.02 |
|  |  | CBCL - Social | 0.01 | 0.06 | -0.11 - 0.13 | 0.832 | 0.832 | 0.00 |
|  |  | CBCL - Thought | 0.12 | 0.08 | -0.04 - 0.28 | 0.132 | 0.180 | 0.04 |
|  |  | CBCL - Attention | 0.05 | 0.08 | -0.11 - 0.21 | 0.513 | 0.538 | 0.01 |
|  |  | CBCL - Rule Breaking | 0.20 | 0.06 | 0.08 - 0.32 | <0.001 | **0.002** | 0.20 |
|  |  | CBCL - Aggressive | 0.15 | 0.07 | 0.01 - 0.29 | 0.033 | 0.053 | 0.08 |
|  |  | PQB - Distress Score | 0.18 | 0.12 | -0.06 - 0.42 | 0.139 | 0.180 | 0.04 |
|  |  |  |  |  |  |  |  |  |
| African | Family History | NIH Total Cognitive Score | -0.03 | 0.27 | -0.55 - 0.49 | 0.920 | 0.920 | 0.00 |
|  |  | CBCL - Total | 1.25 | 0.20 | 0.86 - 1.65 | <0.001 | **<0.001** | 1.83 |
|  |  | CBCL - Anxious Depressed | 0.46 | 0.09 | 0.29 - 0.63 | <0.001 | **<0.001** | 1.28 |
|  |  | CBCL - Withdrawn | 0.51 | 0.10 | 0.31 - 0.71 | <0.001 | **<0.001** | 1.23 |
|  |  | CBCL - Somatic | 0.41 | 0.10 | 0.21 - 0.60 | <0.001 | **<0.001** | 0.81 |
|  |  | CBCL - Social | 0.57 | 0.08 | 0.41 - 0.73 | <0.001 | **<0.001** | 2.18 |
|  |  | CBCL - Thought | 0.60 | 0.10 | 0.40 - 0.80 | <0.001 | **<0.001** | 1.68 |
|  |  | CBCL - Attention | 0.45 | 0.11 | 0.23 - 0.67 | <0.001 | **<0.001** | 0.76 |
|  |  | CBCL - Rule Breaking | 0.36 | 0.10 | 0.16 - 0.56 | <0.001 | **<0.001** | 0.61 |
|  |  | CBCL - Aggressive | 0.53 | 0.10 | 0.32 - 0.73 | <0.001 | **<0.001** | 1.23 |
|  |  | PQB - Distress Score | 0.31 | 0.20 | -0.09 - 0.70 | 0.127 | 0.279 | 0.11 |
|  |  |  |  |  |  |  |  |  |
|  | SCZ PRS | NIH Total Cognitive Score | 0.36 | 0.42 | -0.46 - 1.18 | 0.386 | 0.632 | 0.04 |
|  |  | CBCL - Total | -0.21 | 0.33 | -0.86 - 0.43 | 0.519 | 0.738 | 0.02 |
|  |  | CBCL - Anxious Depressed | -0.07 | 0.15 | -0.36 - 0.22 | 0.640 | 0.738 | 0.01 |
|  |  | CBCL - Withdrawn | -0.21 | 0.17 | -0.53 - 0.12 | 0.217 | 0.435 | 0.07 |
|  |  | CBCL - Somatic | -0.07 | 0.16 | -0.39 - 0.24 | 0.648 | 0.738 | 0.01 |
|  |  | CBCL - Social | -0.02 | 0.14 | -0.30 - 0.25 | 0.865 | 0.907 | 0.00 |
|  |  | CBCL - Thought | -0.08 | 0.17 | -0.41 - 0.24 | 0.620 | 0.738 | 0.01 |
|  |  | CBCL - Attention | 0.11 | 0.18 | -0.25 - 0.47 | 0.538 | 0.738 | 0.02 |
|  |  | CBCL - Rule Breaking | -0.19 | 0.17 | -0.51 - 0.13 | 0.249 | 0.457 | 0.06 |
|  |  | CBCL - Aggressive | -0.14 | 0.17 | -0.48 - 0.19 | 0.402 | 0.632 | 0.03 |
|  |  | PQB - Distress Score | 0.14 | 0.33 | -0.51 - 0.80 | 0.671 | 0.738 | 0.01 |
|  |  |  |  |  |  |  |  |  |
| American Admixed | Family History | NIH Total Cognitive Score | 0.29 | 0.43 | -0.56 - 1.14 | 0.501 | 0.612 | 0.03 |
|  |  | CBCL - Total | 0.90 | 0.30 | 0.31 - 1.48 | 0.003 | **0.011** | 0.62 |
|  |  | CBCL - Anxious Depressed | 0.47 | 0.16 | 0.16 - 0.78 | 0.003 | **0.011** | 0.61 |
|  |  | CBCL - Withdrawn | 0.57 | 0.17 | 0.24 - 0.90 | 0.001 | **0.005** | 0.79 |
|  |  | CBCL - Somatic | 0.43 | 0.17 | 0.10 - 0.76 | 0.012 | **0.029** | 0.43 |
|  |  | CBCL - Social | 0.44 | 0.13 | 0.18 - 0.69 | 0.001 | **0.005** | 0.78 |
|  |  | CBCL - Thought | 0.42 | 0.15 | 0.12 - 0.71 | 0.005 | **0.015** | 0.53 |
|  |  | CBCL - Attention | 0.52 | 0.16 | 0.20 - 0.84 | 0.001 | **0.008** | 0.69 |
|  |  | CBCL - Rule Breaking | 0.34 | 0.12 | 0.10 - 0.58 | 0.006 | **0.015** | 0.53 |
|  |  | CBCL - Aggressive | 0.51 | 0.14 | 0.24 - 0.79 | <0.001 | **0.005** | 0.89 |
|  |  | PQB - Distress Score | 0.56 | 0.31 | -0.04 - 1.17 | 0.068 | 0.150 | 0.23 |
|  |  |  |  |  |  |  |  |  |
|  | SCZ PRS | NIH Total Cognitive Score | 0.13 | 0.45 | -0.74 - 1.01 | 0.765 | 0.805 | 0.01 |
|  |  | CBCL - Total | 0.29 | 0.31 | -0.32 - 0.90 | 0.353 | 0.518 | 0.06 |
|  |  | CBCL - Anxious Depressed | 0.16 | 0.16 | -0.16 - 0.48 | 0.334 | 0.518 | 0.06 |
|  |  | CBCL - Withdrawn | 0.21 | 0.17 | -0.13 - 0.55 | 0.236 | 0.399 | 0.10 |
|  |  | CBCL - Somatic | 0.14 | 0.18 | -0.21 - 0.48 | 0.443 | 0.574 | 0.04 |
|  |  | CBCL - Social | 0.21 | 0.13 | -0.05 - 0.47 | 0.122 | 0.243 | 0.16 |
|  |  | CBCL - Thought | 0.14 | 0.16 | -0.17 - 0.44 | 0.380 | 0.522 | 0.05 |
|  |  | CBCL - Attention | -0.05 | 0.17 | -0.38 - 0.28 | 0.769 | 0.805 | 0.01 |
|  |  | CBCL - Rule Breaking | 0.16 | 0.13 | -0.09 - 0.41 | 0.223 | 0.399 | 0.10 |
|  |  | CBCL - Aggressive | 0.09 | 0.15 | -0.21 - 0.38 | 0.562 | 0.650 | 0.02 |
|  |  | PQB - Distress Score | -0.02 | 0.32 | -0.65 - 0.61 | 0.957 | 0.957 | 0.00 |

Table S12. Independent associations of schizophrenia polygenic risk scores (SCZ-PRS) and family history of psychosis (SCZ-FH) with diagnostic phenotypes derived from the Kiddie Schedule for Affective Disorders and Schizophrenia (KSADS) or Child Behavior Checklist (CBCL), including parental monitoring as a covariate. FDR P refers to the false discovery rate–corrected p value; PVE refers to the percentage of variance explained.

| **Ancestry** | **Genetic Risk Measure** | **Precursor** | **OR** | **SE** | **95% CI** | **Nominal P** | **FDR P** | **PVE (%)** |
| --- | --- | --- | --- | --- | --- | --- | --- | --- |
| All | Family History | KSADS - ADHD | 1.13 | 0.02 | 1.08 - 1.18 | <0.001 | **<0.001** | 0.32 |
|  |  | CBCL - ADHD | 1.19 | 0.03 | 1.13 - 1.26 | <0.001 | **<0.001** | 0.57 |
|  |  | KSADS - Anxiety Disorders | 1.21 | 0.03 | 1.15 - 1.27 | <0.001 | **<0.001** | 0.87 |
|  |  | KSADS - Anxiety disorders w/ PTSD | 1.22 | 0.02 | 1.16 - 1.28 | <0.001 | **<0.001** | 0.97 |
|  |  | KSADS - Depressive Disorders | 1.14 | 0.03 | 1.07 - 1.21 | <0.001 | **<0.001** | 0.21 |
|  |  | KSADS - Conduct Disorder | 1.10 | 0.03 | 1.04 - 1.16 | <0.001 | **<0.001** | 0.16 |
|  |  |  |  |  |  |  |  |  |
|  | SCZ PRS | KSADS - ADHD | 1.02 | 0.03 | 0.97 - 1.08 | 0.407 | 0.496 | 0.01 |
|  |  | CBCL - ADHD | 0.95 | 0.04 | 0.88 - 1.03 | 0.214 | 0.366 | 0.02 |
|  |  | KSADS - Anxiety Disorders | 1.03 | 0.03 | 0.97 - 1.09 | 0.388 | 0.496 | 0.01 |
|  |  | KSADS - Anxiety disorders w/ PTSD | 1.02 | 0.03 | 0.97 - 1.09 | 0.413 | 0.496 | 0.01 |
|  |  | KSADS - Depressive Disorders | 1.02 | 0.05 | 0.93 - 1.12 | 0.638 | 0.696 | 0.00 |
|  |  | KSADS - Conduct Disorder | 1.01 | 0.03 | 0.95 - 1.07 | 0.807 | 0.807 | 0.00 |
|  |  |  |  |  |  |  |  |  |
| European | Family History | KSADS - ADHD | 1.12 | 0.04 | 1.04 - 1.21 | 0.004 | **0.013** | 0.17 |
|  |  | CBCL - ADHD | 1.25 | 0.05 | 1.13 - 1.38 | <0.001 | **<0.001** | 0.48 |
|  |  | KSADS - Anxiety Disorders | 1.18 | 0.04 | 1.09 - 1.28 | <0.001 | **<0.001** | 0.40 |
|  |  | KSADS - Anxiety disorders w/ PTSD | 1.18 | 0.04 | 1.10 - 1.28 | <0.001 | **<0.001** | 0.41 |
|  |  | KSADS - Depressive Disorders | 1.12 | 0.07 | 0.98 - 1.28 | 0.094 | 0.162 | 0.07 |
|  |  | KSADS - Conduct Disorder | 1.03 | 0.05 | 0.95 - 1.13 | 0.462 | 0.504 | 0.01 |
|  |  |  |  |  |  |  |  |  |
|  | SCZ PRS | KSADS - ADHD | 1.07 | 0.03 | 1.00 - 1.15 | 0.044 | 0.105 | 0.08 |
|  |  | CBCL - ADHD | 1.01 | 0.06 | 0.91 - 1.13 | 0.833 | 0.833 | 0.00 |
|  |  | KSADS - Anxiety Disorders | 1.06 | 0.04 | 0.98 - 1.14 | 0.140 | 0.186 | 0.05 |
|  |  | KSADS - Anxiety disorders w/ PTSD | 1.06 | 0.04 | 0.99 - 1.14 | 0.094 | 0.162 | 0.06 |
|  |  | KSADS - Depressive Disorders | 1.10 | 0.07 | 0.96 - 1.25 | 0.166 | 0.199 | 0.05 |
|  |  | KSADS - Conduct Disorder | 1.06 | 0.04 | 0.99 - 1.14 | 0.110 | 0.165 | 0.05 |
|  |  |  |  |  |  |  |  |  |
| African | Family History | KSADS - ADHD | 1.11 | 0.04 | 1.03 - 1.19 | 0.003 | **0.007** | 0.49 |
|  |  | CBCL - ADHD | 1.15 | 0.04 | 1.06 - 1.24 | 0.001 | **0.003** | 0.74 |
|  |  | KSADS - Anxiety Disorders | 1.22 | 0.04 | 1.14 - 1.32 | <0.001 | **<0.001** | 2.01 |
|  |  | KSADS - Anxiety disorders w/ PTSD | 1.25 | 0.04 | 1.16 - 1.35 | <0.001 | **<0.001** | 2.53 |
|  |  | KSADS - Depressive Disorders | 1.15 | 0.04 | 1.06 - 1.26 | 0.001 | **0.003** | 0.69 |
|  |  | KSADS - Conduct Disorder | 1.13 | 0.04 | 1.05 - 1.21 | 0.001 | **0.003** | 0.62 |
|  |  |  |  |  |  |  |  |  |
|  | SCZ PRS | KSADS - ADHD | 1.01 | 0.07 | 0.88 - 1.14 | 0.926 | 0.969 | 0.00 |
|  |  | CBCL - ADHD | 0.97 | 0.09 | 0.81 - 1.16 | 0.721 | 0.969 | 0.01 |
|  |  | KSADS - Anxiety Disorders | 1.03 | 0.08 | 0.88 - 1.21 | 0.737 | 0.969 | 0.01 |
|  |  | KSADS - Anxiety disorders w/ PTSD | 1.00 | 0.08 | 0.86 - 1.17 | 0.969 | 0.969 | 0.00 |
|  |  | KSADS - Depressive Disorders | 0.88 | 0.10 | 0.73 - 1.07 | 0.202 | 0.346 | 0.10 |
|  |  | KSADS - Conduct Disorder | 1.01 | 0.08 | 0.87 - 1.17 | 0.866 | 0.969 | 0.00 |
|  |  |  |  |  |  |  |  |  |
| American Admixed | Family History | KSADS - ADHD | 1.20 | 0.06 | 1.06 - 1.35 | 0.003 | **0.011** | 0.74 |
|  |  | CBCL - ADHD | 1.23 | 0.08 | 1.06 - 1.43 | 0.007 | **0.021** | 0.69 |
|  |  | KSADS - Anxiety Disorders | 1.25 | 0.07 | 1.10 - 1.42 | <0.001 | **0.007** | 1.12 |
|  |  | KSADS - Anxiety disorders w/ PTSD | 1.24 | 0.07 | 1.09 - 1.40 | 0.001 | **0.007** | 0.99 |
|  |  | KSADS - Depressive Disorders | 1.13 | 0.09 | 0.95 - 1.35 | 0.153 | 0.263 | 0.15 |
|  |  | KSADS - Conduct Disorder | 1.16 | 0.07 | 1.01 - 1.33 | 0.041 | 0.082 | 0.36 |
|  |  |  |  |  |  |  |  |  |
|  | SCZ PRS | KSADS - ADHD | 0.95 | 0.07 | 0.82 - 1.10 | 0.468 | 0.624 | 0.04 |
|  |  | CBCL - ADHD | 0.91 | 0.11 | 0.74 - 1.13 | 0.406 | 0.609 | 0.06 |
|  |  | KSADS - Anxiety Disorders | 1.05 | 0.09 | 0.87 - 1.25 | 0.619 | 0.678 | 0.02 |
|  |  | KSADS - Anxiety disorders w/ PTSD | 1.02 | 0.09 | 0.85 - 1.22 | 0.837 | 0.837 | 0.00 |
|  |  | KSADS - Depressive Disorders | 1.36 | 0.12 | 1.07 - 1.73 | 0.011 | **0.028** | 0.47 |
|  |  | KSADS - Conduct Disorder | 0.95 | 0.10 | 0.79 - 1.15 | 0.622 | 0.678 | 0.02 |

Table S13. Independent associations of schizophrenia polygenic risk scores (SCZ PRS) and a family history of psychosis variable which is adjusted for the total number of first- and second-degree relatives (SCZ FH prop) with dimensional, total cognitive score derived from the NIH-Toolbox, Child Behavior Checklist (CBCL) scores, and prodromal questionnaire (PQB) scores. FDR P refers to the false discovery rate–corrected p value; PVE refers to the percentage of variance explained.

| **Ancestry** | **Genetic Risk Measure** | **Precursor** | **B** | **SE** | **95% CI** | **Nominal P** | **FDR P** | **PVE (%)** |
| --- | --- | --- | --- | --- | --- | --- | --- | --- |
| All | SCZ FH (prop) | NIH Total Cognitive Score | -0.21 | 0.17 | -0.55 - 0.13 | 0.236 | 0.332 | 0.02 |
|  |  | CBCL - Total | 1.07 | 0.12 | 0.84 - 1.31 | <0.01 | **<0.01** | 0.87 |
|  |  | CBCL - Anxious Depressed | 0.36 | 0.06 | 0.24 - 0.49 | <0.01 | **<0.01** | 0.37 |
|  |  | CBCL - Withdrawn | 0.39 | 0.06 | 0.27 - 0.51 | <0.01 | **<0.01** | 0.44 |
|  |  | CBCL - Somatic | 0.42 | 0.06 | 0.30 - 0.55 | <0.01 | **<0.01** | 0.47 |
|  |  | CBCL - Social | 0.48 | 0.05 | 0.39 - 0.58 | <0.01 | **<0.01** | 1.02 |
|  |  | CBCL - Thought | 0.58 | 0.06 | 0.46 - 0.71 | <0.01 | **<0.01** | 0.91 |
|  |  | CBCL - Attention | 0.56 | 0.07 | 0.43 - 0.69 | <0.01 | **<0.01** | 0.76 |
|  |  | CBCL - Rule Breaking | 0.36 | 0.05 | 0.25 - 0.46 | <0.01 | **<0.01** | 0.51 |
|  |  | CBCL - Aggressive | 0.44 | 0.06 | 0.32 - 0.55 | <0.01 | **<0.01** | 0.60 |
|  |  | PQB - Distress Score | 0.42 | 0.11 | 0.20 - 0.63 | <0.01 | **<0.01** | 0.15 |
|  |  |  |  |  |  |  |  |  |
|  | SCZ PRS | NIH Total Cognitive Score | -0.43 | 0.17 | -0.77 - -0.10 | 0.011 | **0.023** | 0.07 |
|  |  | CBCL - Total | 0.08 | 0.12 | -0.16 - 0.31 | 0.520 | 0.636 | 0.00 |
|  |  | CBCL - Anxious Depressed | 0.07 | 0.06 | -0.05 - 0.20 | 0.242 | 0.332 | 0.01 |
|  |  | CBCL - Withdrawn | 0.01 | 0.06 | -0.11 - 0.13 | 0.849 | 0.889 | 0.00 |
|  |  | CBCL - Somatic | -0.09 | 0.06 | -0.22 - 0.04 | 0.170 | 0.282 | 0.02 |
|  |  | CBCL - Social | 0.01 | 0.05 | -0.09 - 0.11 | 0.843 | 0.889 | 0.00 |
|  |  | CBCL - Thought | 0.06 | 0.06 | -0.06 - 0.19 | 0.320 | 0.414 | 0.01 |
|  |  | CBCL - Attention | 0.00 | 0.07 | -0.13 - 0.13 | 0.977 | 0.977 | 0.00 |
|  |  | CBCL - Rule Breaking | 0.07 | 0.05 | -0.03 - 0.17 | 0.179 | 0.282 | 0.02 |
|  |  | CBCL - Aggressive | 0.02 | 0.06 | -0.09 - 0.14 | 0.690 | 0.799 | 0.00 |
|  |  | PQB - Distress Score | 0.15 | 0.11 | -0.06 - 0.37 | 0.168 | 0.282 | 0.02 |
|  |  |  |  |  |  |  |  |  |
| European | SCZ FH (prop) | NIH Total Cognitive Score | -0.72 | 0.28 | -1.28 - -0.16 | 0.012 | **0.023** | 0.12 |
|  |  | CBCL - Total | 0.96 | 0.19 | 0.59 - 1.32 | <0.01 | **<0.01** | 0.46 |
|  |  | CBCL - Anxious Depressed | 0.26 | 0.11 | 0.05 - 0.47 | 0.013 | **0.023** | 0.11 |
|  |  | CBCL - Withdrawn | 0.23 | 0.10 | 0.04 - 0.42 | 0.016 | **0.026** | 0.10 |
|  |  | CBCL - Somatic | 0.42 | 0.10 | 0.21 - 0.62 | <0.01 | **<0.01** | 0.29 |
|  |  | CBCL - Social | 0.41 | 0.08 | 0.25 - 0.56 | <0.01 | **<0.01** | 0.48 |
|  |  | CBCL - Thought | 0.66 | 0.10 | 0.46 - 0.86 | <0.01 | **<0.01** | 0.72 |
|  |  | CBCL - Attention | 0.70 | 0.10 | 0.49 - 0.90 | <0.01 | **<0.01** | 0.79 |
|  |  | CBCL - Rule Breaking | 0.34 | 0.08 | 0.19 - 0.50 | <0.01 | **<0.01** | 0.35 |
|  |  | CBCL - Aggressive | 0.37 | 0.09 | 0.19 - 0.55 | <0.01 | **<0.01** | 0.28 |
|  |  | PQB - Distress Score | 0.49 | 0.16 | 0.17 - 0.80 | 0.002 | **0.005** | 0.17 |
|  |  |  |  |  |  |  |  |  |
|  | SCZ PRS | NIH Total Cognitive Score | -0.78 | 0.22 | -1.21 - -0.34 | <0.01 | **0.001** | 0.22 |
|  |  | CBCL - Total | 0.33 | 0.15 | 0.04 - 0.62 | 0.026 | **0.035** | 0.09 |
|  |  | CBCL - Anxious Depressed | 0.21 | 0.08 | 0.04 - 0.37 | 0.013 | **0.023** | 0.11 |
|  |  | CBCL - Withdrawn | 0.09 | 0.08 | -0.06 - 0.24 | 0.238 | 0.275 | 0.02 |
|  |  | CBCL - Somatic | -0.08 | 0.08 | -0.24 - 0.08 | 0.342 | 0.376 | 0.02 |
|  |  | CBCL - Social | 0.02 | 0.06 | -0.09 - 0.14 | 0.683 | 0.683 | 0.00 |
|  |  | CBCL - Thought | 0.13 | 0.08 | -0.03 - 0.29 | 0.102 | 0.124 | 0.05 |
|  |  | CBCL - Attention | 0.07 | 0.08 | -0.09 - 0.23 | 0.384 | 0.403 | 0.01 |
|  |  | CBCL - Rule Breaking | 0.21 | 0.06 | 0.09 - 0.33 | 0.001 | **0.001** | 0.21 |
|  |  | CBCL - Aggressive | 0.16 | 0.07 | 0.02 - 0.30 | 0.023 | **0.034** | 0.09 |
|  |  | PQB - Distress Score | 0.22 | 0.12 | -0.03 - 0.46 | 0.083 | 0.107 | 0.05 |
|  |  |  |  |  |  |  |  |  |
| African | SCZ FH (prop) | NIH Total Cognitive Score | -0.09 | 0.25 | -0.57 - 0.39 | 0.716 | 0.750 | 0.01 |
|  |  | CBCL - Total | 1.19 | 0.19 | 0.82 - 1.57 | <0.01 | **<0.01** | 1.83 |
|  |  | CBCL - Anxious Depressed | 0.42 | 0.08 | 0.25 - 0.58 | <0.01 | **<0.01** | 1.17 |
|  |  | CBCL - Withdrawn | 0.47 | 0.10 | 0.28 - 0.66 | <0.01 | **<0.01** | 1.12 |
|  |  | CBCL - Somatic | 0.42 | 0.09 | 0.23 - 0.60 | <0.01 | **<0.01** | 0.93 |
|  |  | CBCL - Social | 0.55 | 0.08 | 0.39 - 0.71 | <0.01 | **<0.01** | 2.23 |
|  |  | CBCL - Thought | 0.60 | 0.10 | 0.41 - 0.79 | <0.01 | **<0.01** | 1.81 |
|  |  | CBCL - Attention | 0.47 | 0.11 | 0.26 - 0.68 | <0.01 | **<0.01** | 0.91 |
|  |  | CBCL - Rule Breaking | 0.33 | 0.10 | 0.14 - 0.52 | 0.001 | **0.001** | 0.57 |
|  |  | CBCL - Aggressive | 0.49 | 0.10 | 0.30 - 0.68 | <0.01 | **<0.01** | 1.17 |
|  |  | PQB - Distress Score | 0.35 | 0.20 | -0.03 - 0.73 | 0.075 | 0.164 | 0.15 |
|  |  |  |  |  |  |  |  |  |
|  | SCZ PRS | NIH Total Cognitive Score | 0.33 | 0.42 | -0.49 - 1.15 | 0.431 | 0.678 | 0.03 |
|  |  | CBCL - Total | -0.17 | 0.33 | -0.82 - 0.48 | 0.600 | 0.744 | 0.01 |
|  |  | CBCL - Anxious Depressed | -0.06 | 0.15 | -0.35 - 0.23 | 0.676 | 0.744 | 0.01 |
|  |  | CBCL - Withdrawn | -0.19 | 0.17 | -0.52 - 0.14 | 0.267 | 0.500 | 0.06 |
|  |  | CBCL - Somatic | -0.07 | 0.16 | -0.39 - 0.25 | 0.670 | 0.744 | 0.01 |
|  |  | CBCL - Social | -0.01 | 0.14 | -0.28 - 0.26 | 0.946 | 0.946 | 0.00 |
|  |  | CBCL - Thought | -0.07 | 0.17 | -0.40 - 0.25 | 0.657 | 0.744 | 0.01 |
|  |  | CBCL - Attention | 0.13 | 0.18 | -0.23 - 0.49 | 0.475 | 0.697 | 0.02 |
|  |  | CBCL - Rule Breaking | -0.18 | 0.17 | -0.50 - 0.14 | 0.273 | 0.500 | 0.06 |
|  |  | CBCL - Aggressive | -0.13 | 0.17 | -0.47 - 0.20 | 0.431 | 0.678 | 0.03 |
|  |  | PQB - Distress Score | 0.18 | 0.34 | -0.48 - 0.84 | 0.598 | 0.744 | 0.01 |
|  |  |  |  |  |  |  |  |  |
| American Admixed | SCZ FH (prop) | NIH Total Cognitive Score | 0.51 | 0.45 | -0.38 - 1.40 | 0.258 | 0.405 | 0.09 |
|  |  | CBCL - Total | 0.76 | 0.31 | 0.16 - 1.36 | 0.013 | **0.049** | 0.42 |
|  |  | CBCL - Anxious Depressed | 0.35 | 0.16 | 0.03 - 0.66 | 0.032 | 0.088 | 0.31 |
|  |  | CBCL - Withdrawn | 0.46 | 0.17 | 0.12 - 0.80 | 0.008 | **0.035** | 0.48 |
|  |  | CBCL - Somatic | 0.39 | 0.17 | 0.05 - 0.73 | 0.026 | 0.083 | 0.34 |
|  |  | CBCL - Social | 0.40 | 0.13 | 0.15 - 0.66 | 0.002 | **0.024** | 0.64 |
|  |  | CBCL - Thought | 0.29 | 0.16 | -0.01 - 0.59 | 0.061 | 0.149 | 0.24 |
|  |  | CBCL - Attention | 0.47 | 0.17 | 0.14 - 0.80 | 0.005 | **0.027** | 0.54 |
|  |  | CBCL - Rule Breaking | 0.44 | 0.13 | 0.19 - 0.68 | 0.001 | **0.012** | 0.82 |
|  |  | CBCL - Aggressive | 0.43 | 0.15 | 0.14 - 0.72 | 0.004 | **0.026** | 0.58 |
|  |  | PQB - Distress Score | 0.54 | 0.32 | -0.09 - 1.16 | 0.094 | 0.207 | 0.19 |
|  |  |  |  |  |  |  |  |  |
|  | SCZ PRS | NIH Total Cognitive Score | 0.11 | 0.45 | -0.76 - 0.99 | 0.803 | 0.844 | 0.00 |
|  |  | CBCL - Total | 0.33 | 0.31 | -0.29 - 0.94 | 0.296 | 0.417 | 0.07 |
|  |  | CBCL - Anxious Depressed | 0.17 | 0.16 | -0.15 - 0.49 | 0.304 | 0.417 | 0.07 |
|  |  | CBCL - Withdrawn | 0.24 | 0.18 | -0.11 - 0.58 | 0.176 | 0.322 | 0.13 |
|  |  | CBCL - Somatic | 0.14 | 0.18 | -0.21 - 0.49 | 0.428 | 0.523 | 0.04 |
|  |  | CBCL - Social | 0.21 | 0.13 | -0.05 - 0.47 | 0.109 | 0.219 | 0.18 |
|  |  | CBCL - Thought | 0.15 | 0.16 | -0.16 - 0.46 | 0.345 | 0.446 | 0.06 |
|  |  | CBCL - Attention | -0.04 | 0.17 | -0.38 - 0.29 | 0.806 | 0.844 | 0.00 |
|  |  | CBCL - Rule Breaking | 0.16 | 0.13 | -0.09 - 0.41 | 0.213 | 0.360 | 0.11 |
|  |  | CBCL - Aggressive | 0.09 | 0.15 | -0.21 - 0.38 | 0.557 | 0.645 | 0.02 |
|  |  | PQB - Distress Score | 0.04 | 0.32 | -0.59 - 0.68 | 0.890 | 0.890 | 0.00 |

Table S14. Independent associations of schizophrenia polygenic risk scores (SCZ-PRS) a family history of psychosis variable in which is adjusted for the total number of first- and second-degree relatives (SCZ FH prop) with diagnostic phenotypes derived from the Kiddie Schedule for Affective Disorders and Schizophrenia (KSADS) or Child Behavior Checklist (CBCL). FDR P refers to the false discovery rate–corrected p value; PVE refers to the percentage of variance explained.

| **Ancestry** | **Genetic Risk Measure** | **Precursor** | **OR** | **SE** | **95% CI** | **Nominal P** | **FDR P** | **PVE (%)** |
| --- | --- | --- | --- | --- | --- | --- | --- | --- |
| All | SCZ FH (prop) | KSADS - ADHD | 1.13 | 0.02 | 1.08 - 1.18 | <0.01 | **<0.01** | 0.37 |
|  |  | CBCL - ADHD | 1.19 | 0.03 | 1.13 - 1.25 | <0.01 | **<0.01** | 0.68 |
|  |  | KSADS - Anxiety Disorders | 1.18 | 0.02 | 1.13 - 1.24 | <0.01 | **<0.01** | 0.71 |
|  |  | KSADS - Anxiety disorders w/ PTSD | 1.19 | 0.02 | 1.13 - 1.24 | <0.01 | **<0.01** | 0.78 |
|  |  | KSADS - Depressive Disorders | 1.12 | 0.03 | 1.05 - 1.19 | <0.01 | **<0.01** | 0.18 |
|  |  | KSADS - Conduct Disorder | 1.10 | 0.03 | 1.05 - 1.15 | <0.01 | **<0.01** | 0.18 |
|  |  |  |  |  |  |  |  |  |
|  | SCZ PRS | KSADS - ADHD | 1.03 | 0.03 | 0.98 - 1.08 | 0.293 | 0.440 | 0.01 |
|  |  | CBCL - ADHD | 0.96 | 0.04 | 0.88 - 1.04 | 0.277 | 0.440 | 0.02 |
|  |  | KSADS - Anxiety Disorders | 1.03 | 0.03 | 0.97 - 1.09 | 0.356 | 0.455 | 0.01 |
|  |  | KSADS - Anxiety disorders w/ PTSD | 1.03 | 0.03 | 0.97 - 1.09 | 0.379 | 0.455 | 0.01 |
|  |  | KSADS - Depressive Disorders | 1.03 | 0.05 | 0.94 - 1.13 | 0.486 | 0.530 | 0.01 |
|  |  | KSADS - Conduct Disorder | 1.01 | 0.03 | 0.95 - 1.07 | 0.707 | 0.707 | 0.00 |
|  |  |  |  |  |  |  |  |  |
| European | SCZ FH (prop) | KSADS - ADHD | 1.14 | 0.04 | 1.06 - 1.23 | 0.001 | **0.002** | 0.25 |
|  |  | CBCL - ADHD | 1.27 | 0.04 | 1.16 - 1.38 | <0.01 | **<0.01** | 0.84 |
|  |  | KSADS - Anxiety Disorders | 1.15 | 0.04 | 1.07 - 1.24 | <0.01 | **0.001** | 0.30 |
|  |  | KSADS - Anxiety disorders w/ PTSD | 1.15 | 0.04 | 1.07 - 1.24 | <0.01 | **0.001** | 0.30 |
|  |  | KSADS - Depressive Disorders | 1.15 | 0.06 | 1.02 - 1.29 | 0.019 | **0.046** | 0.14 |
|  |  | KSADS - Conduct Disorder | 1.04 | 0.05 | 0.95 - 1.13 | 0.421 | 0.459 | 0.01 |
|  |  |  |  |  |  |  |  |  |
|  | SCZ PRS | KSADS - ADHD | 1.08 | 0.03 | 1.01 - 1.16 | 0.024 | **0.048** | 0.10 |
|  |  | CBCL - ADHD | 1.02 | 0.06 | 0.91 - 1.14 | 0.720 | 0.720 | 0.00 |
|  |  | KSADS - Anxiety Disorders | 1.06 | 0.04 | 0.99 - 1.14 | 0.118 | 0.142 | 0.05 |
|  |  | KSADS - Anxiety disorders w/ PTSD | 1.07 | 0.04 | 0.99 - 1.15 | 0.079 | 0.130 | 0.07 |
|  |  | KSADS - Depressive Disorders | 1.11 | 0.07 | 0.97 - 1.27 | 0.115 | 0.142 | 0.06 |
|  |  | KSADS - Conduct Disorder | 1.07 | 0.04 | 0.99 - 1.15 | 0.086 | 0.130 | 0.06 |
|  |  |  |  |  |  |  |  |  |
| African | SCZ FH (prop) | KSADS - ADHD | 1.11 | 0.03 | 1.03 - 1.18 | 0.003 | **0.009** | 0.50 |
|  |  | CBCL - ADHD | 1.14 | 0.04 | 1.05 - 1.23 | <0.01 | **0.003** | 0.77 |
|  |  | KSADS - Anxiety Disorders | 1.18 | 0.04 | 1.11 - 1.27 | <0.01 | **<0.01** | 1.63 |
|  |  | KSADS - Anxiety disorders w/ PTSD | 1.20 | 0.03 | 1.13 - 1.29 | <0.01 | **<0.01** | 2.03 |
|  |  | KSADS - Depressive Disorders | 1.12 | 0.04 | 1.03 - 1.22 | 0.011 | **0.022** | 0.43 |
|  |  | KSADS - Conduct Disorder | 1.11 | 0.04 | 1.03 - 1.19 | 0.005 | **0.011** | 0.48 |
|  |  |  |  |  |  |  |  |  |
|  | SCZ PRS | KSADS - ADHD | 1.01 | 0.07 | 0.89 - 1.15 | 0.873 | 0.932 | 0.00 |
|  |  | CBCL - ADHD | 0.98 | 0.09 | 0.82 - 1.17 | 0.812 | 0.932 | 0.00 |
|  |  | KSADS - Anxiety Disorders | 1.03 | 0.08 | 0.88 - 1.21 | 0.698 | 0.932 | 0.01 |
|  |  | KSADS - Anxiety disorders w/ PTSD | 1.01 | 0.08 | 0.86 - 1.17 | 0.932 | 0.932 | 0.00 |
|  |  | KSADS - Depressive Disorders | 0.89 | 0.10 | 0.74 - 1.08 | 0.245 | 0.420 | 0.08 |
|  |  | KSADS - Conduct Disorder | 1.02 | 0.07 | 0.88 - 1.18 | 0.806 | 0.932 | 0.00 |
|  |  |  |  |  |  |  |  |  |
| American Admixed | SCZ FH (prop) | KSADS - ADHD | 1.20 | 0.06 | 1.07 - 1.35 | 0.002 | **0.011** | 0.84 |
|  |  | CBCL - ADHD | 1.17 | 0.08 | 1.01 - 1.36 | 0.034 | 0.069 | 0.41 |
|  |  | KSADS - Anxiety Disorders | 1.22 | 0.06 | 1.08 - 1.38 | 0.002 | **0.011** | 0.91 |
|  |  | KSADS - Anxiety disorders w/ PTSD | 1.21 | 0.06 | 1.06 - 1.37 | 0.003 | **0.011** | 0.81 |
|  |  | KSADS - Depressive Disorders | 1.09 | 0.09 | 0.91 - 1.30 | 0.353 | 0.605 | 0.07 |
|  |  | KSADS - Conduct Disorder | 1.21 | 0.07 | 1.06 - 1.38 | 0.004 | **0.011** | 0.76 |
|  |  |  |  |  |  |  |  |  |
|  | SCZ PRS | KSADS - ADHD | 0.95 | 0.07 | 0.82 - 1.10 | 0.503 | 0.670 | 0.04 |
|  |  | CBCL - ADHD | 0.91 | 0.11 | 0.74 - 1.13 | 0.406 | 0.608 | 0.06 |
|  |  | KSADS - Anxiety Disorders | 1.04 | 0.09 | 0.87 - 1.24 | 0.657 | 0.717 | 0.02 |
|  |  | KSADS - Anxiety disorders w/ PTSD | 1.01 | 0.09 | 0.85 - 1.21 | 0.873 | 0.873 | 0.00 |
|  |  | KSADS - Depressive Disorders | 1.37 | 0.12 | 1.08 - 1.74 | 0.008 | **0.020** | 0.53 |
|  |  | KSADS - Conduct Disorder | 0.96 | 0.10 | 0.79 - 1.15 | 0.643 | 0.717 | 0.02 |

**References**

Achenbach, T. M. (1991). *Manual for the Child Behavior Checklist/4-18 and 1991 Profiles.* Burlington, VT: Department of Psychiatry, University of Vermont.

Al‐shoaibi, A. A. A., Zamora, G., Chu, J., Patel, K. P., Ganson, K. T., Testa, A., Jackson, D. B., Tapert, S. F., Baker, F. C., & Nagata, J. M. (2024). Family conflict and less parental monitoring were associated with greater screen time in early adolescence. *Acta Paediatrica*, apa.17349. https://doi.org/10.1111/apa.17349

Barch, D. M., Albaugh, M. D., Avenevoli, S., Chang, L., Clark, D. B., Glantz, M. D., Hudziak, J. J., Jernigan, T. L., Tapert, S. F., Yurgelun-Todd, D., Alia-Klein, N., Potter, A. S., Paulus, M. P., Prouty, D., Zucker, R. A., & Sher, K. J. (2018). Demographic, physical and mental health assessments in the adolescent brain and cognitive development study: Rationale and description. *Developmental Cognitive Neuroscience*, *32*, 55–66. https://doi.org/10.1016/j.dcn.2017.10.010

Barch, D. M., Albaugh, M. D., Baskin-Sommers, A., Bryant, B. E., Clark, D. B., Dick, A. S., Feczko, E., Foxe, J. J., Gee, D. G., Giedd, J., Glantz, M. D., Hudziak, J. J., Karcher, N. R., LeBlanc, K., Maddox, M., McGlade, E. C., Mulford, C., Nagel, B. J., Neigh, G., … Xie, L. (2021). Demographic and mental health assessments in the adolescent brain and cognitive development study: Updates and age-related trajectories. *Developmental Cognitive Neuroscience*, *52*, 101031. https://doi.org/10.1016/j.dcn.2021.101031

Brown, S. A., Brumback, T., Tomlinson, K., Cummins, K., Thompson, W. K., Nagel, B. J., De Bellis, M. D., Hooper, S. R., Clark, D. B., Chung, T., Hasler, B. P., Colrain, I. M., Baker, F. C., Prouty, D., Pfefferbaum, A., Sullivan, E. V., Pohl, K. M., Rohlfing, T., Nichols, B. N., … Tapert, S. F. (2015). The National Consortium on Alcohol and NeuroDevelopment in Adolescence (NCANDA): A Multisite Study of Adolescent Development and Substance Use. *Journal of Studies on Alcohol and Drugs*, *76*(6), 895–908. https://doi.org/10.15288/jsad.2015.76.895

Chang, S. E., Hughes, D. E., Zhu, J., Hyat, M., Salone, S. D., Goodman, Z. T., Roffman, J. L., Karcher, N. R., Hernandez, L. M., Forsyth, J. K., & Bearden, C. E. (2024). Attention-mediated genetic influences on psychotic symptomatology in adolescence. *Nature Mental Health*. https://doi.org/10.1038/s44220-024-00338-7

Conomos, M. P., Laurie, C. A., Stilp, A. M., Gogarten, S. M., McHugh, C. P., Nelson, S. C., Sofer, T., Fernández-Rhodes, L., Justice, A. E., Graff, M., Young, K. L., Seyerle, A. A., Avery, C. L., Taylor, K. D., Rotter, J. I., Talavera, G. A., Daviglus, M. L., Wassertheil-Smoller, S., Schneiderman, N., … Laurie, C. C. (2016). Genetic Diversity and Association Studies in US Hispanic/Latino Populations: Applications in the Hispanic Community Health Study/Study of Latinos. *The American Journal of Human Genetics*, *98*(1), 165–184. https://doi.org/10.1016/j.ajhg.2015.12.001

Conomos, M. P., Reiner, A. P., Weir, B. S., & Thornton, T. A. (2016). Model-free Estimation of Recent Genetic Relatedness. *The American Journal of Human Genetics*, *98*(1), 127–148. https://doi.org/10.1016/j.ajhg.2015.11.022

Gershon, R. C., Wagster, M. V., Hendrie, H. C., Fox, N. A., Cook, K. F., & Nowinski, C. J. (2013). NIH Toolbox for Assessment of Neurological and Behavioral Function. *Neurology*, *80*(Issue 11, Supplement 3), S2–S6. https://doi.org/10.1212/WNL.0b013e3182872e5f

Hair, N. L., Hanson, J. L., Wolfe, B. L., & Pollak, S. D. (2015). Association of Child Poverty, Brain Development, and Academic Achievement. *JAMA Pediatrics*, *169*(9), 822. https://doi.org/10.1001/jamapediatrics.2015.1475

Kaufman, J., Birmaher, B., Brent, D., Rao, U., Flynn, C., Moreci, P., Williamson, D., & Ryan, N. (1997). Schedule for Affective Disorders and Schizophrenia for School-Age Children-Present and Lifetime Version (K-SADS-PL): Initial Reliability and Validity Data. *Journal of the American Academy of Child & Adolescent Psychiatry*, *36*(7), 980–988. https://doi.org/10.1097/00004583-199707000-00021

Rice, S. M., Purcell, R., De Silva, S., Mawren, D., McGorry, P. D., & Parker, A. G. (2016). The Mental Health of Elite Athletes: A Narrative Systematic Review. *Sports Medicine*, *46*(9), 1333–1353. https://doi.org/10.1007/s40279-016-0492-2

The International HapMap 3 Consortium. (2010). Integrating common and rare genetic variation in diverse human populations. *Nature*, *467*(7311), 52–58. https://doi.org/10.1038/nature09298

Townsend, L., Kobak, K., Kearney, C., Milham, M., Andreotti, C., Escalera, J., Alexander, L., Gill, M. K., Birmaher, B., Sylvester, R., Rice, D., Deep, A., & Kaufman, J. (2020). Development of Three Web-Based Computerized Versions of the Kiddie Schedule for Affective Disorders and Schizophrenia Child Psychiatric Diagnostic Interview: Preliminary Validity Data. *Journal of the American Academy of Child & Adolescent Psychiatry*, *59*(2), 309–325. https://doi.org/10.1016/j.jaac.2019.05.009

Trevino, A. D., Jamil, B., Su, J., Aliev, F., Elam, K. K., & Lemery-Chalfant, K. (2024). Alcohol Use Disorder Polygenic Risk Scores and Trajectories of Early Adolescent Externalizing Behaviors: Examining the Role of Parenting and Family Conflict in the Racially/Ethnically Diverse ABCD Sample. *Behavior Genetics*, *54*(1), 101–118. https://doi.org/10.1007/s10519-023-10155-w

Weintraub, S., Dikmen, S. S., Heaton, R. K., Tulsky, D. S., Zelazo, P. D., Bauer, P. J., Carlozzi, N. E., Slotkin, J., Blitz, D., Wallner-Allen, K., Fox, N. A., Beaumont, J. L., Mungas, D., Nowinski, C. J., Richler, J., Deocampo, J. A., Anderson, J. E., Manly, J. J., Borosh, B., … Gershon, R. C. (2013). Cognition assessment using the NIH Toolbox. *Neurology*, *80*(Issue 11, Supplement 3), S54–S64. https://doi.org/10.1212/WNL.0b013e3182872ded
